# Palladium-Catalysed Synthesis and Transformation of Quinolones

**DOI:** 10.3390/molecules24020228

**Published:** 2019-01-09

**Authors:** Vera L. M. Silva, Artur M. S. Silva

**Affiliations:** Department of Chemistry & QOPNA and LAQV-REQUIMTE, Campus Universitário de Santiago, University of Aveiro, 3810-193 Aveiro, Portugal; artur.silva@ua.pt

**Keywords:** palladium, Pd-catalysed, transition-metal catalysis, quinolones, heterocycles, reaction mechanism

## Abstract

Palladium-catalysed reactions have had a large impact on synthetic organic chemistry and have found many applications in target-oriented synthesis. Their widespread use in organic synthesis is due to the mild conditions associated with the reactions together with their tolerance of a wide range of functional groups. Moreover, these types of reactions allow the rapid construction of complex molecules through multiple bond-forming reactions in a single step, the so-called tandem processes. Pd-catalysed reactions have been applied to the synthesis of a large number of natural products and bioactive compounds, some of them of complex molecular structures. This review article aims to present an overview of the most important Pd-catalysed reactions employed in the synthesis and transformations of quinolin-2(1*H*)-ones and quinolin-4(1*H*)-ones. These compounds are widely recognized by their diverse bioactivity, being privileged structures in medicinal chemistry and useful structural moieties for the development of new drug candidates. Furthermore, they hold significant interest due to their host–guest chemistry; applications in chemical, biochemical and environmental analyses and use in the development of new synthetic methods. In some cases, the quinolone formation step cannot be ascribed to a claimed Pd-catalysed reaction but this reaction is crucial to get the appropriate substrate for cyclization into the quinolone. Herein we present and discuss different economical, efficient and selective synthetic strategies to access quinolone-type compounds.

## 1. Introduction

**Figure 1:**
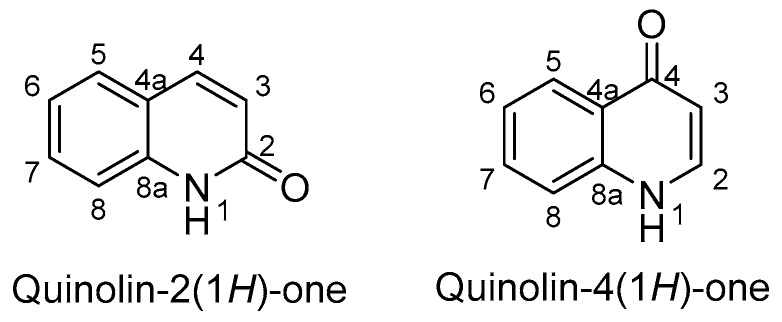
Structures of quinolin-2(1*H*)-one and quinolin-4(1*H*)-one.

Quinolin-2(1*H*)-ones and the isomeric quinolin-4(1*H*)-ones are benzo-α- and benzo-γ-pyridones, respectively, since they are constituted by a α- or γ-pyridone *o*-fused with a benzene ring (Figure 1). The quinolone motif is widely distributed in nature, being particularly found in alkaloids of *Rutaceae* family but can also be produced by different animal and bacterial species [1,2]. These compounds are privileged scaffolds in medicinal chemistry and are ubiquitous substructures associated with relevant biologically active natural products. For instance, quinolin-2(1*H*)-ones were reported as antiulcer (e.g., rebamipide), antihistaminic (e.g., repirinast) [3] and anticancer (e.g., tipifarnib) [4] agents and have also demonstrated activity as antivirals [5], for instance as inhibitors of HIV-1 reverse transcriptase [6,7]. In addition, they are useful intermediates in organic synthesis. Quinolin-4(1*H*)-ones are well-known as antibiotics (e.g., fluoroquinolones) and exhibit excellent antimicrobial activity (e.g., [8]). Moreover quinolin-4(1*H*)-ones possess other interesting biological properties such as antimalarial [9] and antitumoral [10,11] activities, among others. Up to now several methods for the synthesis of quinolones have been reported in the literature but the most commonly used involve the condensation of anilines with β-ketoesters followed by cyclization to give quinolin-2(1*H*)-ones (Conrad–Limpach–Knorr synthesis) or quinolin-4(1*H*)-ones (Conrad–Limpach synthesis). Other classical methods leading to the formation of the C3–C4 bond include the Friedlander synthesis, Camps modification and Niementowski reaction [12,13,14]. Most of these conventional methods often require harsh conditions, tedious workup and purification procedures, are regioselectivity compromised and the variety of substrates is limited. The diversity of the quinolone-containing structures encountered, as well as their biological and pharmaceutical relevance, have motivated research aimed at the development of new economical, efficient and selective synthetic strategies to access these compounds. Recently transition metal-catalysed (TM-catalysed) procedures for the synthesis of such compounds and further transformations have been developed providing increased tolerance toward functional groups and leading generally to higher reaction yields [15]. Many of these methods have proven to be the most powerful and are currently applied in target- or diversity-oriented syntheses. Among TM-catalysed reactions, Pd-catalysed reactions, especially cross-coupling reactions, have found wide application in organic synthesis. The use of Pd-catalysts to allow the rapid construction of complex molecules through multiple bond-forming reactions in a single step, the so-called tandem process, became a powerful tool for synthetic organic chemists. Such processes reduce the steps in the synthesis of certain molecules; thus, being attractive from the viewpoint of developing environmentally benign and economical synthetic methods. This review article is an attempt to compile the most important Pd-catalysed reactions that have been applied in the synthesis and transformation of quinolin-2(1*H*)-ones and quinolin-4(1*H*)-ones in the period ranging from 1990-2017. When describing the developments in this research field, emphasis will be given to the reaction conditions, including the catalysts, ligands and bases, the reaction’s scope, selectivity and in some cases to the mechanism.

## 2. Synthesis of Quinolin-2(1*H*)-ones

### 2.1. Heck Reaction

**Scheme 1:**
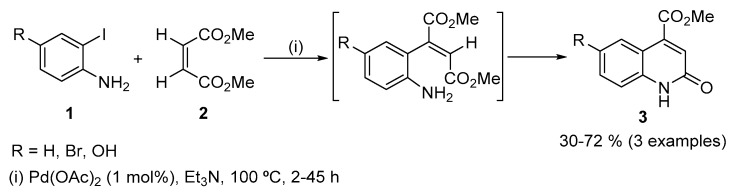
Synthesis of quinolin-2(1*H*)-ones **3** by Heck reaction of 2-iodoanilines **1** with dimethyl maleate **2** [16].

In 1978, Heck and co-workers described for the first time the synthesis of quinolin-2(1*H*)-ones from vinylic substitution of 1,2-disubstituted olefins with 2-iodoanilines [16]. Reaction of 2-iodoanilines **1** with dimethyl maleate **2** using Pd(OAc)_2_ as catalyst and Et_3_N as base, in acetonitrile at 100 °C, afforded as expected from previous reaction with 4-iodoaniline, the *Z* intermediate amino esters which in situ cyclize to quinolin-2(1*H*)-ones **3** in moderate to good yields (72, 55 and 30% for R = H, Br and OH, respectively) (Scheme 1). However, with diethyl fumarate the *E* intermediate amino ester was expected and cyclization should not occur without first isomerizing, since the β-carboxymethyl group was far from the amino group, although quinolin-2(1*H*)-one **3** was isolated in 47% yield along with 20% of aniline. In this case, isomerization of the intermediate should occur fairly easily or the σ-bonded Pd intermediate cyclizes readily in this reaction.

4-Phenylquinolin-2(1*H*)-one was obtained in 66% yield by reacting 2-iodoaniline with (*Z*)-*N*-phenylcinnamamide [16]. As previously referred, the use of (*E*)-*N*-phenylcinnamamide instead of the (*Z*)-isomer also gives the quinolone but only in 15% yield. An alternative route to prepare 4-phenylquinolin-2(1*H*)-one was the reaction of (*E*)-2-aminocinnamic acid (or esters or amides) with iodobenzene, which gives the expected quinolin-2(1*H*)-one in good yield (71%).

**Scheme 2:**
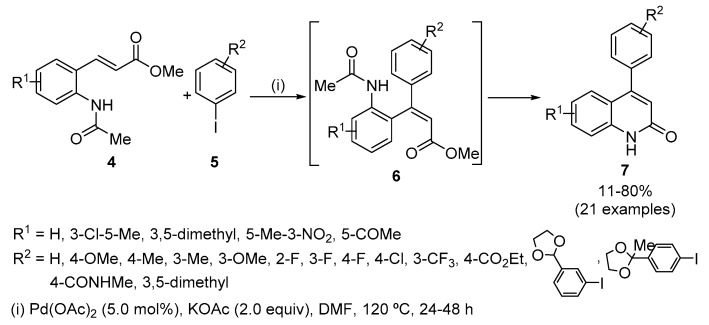
Synthesis of 4-arylquinolin-2(1*H*)-ones **7** through a domino Heck/cyclization of methyl β-(2-acetamidophenyl)acrylates **4** with aryl iodides **5** [17].

Cacchi and co-workers reported a straightforward domino Heck/cyclization reaction of methyl β-(2-acetamidophenyl)acrylates **4** with aryl iodides **5** in the presence of Pd(OAc)_2_ and KOAc in *N*,*N*-dimethylformamide (DMF) at 120 °C that afforded free NH 4-arylquinolin-2(1*H*)-ones **7** in 11–80% yield (Scheme 2) [17]. The vinylic substitution intermediate **6** was only observed when the reaction was performed at lower temperatures (25% at 80 °C and 30% at 60 °C).

**Scheme 3:**
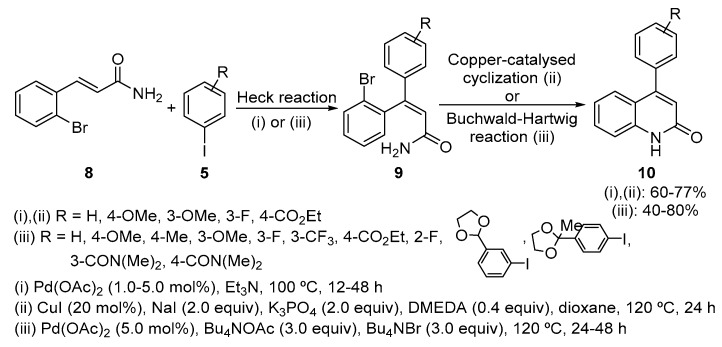
Synthesis of 4-arylquinolin-2(1*H*)-ones **10** through a sequential Heck/Cu-catalysed cyclization process (i, ii) or through a pseudo-domino Heck/Buchwald-Hartwig process (iii) [18,19].

The same authors demonstrated that β-(2-bromophenyl)acrylamide **8** can also be a good precursor for the synthesis of 4-arylquinolin-2(1*H*)-ones **10** by a sequential Heck-reaction-Cu-catalysed-cyclization, using neutral, electron-rich and electron-poor aryl iodides [Scheme 3, (i) and (ii)]. The optimized cyclization conditions involved CuI (20 mol%), NaI (2.0 equiv), K_3_PO_4_ (2.0 equiv), dimethylethylenediamine (DMEDA) (0.4 equiv) in 1,4-dioxane at 120 °C; the reaction did not work in the absence of CuI [18]. Later, they have shown that 4-arylquinolin-2(1*H*)-ones **10** can be prepared using a pseudo-domino process that involves a two mechanistically independent catalytic cycles, a Heck reaction of 2-bromocinnamamide **8** with aryl iodides **5**, with formation of **9**, followed by an intramolecular Buchwald-Hartwig C-N bond forming reaction [Scheme 3, (iii)] [19]. In the Heck reaction, phosphine-free Pd(OAc)_2_ (0.05 equiv) was used as the pre-catalyst and molten tetrabutylammonium acetate/tetrabutylammonium bromide (3.0 equiv of each one) as the reaction medium, at 120 °C.

**Scheme 4:**
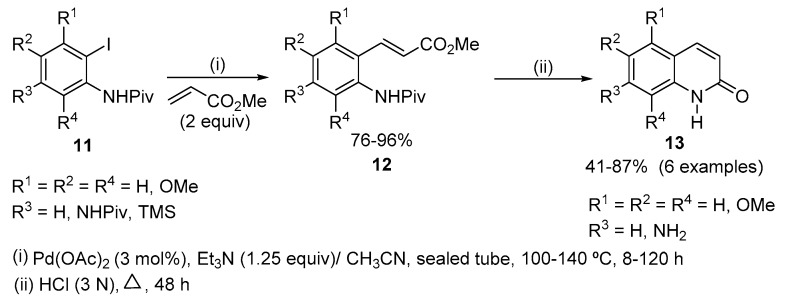
Synthesis of quinolin-2(1*H*)-ones **13** from pivaloylaminobenzenes **11** via Heck coupling and cyclization [20].

Methoxyquinolin-2(1*H*)-ones were synthesized from iodo derivatives of methoxylated pivaloylaminobenzenes **11**, which were obtained via metalation of the corresponding polymethoxypivaloylaminobenzenes with BuLi. The synthesis involves two steps; first the Heck coupling reaction of **11** with methyl acrylate using Pd(OAc)_2_ as catalyst and Et_3_N as base in acetonitrile at 100 °C afforded the corresponding methyl methoxypivaloylaminocinnamates **12** which then undergo cyclization to quinolin-2(1*H*)-ones **13**, in acidic medium, in fairly good overall yields (19–62%) (Scheme 4) [20].

**Scheme 5:**
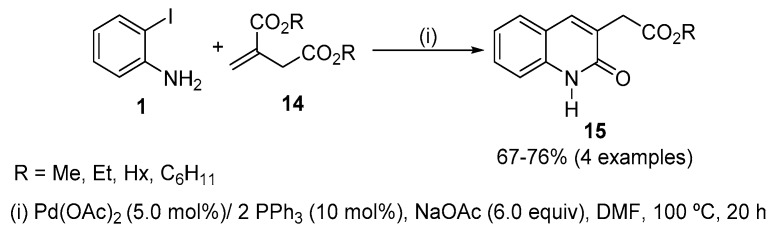
Synthesis of 3-substituted quinolin-2(1*H*)-ones **15** via Heck coupling-cyclization of 2-iodoaniline **1** with α,β-unsaturated carbonyl compounds **14** [21].

The coupling-cyclization between 2-iodoaniline **1** and α,β-unsaturated carbonyl compounds **14** with a catalytic amount of a Pd-catalyst along with a base in DMF at 100 °C afforded 3-substituted quinolin-2(1*H*)-ones **15** in moderate to good yields (67–76%) (Scheme 5) [21]. The best conditions found were 5 mol% Pd(OAc)_2_ as catalyst, 10 mol% PPh_3_ as ligand, NaOAc (6.0 equiv) as base. PdCl_2_(PPh_3_)_2_ was also effective in these conditions. If, a ketone group is linked to the vinyl carbon of the starting alkenes instead of a carboalkoxyl group, 2,3- or 2,4-disubstituted quinolines were obtained, depending if the α- or the β-position of the alkene is substituted.

**Scheme 6:**
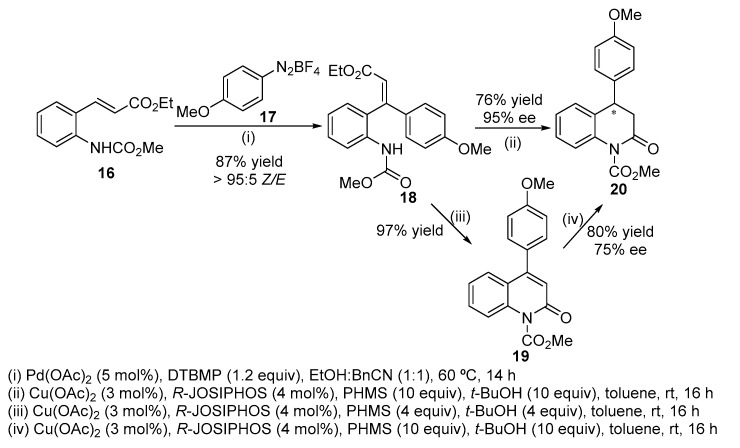
Synthesis of **4**-arylquinolin-2(1*H*)-one **19** via a Pd-catalysed Heck-Matsuda reaction of ester **16** with **17** followed by a Cu-catalysed asymmetric 1,4-reduction/cyclization [22].

A ligand free Pd-catalysed Heck-Matsuda reaction of ethyl cinnamate **16** with arene diazoniumtetrafluoroborate **17** gave β,β-diaryl acrylate **18** which undergo a Cu-catalysed asymmetric 1,4-reduction–cyclization affording 4-methoxyphenylquinolin-2(1*H*)-one **19** [Scheme 6, (i)] [22]. The presence of the base (in this case 2,6-di-*t*-butyl-4-methylpyridine) is important for the excellent stereoselectivity (>95:5 *Z*/*E*) due to the scavenging of PdH, preventing its reinsertion which is responsible for isomerization of Heck adducts. Based on the enantioselective synthesis of β,β-diaryl propanoates by Cu-catalysed 1,4-reduction of β,β-diaryl acrylate using Cu(OAc)_2_ and (*R*)-1-[(*S*)-2-(diphenylphosphino)ferrocenyl]ethyldicyclohexylphosphine (*R*-JOSIPHOS) as ligand, in the presence of an excess of polymethylhydrosiloxane (PMHS), the cyclization of intermediates **18** gave almost quantitatively quinolin-2(1*H*)-one **19** using PHMS/*t*-BuOH (4 equiv) (an intramolecular amidation reaction took place in preference to the 1,4-reduction) [Scheme 6, (iii)]. By using PHMS/*t*-BuOH (10 equiv), the expected dihydroquinolin-2(1*H*)-one **19** is obtained in good yield [Scheme 6, (ii)]. Dihydroquinolin-2(1*H*)-one **20** can also be prepared by subjecting quinolin-2(1*H*)-one **19** to a second Cu-catalysed 1,4-reduction [Scheme 6, (iv)].

**Scheme 7:**
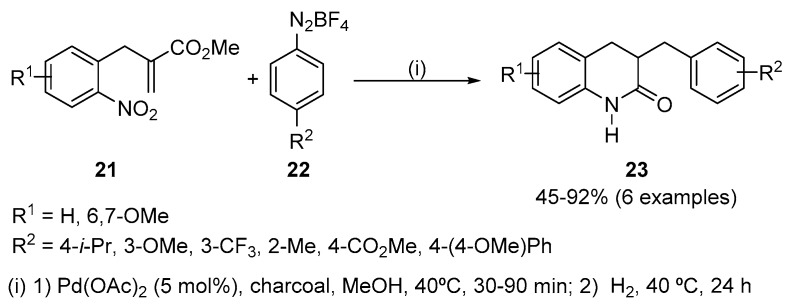
Heterogeneous tandem HRC of 2-(2-nitrophenyl)acrylates **21** with aryldiazonium salts **22**: synthesis of 3,4-dihydroquinolin-2(1*H*)-ones **23** [23].

**Scheme 8:**
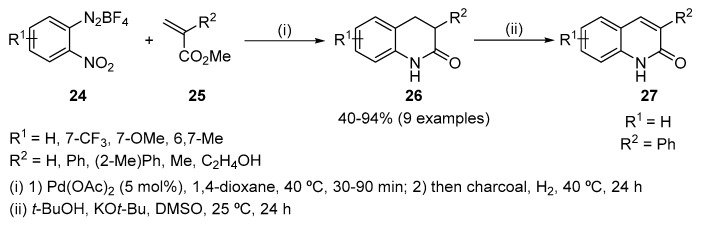
Homogeneous/heterogeneous sequential HRC of 2-nitrobenzenediazonium salts **24** with acrylates **25**: Synthesis of 3,4-dihydroquinolin-2(1*H*)-ones **26** [23].

The selective synthesis of 3,4-dihydroquinolin-2(1*H*)-ones **23** and **26** was achieved following a Pd-catalysed one-pot sequential Heck-reduction-cyclization (HRC) methodology, using either heterogeneous catalysts (Scheme 7) or mixed homogeneous/heterogeneous catalysts (Scheme 8) with Pd^0^/C generated in situ [23,24]. The overall reaction sequence proceeds under mild conditions with good to high isolated yields starting either from 2-(2-nitrophenyl)acrylates **21** and aryldiazonium salts **22** or 2-nitrobenzenediazonium salts **24** and acrylates **25**. Recycling experiments showed that the reused heterogeneous Pd^0^/C catalyst was not able to promote another HRC sequence but was still highly active for hydrogenation reactions. 3,4-Dihydroquinolin-2(1*H*)-ones **26** could be easily dehydrogenated affording quinolin-2(1*H*)-one **27** under mild oxidative conditions.

**Scheme 9:**
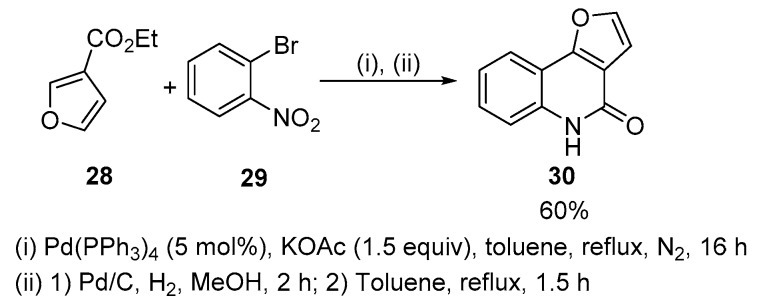
Synthesis of furo[3,2-*c*]quinolin-4(5*H*)-one **30** through two sequential Pd-catalysed reactions of ethyl 3-furoate **28** and 1-bromo-2-nitrobenzene **29** [25].

The arylation of ethyl 3-furoate **28** with 1-bromo-2-nitrobenzene **29** in the presence of Pd(PPh_3_)_4_ afforded ethyl 2-(2-nitrophenyl)furan-3-carboxylate (80%) (Scheme 9) [25]. Hydrogenation of the nitro group with Pd/C as catalyst and subsequent cyclization occurred uneventfully to provide furo[3,2-*c*]quinolin-4(5*H*)-one **30** (75%). An overall yield of 60% was achieved for these two sequential Pd-catalysed reactions.

**Scheme 10:**
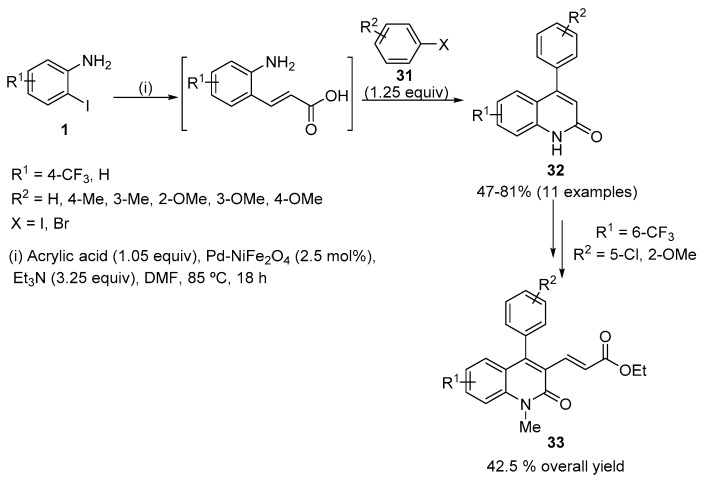
Synthesis of 4-arylquinolin-2(1*H*)-ones **32** by Heck reaction of substituted 2-iodoanilines **1** with acrylic acid followed by in situ cyclization [26].

The successive Heck reaction on substituted 2-iodoaniline **1** with acrylic acid catalysed by Pd supported on nickel ferrite, as a heterogeneous and recyclable catalyst, followed by in situ cyclization afforded 4-arylquinolin-2(1*H*)-ones **32** (Scheme 10) [26]. Regarding the aryl halide **31**, aryl iodides gave better yields of the desired product compared with aryl bromides. The scope of this methodology was extended to the synthesis of bioactive 3-alkenyl derivatives of 4-arylquinolin-2(1*H*)-ones **33** (Scheme 10).

**Scheme 11:**
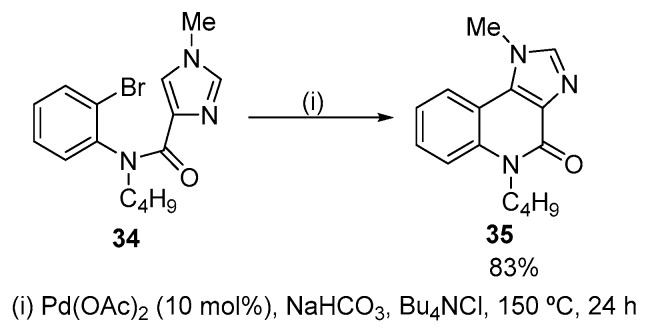
Synthesis of quinolone **35** by an intramolecular Heck cyclization of carboxamide **34** [27].

Pd-catalysed intramolecular Heck cyclization of *N*-phenyl-1*H*-imidazole-4-carboxamide **34** afforded 5-butyl-1-methyl-1*H*-imidazo[4,5-*c*]quinolin-4(5*H*)-one **35** with 83% yield (Scheme 11) [27].

**Scheme 12:**
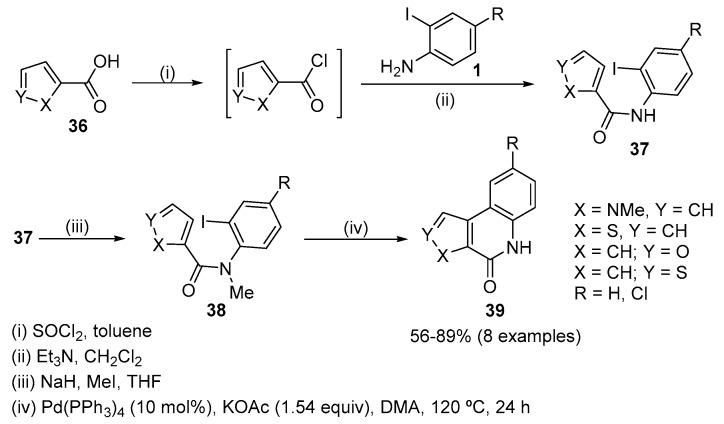
Intramolecular Heck cyclization of *N*-(hetero)arylcarboxamides **38** into quinolones **39** [28].

The intramolecular Heck cyclization of *N*-(hetero)arylcarboxamides afforded tricyclic fused quinolone derivatives (Scheme 12) [28]. These compounds were prepared by treatment of the acyl chlorides, generated in situ from the commercially available pyrrole-, thiophene- and furan-2/3-carboxylic acids **36**, with the appropriate 2-iodoanilines **1**, followed by *N*-methylation of the *N*-(hetero)arylcarboxamides **37** to avoid N-Pd complexation. Intramolecular Heck cyclization of **38** at the positions 2 or 3 of the heterocyclic nucleus with Pd(PPh_3_)_4_ as catalyst and KOAc as base afforded the corresponding tricyclic fused quinolones **39** in moderate to high yields (Scheme 12).

**Scheme 13:**
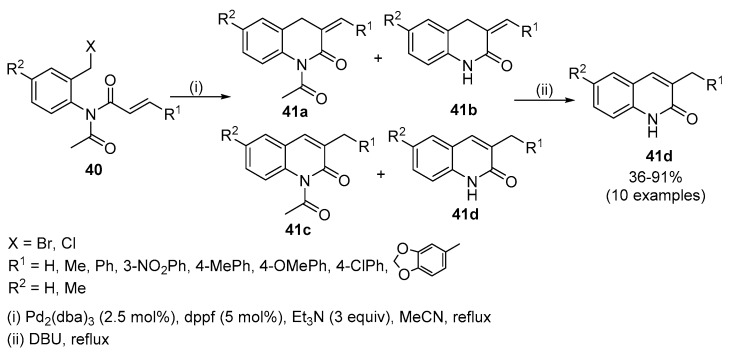
Synthesis of quinolin-2(1*H*)-ones **41d** by intramolecular Heck cyclization of acrylamides **40** [29].

Pd-catalysed intramolecular Heck cyclization of *N*-acetyl-*N*-[2-(halomethyl)aryl]acrylamides **40** afforded 3-alkylquinolin-2(1*H*)-ones **41d** and another three cyclization products **41a**–**c** (Scheme 13) [29]. Compounds **41a** and **41c** are the acetylated products of **41b** and **41d**, respectively. Treatment of the reaction mixture with 1,8-diazabicyclo[5.4.0]undec-7-ene (DBU) successfully converted **41a**–**c** to 3-alkylquinolin-2(1*H*)-one **41d**. Combination of Pd_2_(dba)_3_ and 1,1′-*bis*(diphenylphosphino)ferrocene (dppf) in the presence of Et_3_N in refluxing MeCN followed by treatment of the reaction mixture with DBU led to 3-alkylquinolin-2(1*H*)-one **41d** in good to excellent yields (Scheme 13).

### 2.2. Sonogashira Reaction

**Scheme 14:**
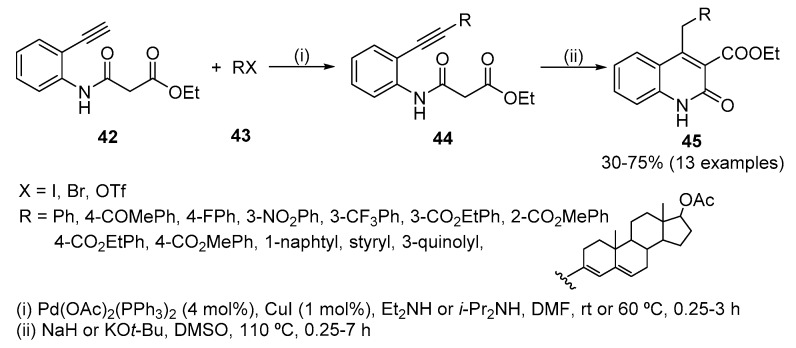
Synthesis of 3,4-disubstituted quinolin-2(1*H*)-ones **48** by a Pd-catalysed reaction of ethyl *N*-(2-ethynyl)malonanilide **45** followed by an intramolecular carbocyclization [30].

Pd-catalysed coupling of ethyl *N*-(2-ethynyl)malonanilide **42** with aryl, heteroaryl, vinyl halides or vinyl triflates **43** gave derivatives **44**, which upon intramolecular cyclization, under basic conditions, afforded 3,4-disubstituted quinolin-2(1*H*)-ones **45** (Scheme 14) [30]. The mechanism of this carbocyclization involves an intramolecular nucleophilic attack of the carbanion, generated from **44**, on the carbon-carbon triple bond giving, after protonation, a six-membered ring methylidene intermediate that isomerizes to the quinolin-2(1*H*)-one **45**. The success of this cyclization step is determined by the nature of the substituent in the acetylenic moiety. Good yields were obtained with aromatic rings bearing electron-withdrawing substituents (60–75%) while no quinolin-2(1*H*)-ones were obtained with electron-donating *p*-methoxyphenyl or butyl groups.

### 2.3. Carbonylative Annulation

**Scheme 15:**
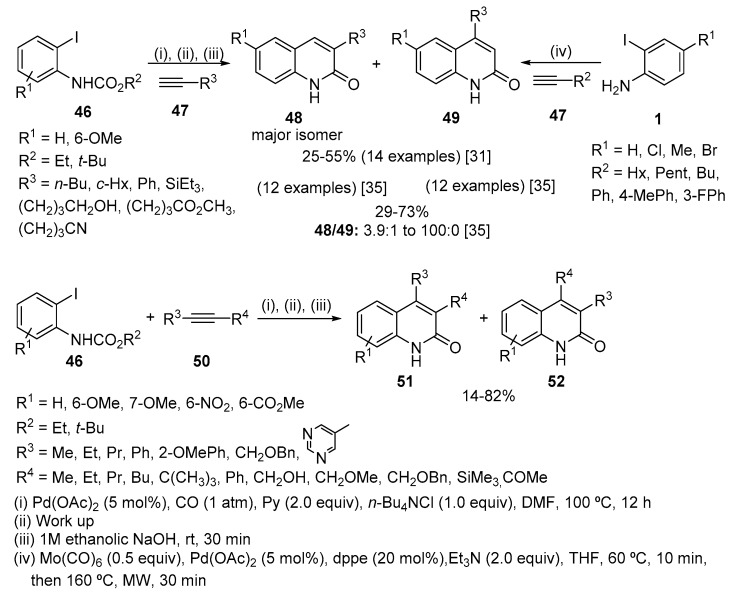
Synthesis of 3- and 4-substituted quinolin-2(1*H*)-ones **48** and **49** and 3,4-disubstituted quinolin-2(1*H*)-ones **51** and **52** via Pd-catalysed carbonylative annulation of 2-iodoanilines **46** and **1** with terminal alkynes and with internal alkynes, respectively [31,34,35].

Both 3- and 4-substituted quinolin-2(1*H*)-ones **48** and **49** were obtained by Pd-catalysed carbonylative annulation of terminal alkynes **47** with 2-iodoaniline derivatives **46**, in the presence of carbon monoxide (CO), pyridine (Py) and Pd(OAc)_2_ (Scheme 15) [31]. Terminal alkynes bearing alkyl, phenyl, silyl, hydroxy, ester and cyano substituents gave quinolin-2(1*H*)-ones in moderate yields. Only the 3-substituted quinolin-2(1*H*)-ones **48** were obtained in the reaction with phenylacetylene and triethylsilylacetylene. Removal of the carbamate-protecting group by treating the crude reaction with 1 M of ethanolic NaOH is necessary to avoid the formation of a mixture of deprotected and protected quinolin-2(1*H*)-ones. Both 3- and 4-substituted quinolin-2(1*H*)-ones **48** and **49** were obtained in the reactions with terminal alkynes bearing long alkyl chains. An increase in the size of the substituent on the triple bond improves the regioselectivity. Furthermore, the ratio of isomers is around 4 to 1 when functionalized acetylenes are used compared to the 2.2 to 1 ratio obtained in most of the reactions with 1-hexyne. The formation of quinolin-2(1*H*)-ones and not quinolin-4(1*H*)-ones shows that the key step in this process is the insertion of the terminal alkyne into the carbon-Pd bond instead of undergoing a Sonogashira-type coupling [32,33]. The authors have conducted an isotope labelling experiment to unambiguously prove this reactivity pattern.

Later, the same authors reported the synthesis of 3,4-disubstituted quinolin-2(1*H*)-ones **51** and **52** by a ligand free Pd-catalysed annulation of internal alkynes **50** with *N*-substituted 2-iodoanilines **46** in the presence of CO (Scheme 15) [34]. The type of the substituent on the nitrogen was fundamental to obtain quinolin-2(1*H*)-ones in high yields. Better yields were obtained using mild electron-withdrawing substituents as *p*-toluenesulfonyl, trifluoroacetyl and alkoxycarbonyl. The nitrogen substituent is lost during the progress of the reaction affording *N*-unsubstituted quinolin-2(1*H*)-ones, except for aminocarbonyl and ethoxycarbonyl groups that originated the corresponding protected quinolin-2(1*H*)-one in 7% and 11% isolated yields, respectively. A wide variety of internal alkynes **50**, bearing alkyl, aryl, heteroaryl, hydroxy and alkoxy substituents, were effective in this reaction; however, unsymmetrical alkynes originated mixtures of regioisomers **51** and **52**, with low regioselectivity, owing to the two possible modes of alkyne insertion into the aryl-Pd bond. Electron-deficient alkynes are very poor substrates for the carbonylative annulation. Electron-rich 2-iodoanilines can react as annulating agents but when the substitution is *para* to the iodine the yield is lower. Carbonylative annulation of electron-poor 2-iodoanilines gives the corresponding quinolin-2(1*H*)-ones in lower yields than the parent system.

Pd-catalysed carbonylative annulation of unprotected 2-iodoanilines **1** and terminal alkynes **47**, using the commercially available molybdenum hexacarbonyl [Mo(CO)_6_] as a convenient and solid CO source, also afforded 3- and 4-substituted quinolin-2(1*H*)-ones **48** and **49**, the latter ones as minor products [Scheme 15, (iv)] [35]. The reactions were conducted at 160 °C for 30 min under microwave irradiation. Et_3_N was the best base in terms of yield and regioselectivity. Pd(OAc)_2_ exhibited good catalytic activity whereas 1,2-*bis*(diphenylphosphino)ethane (dppe) and tetrahydrofuran (THF) were the best ligand and solvent choice for regioselectivity and yields. Different substituted 2-iodoanilines and alkyl alkynes were used without significant loss in reaction yield or efficiency. Aryl-substituted alkynes gave only the 3-substituted quinolin-2(1*H*)-one **48** although the yield was moderate. Neither internal alkynes nor *N*-protected 2-iodoanilines can be used in this carbonylative annulation.

**Scheme 16:**
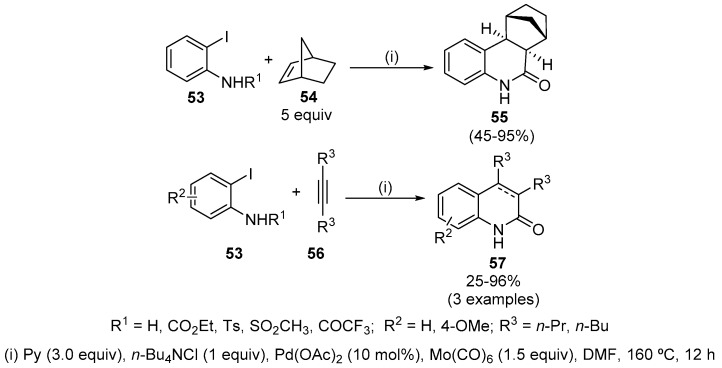
Synthesis of 3,4-disubstituted (dihydro)quinolin-2(1*H*)-ones **55** and **57** by carbonylative annulation of *o*-iodoanilines **53** [36].

Protected iodoanilines **53** reacted with internal alkenes **54** or alkynes **56** and Mo(CO)_6_, affording 3,4-disubstituted (dihydro)quinolin-2(1*H*)-ones **55** and **57** (Scheme 16) [36]. The reaction is ligand-free and avoids the problematic use of gaseous CO. In addition, the annulation reactions proceed with insertion of unsaturated compounds into the arylpalladium bond in preference to insertion of CO where no isomeric quinolones were observed. For instance, norbornene adds to the arylpalladium intermediate in a *cis-exo* manner followed by CO insertion and intramolecular amination (Scheme 16). The slow CO liberation during the reaction also provides an efficient pressure of the gas to promote the carbonylative reaction. This protocol merges three Heck, carbonylation and amination reactions to construct two C-C and one C-N bonds in one pot. It is compatible with various amino protecting groups, such as ethoxycarbonyl, tosyl, sulfonyl and acyl groups; however, deprotection occurred during the reaction and workup procedure, affording the free quinolin-2(1*H*)-one. Nonetheless, it is important to note that the yields are better when using *N*-protected anilines and depend on the protecting group. Reactions of *N*-substituted anilines with dipropyl acetylene, 4-octyne and 5-decyne afforded the annulation product in moderate to very good yields. Unfortunately, diphenylacetylene did not participate in the annulation reaction with *o*-iodoaniline; only traces of the desired product were obtained.

**Scheme 17:**
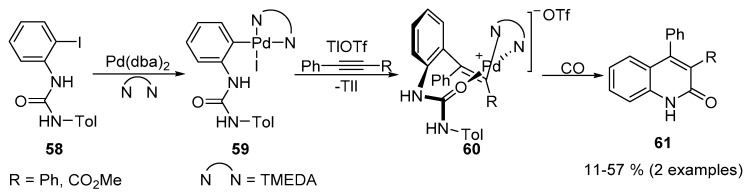
Synthesis of 3-substituted 4-phenylquinolin-2(1*H*)-ones **61** by reaction of vinyl-Pd complexes **60** with CO [37].

The oxidative addition of 1-(2-iodophenyl)-3-*p*-tolylurea **58** to Pd(dba)_2_ in the presence of 1 equiv of *N*,*N*,*N*′,*N*′-tetramethyl-1,2-ethylenediamine (TMEDA) followed by the reaction of **59** with internal alkynes, originated vinyl-Pd complexes **60**. These complexes reacted with CO to give Pd and two 3-substituted 4-phenylquinolin-2(1*H*)-ones **61** (R = Ph, 11% and R = CO_2_Me, 57%) (Scheme 17) [37]. The reaction mechanism should follow CO insertion into the Pd-C-vinyl bond of **60** to give an acyl Pd derivative, which undergoes a C,N coupling with loss of Pd and *p*-tolyl isocyanate.

**Scheme 18:**
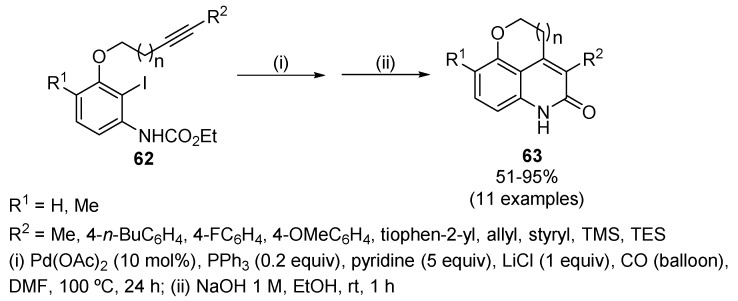
Synthesis of 4,5-fused tricyclic quinolin-2(1*H*)-ones **63** by intramolecular carbonylative annulation of alkyne-tethered *o*-iodoanilines **62** [38].

Following the work of Larock, Jia and co-workers developed a protocol for the efficient synthesis of 4,5-fused tricyclic quinolin-2(1*H*)-ones **63** by intramolecular carbonylative annulation of alkyne-tethered *o*-iodoanilines **62** (Scheme 18) [38]. The use of PPh_3_ as ligand was found to be crucial for this reaction although its role is not clear. For most of the substrates tested, the reaction afforded a mixture of the *N*-protected and free (NH) fused quinolin-2(1*H*)-ones in different ratios. This problem was overwhelmed by treatment of the crude products with 1 M ethanolic NaOH to completely hydrolyse the *N*-substituted product thus obtaining only the expected fused quinolin-2(1*H*)-one **63**. The electronic effect of the aryl groups on the internal alkyne on the reactivity was not significant. In addition, substrates leading to 7- and 8-membered ring fused quinolin-2(1*H*)-ones gave the desired products in good yields and the *o*-methyl substituent on *2*-iodoaniline was well tolerated.

**Scheme 19:**
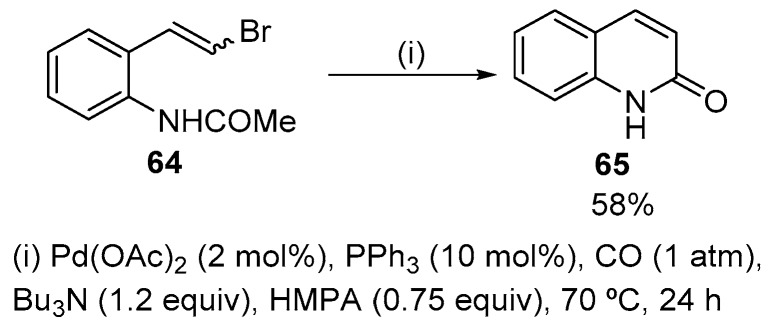
Synthesis of quinolin-2(1*H*)-one **65** by Pd-catalysed carbonylation of vinyl bromides **64** [39].

In 1979, Ban and co-workers described the Pd-catalysed carbonylation of vinyl bromides bearing an internal amide group **64** [39]. In the presence of the catalytic system Pd(OAc)_2_ and PPh_3_, the (*Z*)-isomer **64** of the vinyl bromide smoothly gave quinolin-2(1*H*)-one **65** with loss of the *N*-acetyl group (Scheme 19). In the same reaction conditions, the corresponding (*E*)-isomer gave only 7.7% yield of the same quinolin-2(1*H*)-one **65** because palladation occurs at the position of the halogen atom of the vinyl halide.

**Scheme 20:**
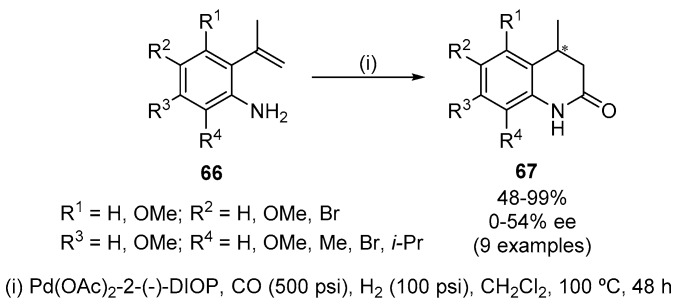
Synthesis of 4-methyl-3,4-dihydroquinolin-2(1*H*)-ones **67** by asymmetric cyclocarbonylation of 2-(1-methylvinyl)anilines **66** [40].

Asymmetric cyclocarbonylation of 2-(1-methylvinyl)anilines **66** using a catalyst system of Pd(OAc)_2_-2(−)-DIOP [2,3-*O*-isopropylidene-2,3-dihydroxy-1,4-*bis*(diphenylphosphino)butane] [Pd(OAc)_2_ (1 mol%), (−)-DIOP (2 mol%)], in CH_2_Cl_2_ at 100 °C for 48 h, under CO (500 psi) and hydrogen pressure (100 psi), originated chiral 4-methyl-3,4-dihydroquinolin-2(1*H*)-ones **67** in up to 54% ee (Scheme 20) [40]. Other chiral ligands [(2*S*,4*S*)-1-*t*-butoxycarbonyl-4-diphenylphosphino-2-(diphenylphosphinomethyl)pyrrolidine, 2,2′-*bis*(diphenylphosphino)-1,1′- binaphthyl, (2*S*,3*S*)-(−)-*bis*(diphenylphosphino)butane] [(−)-bppm, (+)-BINAP and *R*,*R*-BDPP] were inferior to (−)-DIOP in terms of enantioselectivity, which was not influenced by the amount of (−)-DIOP (1, 2, 6 equiv) or Pd-precursors [Pd_2_(dba)_3_.CHCl_3_, Pd(acac)_2_, Pd(CF_3_COO)_2_, п-allyl-Pd chloride dimer] employed. However, it was slightly sensitive to the ratio of CO and hydrogen pressure; an increase of this ratio reduces the enantioselectivity. In the absence of hydrogen, the yield is very low (37%) although the optical purity was still maintained (33% ee). Substituents at the 4- and 5- position of 2-(1-methylvinyl)anilines **66** showed some effect on the enantioselectivity (**67**: R^2^ = R^3^ = OMe, 20% ee; R^2^ = Br, 31% ee). Stronger effects were found in the case of **66**: R^1^ = OMe, the reaction was not enantioselective affording the corresponding racemic product while the carbonylation of **66**: R^4^ = OMe proceeded in a more enantioselective manner affording the corresponding quinolone **67** in nearly quantitative yield (99%, 54% ee). For 6-substituted anilines **66**, excellent yields of **67:** R^4^ = Me (98%) and **67:** R^4^ = *i*-Pr (99%) were obtained whereas the carbonylation of aniline **66:** R^2^ = R^4^ = Br afforded the corresponding 4-methyl-3,4-dihydroquinolin-2(1*H*)-one **67** in moderate yield (48%).

### 2.4. Ullmann Reaction

**Scheme 21:**
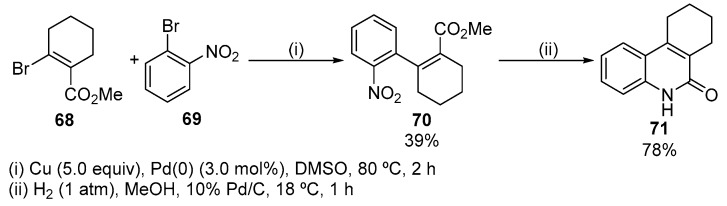
Synthesis of quinolin-2(1*H*)-one **71** via Pd^0^-mediated Ullmann cross-coupling/reductive cyclization sequence [41].

The Pd(0)-mediated Ullmann cross-coupling reaction of β-bromo-ester **68** with 1-bromo-2-nitrobenzene **69** gave the nitro-ester **70** which engaged in reductive cyclization on exposure to hydrogen in the presence of Pd/C affording the quinolin-2(1*H*)-one **71** (Scheme 21) [41].

### 2.5. Buchwald-Hartwig Reaction

**Scheme 22:**
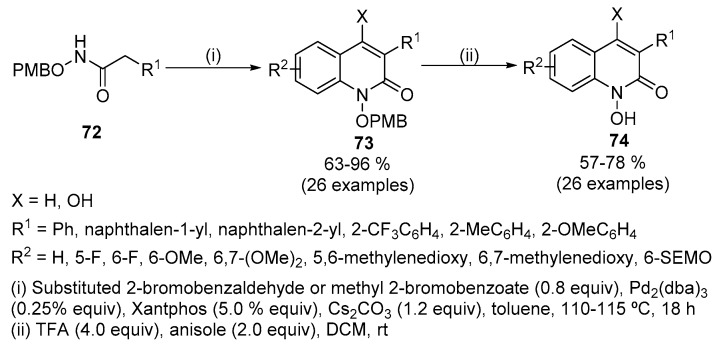
Synthesis of *N*-hydroxyquinolin-2(1*H*)-ones **74** by Pd-catalysed Buchwald-Hartwig reaction of protected *N*-hydroxyamides **72** with benzaldehydes or benzoates followed by cyclodehydration and deprotection [42].

The coupling of the protected *N*-hydroxyamides **72** with benzaldehydes or benzoates by Pd-catalysed Buchwald-type C-N bond formation followed by a tandem cyclodehydration afforded the quinolin-2(1*H*)-ones **73** in good to excellent yields (63–96%) (Scheme 22) [42]. Pd_2_(dba)_3_ was the best catalyst in the presence of Cs_2_CO_3_ and 4,5-*bis*(diphenylphosphino)-9,9-dimethylxanthene (Xantphos) as the ligand. Various aryl and naphthyl substituents were tolerated under the reaction conditions. Treatment of compounds **73** with trifluoroacetic acid (TFA) in dichloromethane afforded the corresponding *N*-hydroxyquinolin-2(1*H*)-ones **74** in 57–78% yield.

**Scheme 23:**
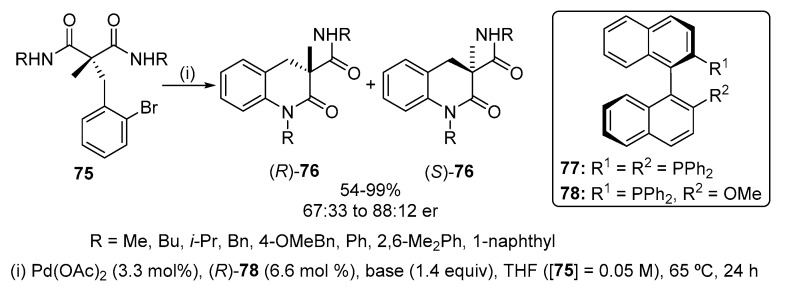
Synthesis of enantiopure 3,4-dihydroquinolin-2(1*H*)-ones **76** by an enantioselective Buchwald-Hartwig reaction [43].

Starting from symmetrical α-(2-bromobenzyl)malonamides **75**, desymmetrized 3,4-dihydroquinolin-2(1*H*)-ones **76** were synthesized in nearly quantitative yields and enantiomeric ratios up to 88:12 by an unusual enantioselective Buchwald-Hartwig reaction (Scheme 23) [43]. Using enantiopure (*R*)-**77** as ligand, **76**: R = Bn was isolated in 85% yield but in a 57:43 er. When (*R*)-**78** was employed in the same reaction, the yield was comparable to that obtained with (*R*)-**77** but enantioselectivity was improved significantly to a 79:21 ratio. Different ratios of Pd to (*R*)-**78** were investigated from 1:1 to 1:3 because this monophosphine may complex Pd differently from other bisphosphines. The yield was slightly lower for 1:3 ratio but the enantioselectivity was nearly the same for all ratios studied. In 1,4-dioxane or THF the product was afforded in excellent yields and good enantiomeric ratios compared to more polar solvents such as MeCN and DMSO. A series of bases were screened using refluxing THF but the best results were obtained with K_3_PO_4_ and Cs_2_CO_3_ affording the product in nearly quantitative yield with good enantioselectivity. While most of quinolin-2(1*H*)-ones **76** were obtained in 99%, more sterically demanding amide nitrogen substituents led to lower reaction yields (*i*-Pr, 90%; 2,6-Me_2_Ph, 54%; 1-naphthyl, 79%), although the enantioselectivity increased with the steric bulk of the substituent (ranging from 69:31 er for Me up to 85:15 er and 88:12 er for *i*-Pr and 4-OMeBn, respectively). In the case of *N*-aryl substrates the same enantiomeric ratios of *N*-phenyl and *N*-benzylquinolin-2(1*H*)-ones (79:21 er) were observed.

**Scheme 24:**
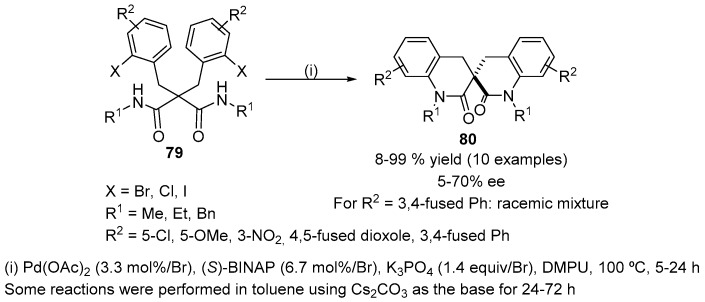
Synthesis of spirobis[3,4-dihydroquinolin-2(1*H*)-ones] **80** by intramolecular double *N*-arylation of malonamides **79** [44].

Enantioselective intramolecular double *N*-arylation of malonamides **79** bearing 2-bromoarylmethyl groups catalysed by a Pd-BINAP complex afforded C2-symmetric spirobis[3,4-dihydroquinolin-2(1*H*)-ones] **80** in 99% yield and up to 70% ee (Scheme 24) [44]. The scope of this reaction was investigated using a variety of malonamides **79** and it was demonstrated that substrates having 2-chlorobenzyl groups were less reactive (8% yield, 6% ee). Although the reaction with the iodide analogues took place smoothly to give quinolin-2(1*H*)-ones **80** in high yield (94%), its enantioselectivity was as low as 38% ee. The reaction enantioselectivity is also affected by the substituent on the aromatic ring, moderate optical purity being obtained for 5-Cl substituents (85% yield, 48% ee) whereas nearly racemic products were produced for 5-OMe- (90% yield, 6% ee) and 3-NO_2_-substituted malonamides (42% yield, 5% ee). It was also possible to introduce a 1,3-benzodioxole ring leading to quantitative formation of the corresponding spirobis[3,4-dihydroquinolin-2(1*H*)-one] (99% yield, 57% ee) and no selectivity was observed for sterically more demanding substrates.

**Scheme 25:**
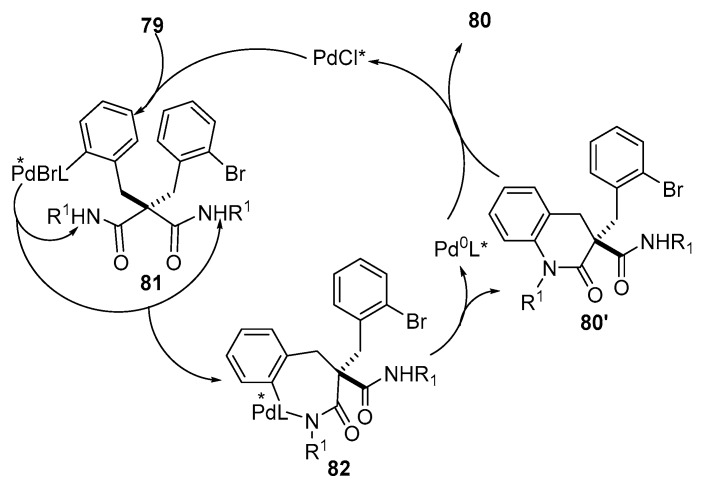
Plausible catalytic cycle [44].

The mechanism proposed for the formation of compounds **80** seems to be initiated by the oxidative addition of one of the bromoarenes in **79** to a catalytically active Pd(0) complex. Then desymmetrisation of the amide groups of the resulting Pd(II) complex **81** occurs affording palladacycle **82**. The subsequent C-N bond-forming reductive elimination yields monocyclized compound **80**′ with regeneration of the Pd catalyst. Then intramolecular *N*-arylation of **80**′ affords the expected spirobis[3,4-dihydroquinolin-2(1*H*)-one] **80**. According to the authors, the enantioselectivity is determined in the first cyclization. They also confirmed that a kinetic resolution process is involved in the second cyclization (Scheme 25) [44].

### 2.6. Intramolecular C-H Alkenylation

**Scheme 26:**
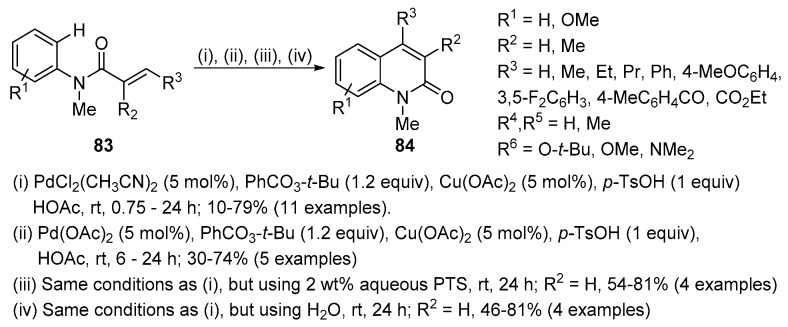
Synthesis of quinolin-2(1*H*)-ones **84** by selective 6-*endo* intramolecular C-H alkenylation of *N*-phenylacrylamides **83** [45].

Pd(II)-catalysed selective 6-*endo* intramolecular C-H alkenylation of *N*-phenylacrylamides **83**, in the conditions shown in Scheme 26, allows the construction of the quinolin-2(1*H*)-one core **84** [45]. In all cases, the quinolin-2(1*H*)-one **84** was the unique isolated product and the formation of the indolin-2-one, due to a 5-*exo* cyclization, was not detected. The reaction was efficient with an unsubstituted alkene and substitution on the 3-position of the acrylamide is well tolerated, leading to 4-substituted quinolin-2(1*H*)-ones. However, the presence of an ester or ketone moiety led to the expected product in low isolated yields, 10% and 22%, respectively. A better yield of 30% was achieved for the ketone but in the absence of Cu(OAc)_2_. 2,3-Disubstituted acrylamides can also be used leading to 3,4-disubstituted quinolin2(1*H*)-ones. *N*-demethylated acrylamides also gave the corresponding quinolin-2(1*H*)-ones but in lower yields. Although acetic acid is a good solvent, the reaction can also be performed in aqueous media at room temperature, using a 2% aqueous solution of polyoxyethanyl-α-tocopheryl sebacate (PTS) or even in water, with good yields but for a prolonged reaction time (24 h).

**Scheme 27:**
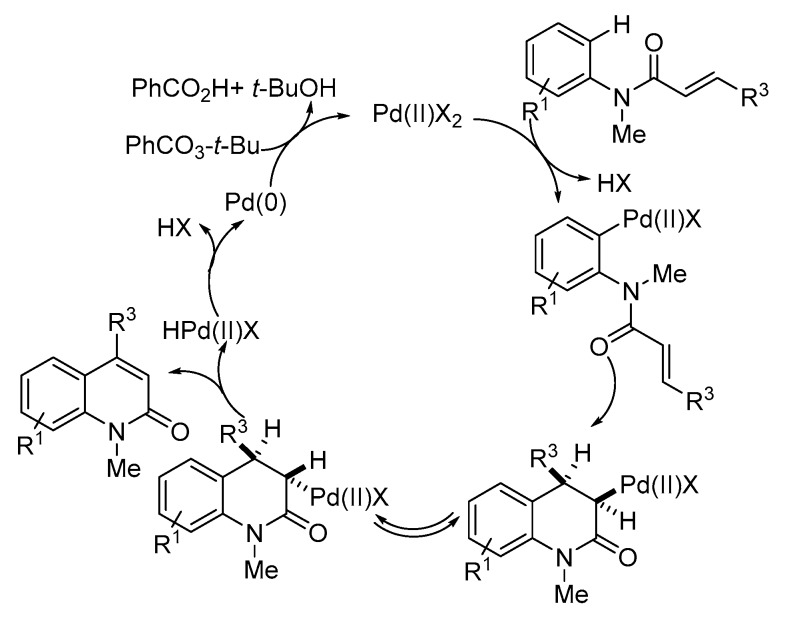
Mechanism proposed for the Pd(II)-catalysed 6-*endo* intramolecular C-H alkenylation [45].

The mechanism of this intramolecular cyclization seems to involve an electrophilic palladation of the aromatic ring to form an aryl-Pd(II) intermediate (Scheme 27). This arylpalladium would undergo a *syn* insertion into the activated alkene moiety in a 6-*endo* mode, followed by β-hydride elimination to afford the quinolone framework. Given the (*E*)-configuration of the starting alkenes, the subsequent *syn* β-hydride elimination would require the prior epimerization of the α-carbon through the formation of an O-Pd intermediate.

### 2.7. Intramolecular Amidation of C(sp^2^)-H Bonds

**Scheme 28:**
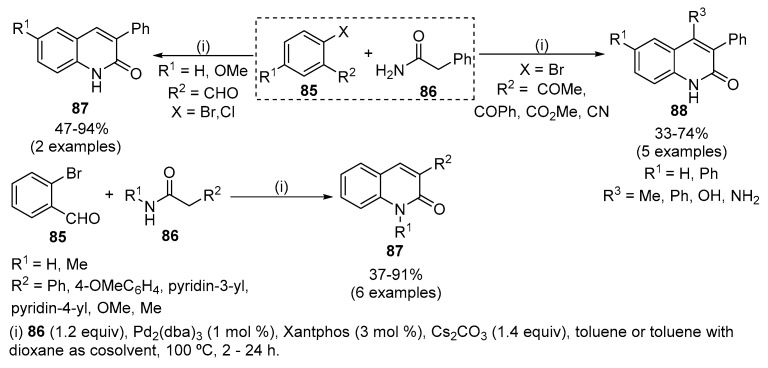
Synthesis of quinolin-2(1*H*)-ones **87** and **88** by tandem Pd(0)-catalysed amidation/aldol condensation of appropriate aryl halides **85** with acetamides **86** [46].

Pd-catalysed amidation of halo aromatics substituted in the *o*-position by a carbonyl functional group or its equivalent **85** with primary or secondary amides **86** gave substituted quinolin-2(1*H*)-ones **87** (Scheme 28) [46]. Both aryl bromides and chlorides are effective in this reaction but aryl chlorides required longer reaction times than bromides (6 h versus 2 h). On the other hand, electron-rich aryl bromides afforded the product in moderate yield (47%). The reaction was not limited to *o*-halo aldehydes; enolizable methyl ketones, such as 2-bromoacetophenone, 2-bromobenzophenone, methyl 2-bromobenzoate, methyl 2-bromo-5-phenylbenzoate, 2-bromobenzonitrile also coupled with 2-phenylacetamide to give 4-substituted quinolin-2(1*H*)-one derivatives **88** in moderate to good yields (33–74%). Primary aryl and heterocyclic acetamides worked well with the exception of 2-pyridylacetamide that did not react in the reaction conditions presented in Scheme 28. With the secondary amide, *N*-methyl-2-phenylacetamide, the product was obtained in good yield (60%) however, neither the more sterically demanding *N*-isopropyl-2-phenylacetamide nor the *N*-arylamide *N*-phenyl-2-phenylacetamide was coupled with 2-bromobenzaldehyde using these conditions. Primary and secondary alkyl amides, such as propionamide and 2-cyclopropylacetamide, undergo coupling but failed the in situ cyclization; in addition, other attempts to cyclize the coupling products in several acidic or basic conditions failed. 2-Methoxyacetamide and *N*-methylpropionamide were coupled with 2-bromobenzaldehydes but also failed the cyclization in the coupling reaction conditions. However, they cyclized into the expected quinolin-2(1*H*)-ones, in low yields (51% and 32%, respectively), by the addition of NaO-*t*-Bu in *t*-BuOH.

**Scheme 29:**
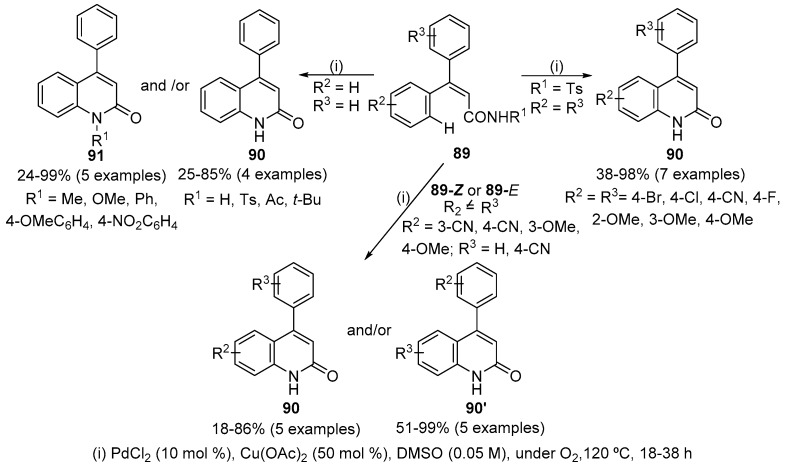
Synthesis of 4-arylquinolin-2(1*H*)-ones **90**, **90**′ and **91** by Pd-catalysed intramolecular amidation of 3,3-diarylacrylamides **89** [47,48].

Pd-catalysed intramolecular amidation of *N*-substituted-3,3-diarylacrylamides **89** was efficiently performed in the presence of a catalytic amount of PdCl_2_ and Cu(OAc)_2_ under O_2_ atmosphere, affording a range of diversely substituted 4-arylquinolin-2(1*H*)-ones **90** and **91** (Scheme 29) [47,48]. The cyclization of the 3,3-diarylacrylamides having tosyl, acetyl or a *t*-butyl group on the nitrogen atom of the amide moiety proceeded with concomitant loss of the protecting group, whereas the use of free amide or alkyl amides led to the product in poor yields. The benzene ring is also a suitable substituent on the nitrogen atom and, in this case, the reaction yield increases as the electron density of the benzene ring decreases, suggesting that the nucleophilicity of the nitrogen atom plays an important role in the process. This method is applicable to a wide range of symmetrical 3,3-diarylacrylamides (R^2^ = R^3^) possessing various functional groups on the benzene ring (Scheme 29). Unsymmetrical substrates (R^2^ ≠ R^3^) having a cyano group at the *p*-position of the benzene ring yielded only one regioisomer. In the case of the *m*-cyano substituted compounds the (*E*)-isomer was much more reactive than the (*Z*)-isomer (Scheme 29).

**Scheme 30:**
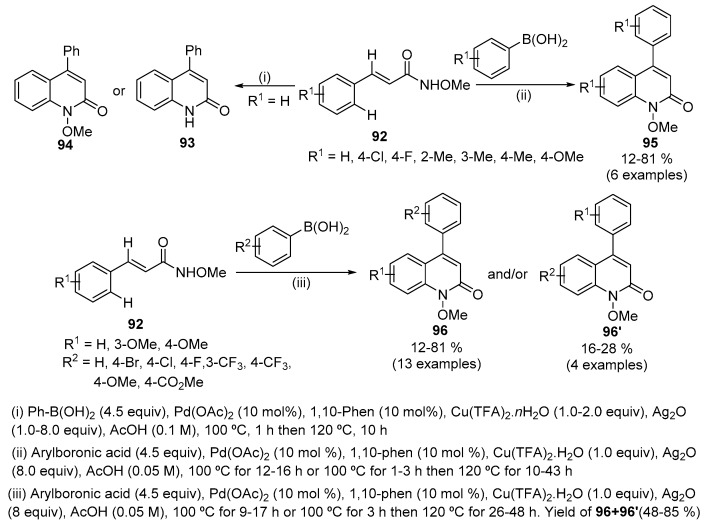
Synthesis of 4-arylquinolin-2(1*H*)-ones **93**, **94** and **95** via symmetrical 3,3-diarylacrylamides **92** and **96** and/or **96**′ via unsymmetrical 3,3-diarylacrylamides **92** [48,49].

A tandem process consisting of two mechanistically independent sequential Pd(II)-catalysed reactions, the oxidative Heck reaction of cinnamamides **92** with arylboron compounds (Ar′–“B”) followed by the intramolecular C–H amidation reaction, represented a facile and novel route to various substituted 4-arylquinolin-2(1*H*)-one derivatives (Scheme 30) [48,49]. The coupling of *N*-methoxycinnamamide **92** with phenylboronic acid using a catalytic combination of Pd(OAc)_2_/1,10-phenanthroline, Cu(TFA)_2_·nH_2_O (200 mol%) and Ag_2_O (100 mol%) in AcOH successfully delivered the expected quinolin-2(1*H*)-one **93**. In this case, demethoxylation unexpectedly occurred during the reaction, resulting in the formation of *N*-free quinolin-2(1*H*)-one **93** in fairly good yield (60%). The use of increased amounts of Ag_2_O (e.g., 300 mol% or more) completely inhibited demethoxylation and resulted in the formation of quinolin-2(1*H*)-one **94** in 52–81% yield (Scheme 30). The best result was obtained when 800 mol% of Ag_2_O was employed, affording **94** in high yields (81%). During the process, the use of a large excess of re-oxidants may inhibit the undesired N–O bond cleavage in which palladium and/or copper salt(s) are involved. This tandem process is also applicable to the synthesis of quinolin-2(1*H*)-ones via the formation of symmetrical 3,3-diarylacrylamides [48,49]. Reactions of cinnamamides with a methyl group or a halogen atom at the *p*-position of the benzene ring proceeded efficiently (59–76%), whereas that of cinnamamide with a methoxy group at the same position, resulted in the formation of the corresponding quinolin-2(1*H*)-one **95** (R^1^ = 4-OMe) in only 12% yield, along with the recovery of the starting material in 85% yield. For cinnamamides with a methyl group at the *m*-position of the benzene ring, C–H cyclization occurred at the less-hindered site; in contrast, the introduction of a methyl group at the *o*-position of the benzene ring did not result in the formation of the coupling or cyclized product (Scheme 30).

Reactions via the formation of unsymmetrical 3,3-diarylacrylamides were also investigated (Scheme 30) [48,49]. Although 4-methoxyphenylboronic acid was not suitable for this process, other arylboronic acids possessing a trifluoromethyl group, a methoxycarbonyl group or a halogen atom on the benzene ring (R^2^) successfully underwent reaction, affording different substituted 4-arylquinolin-2(1*H*)-ones **96** and/or **96**′. For example, cinnamamide **92** (R^1^ = H) reacted with the 3-trifluoromethylphenylboronic acid (R^2^ = 3-CF_3_) under optimized conditions to successfully afford quinolin-2(1*H*)-one **96** (R^1^ = H, R^2^ = 3-CF_3_) as the sole product in 67% yield. No regioisomer was identified, which suggests that the alkene isomerization of the Heck product formed in situ did not occur during the process. Likewise, the reaction of cinnamamide **92** (R^1^ = 3-OMe) with 4-chlorophenylboronic acid (R^2^ = 4-Cl) afforded only quinolin-2(1*H*)-one **96** (R^1^ = 3-OMe, R^2^ = 4-Cl) in 72% yield. However, with cinnamamide **92** (R^1^ = 4-OMe) sometimes a lower selectivity was observed, giving rise to a mixture of the corresponding quinolin-2(1*H*)-ones **96** and **96**′, indicating that the (*E*→Z)-isomerization could be occurring during the reaction course [49].

### 2.8. Intramolecular Hydroarylation of C-C Triple Bonds

**Scheme 31:**
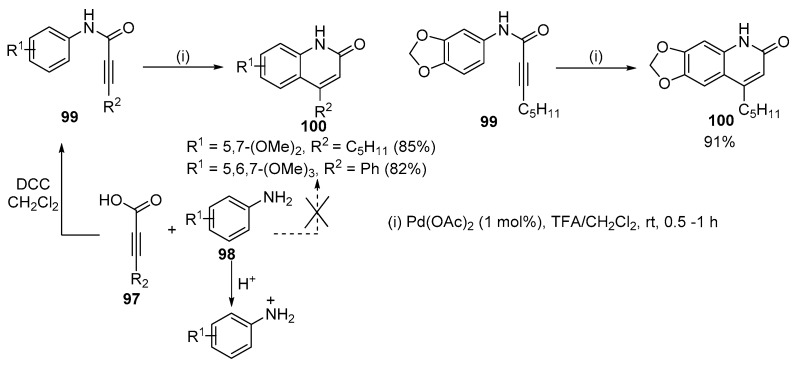
Synthesis of quinolin-2(1*H*)-ones **100** by Pd-catalysed intramolecular addition reactions of the corresponding alkynanilides **99** [50].

Various aryl alkynanilides **99**, prepared from the corresponding alkynoic acids **97** and anilines **98**, undergo fast intramolecular reaction, at room temperature, in the presence of a catalytic amount of Pd(OAc)_2_ in a mixed solvent containing TFA, affording quinolin-2(1*H*)-ones **100** in very good yields (82–91%), with more than 1000 turnover numbers (TON) to Pd (Scheme 31) [50]. The methodology tolerated a number of functional groups such as Br and CHO. On the basis of isotope experiments, a possible mechanism involving ethynyl chelation-assisted electrophilic metalation of aromatic C-H bonds by in situ generated cationic Pd(II) species was proposed. The involvement of vinyl-cationic species was also suggested [50]. On the other hand, it was demonstrated that the intermolecular reaction of alkynoic acids **97** and anilines **98** did not afford quinolin-2(1*H*)-ones **100**, presumably because the amino groups in anilines are converted in TFA to ammonium ions which act as electron-withdrawing groups and consequently deactivate the aromatic rings (Scheme 31) [50].

### 2.9. [3 + 3] Annulation

**Scheme 32:**
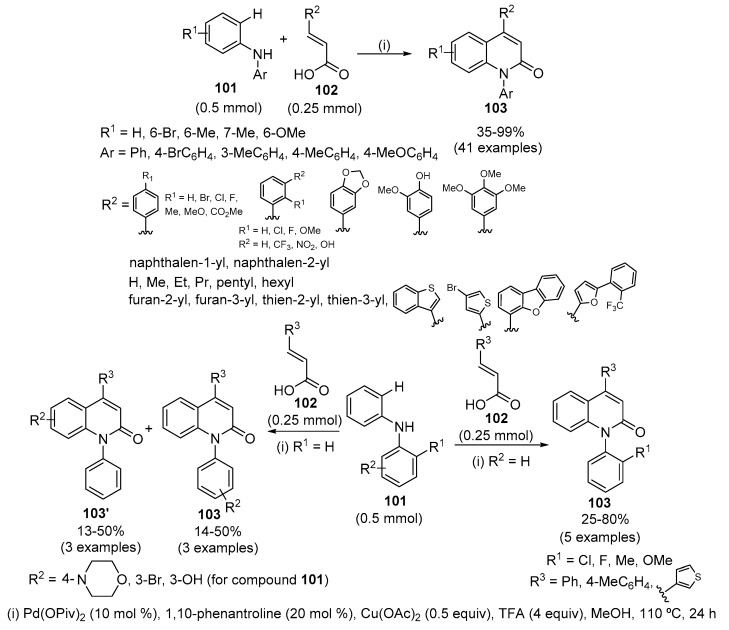
Synthesis of 4-substituted quinolin-2(1*H*)-ones **103** and **103**′ by Pd-catalysed [3 + 3] annulation between diarylamines **101** and α,β-unsaturated acids **102** [51].

The Pd-catalysed [3 + 3] annulation between diarylamines **101** and α,β-unsaturated acids **102** through C-H activation allowed direct access to 4-substituted-quinolin-2(1*H*)-ones **103** in high yield (Scheme 32) [51]. The use of TFA was crucial for suppressing facile decarboxylation of α,β-unsaturated acids. This method proved general and versatile for the synthesis of a variety of 4-substituted-quinolin-2(1*H*)-ones with aromatic, aliphatic and heterocyclic substituents at the 4-position. Although all of the substrates reacted successfully, electron-rich cinnamic acids gave better yields. Monoarylamines (ArNHR, R=H, Me, Et, *i*-Pr, Ts and Ac) either formed mixtures of inseparable compounds or gave low yields of the desired quinolones. Interestingly, all *o*-substituted unsymmetrical diarylamines gave single regioisomeric products. Based on preliminary mechanistic studies, involving competition experiments, intermediate studies and deuterium labelling, a reaction sequence was proposed, involving *o*-palladation, п-coordination, β-migratory insertion and β-hydride elimination [51].

### 2.10. Other Reactions

**Scheme 33:**
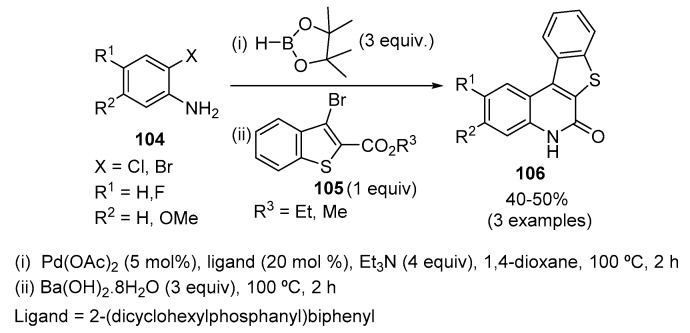
Synthesis of benzothieno[2,3-*c*]quinolin-6(5*H*)-ones **106** by one-pot three steps Pd-catalysed borylation, Suzuki coupling and amidation [52].

Benzothieno[2,3-*c*]quinolin-6(5*H*)-ones **106** were prepared by one-pot three steps Pd-catalysed borylation, Suzuki coupling and amidation (lactamization), starting from *o*-haloanilines **104** and alkyl 3-bromobenzo[*b*]tiophene-2-carboxylates **105** (Scheme 33) [52]. The amidation reaction probably occurs in the Suzuki coupling intermediate, through a nucleophilic attack of the nitrogen atom of the amine on the carbonyl of the ester group, with loss of methanol or ethanol, giving the corresponding tetracyclic quinolone. The use of an electron-rich sterically hindered ligand, such as 2-(dicyclohexylphosphanyl)biphenyl and Ba(OH)_2_·8H_2_O as base, is convenient for sterically hindered substrates. In addition, the borylation should be performed in the component bearing an *o*-electron-donating group with the other Suzuki coupling component having an *o*-electron-withdrawing group. This method allows the use of two bromo components conveniently substituted as starting materials. The in situ Pd-catalysed borylation avoids the preparation and isolation of boronic acids or esters and occurs with atom economy. The borylation occurred either using *o*-bromo or *o*-chloroanilines enhancing the scope of the reaction.

**Scheme 34:**
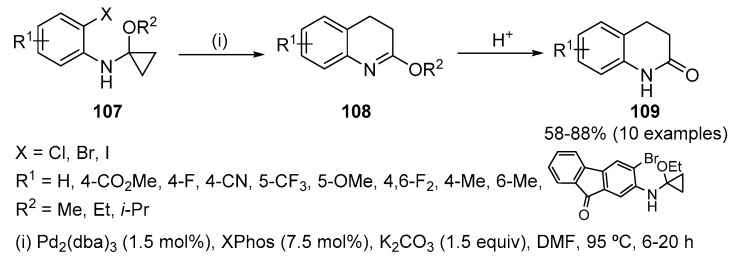
Synthesis of 3,4-dihydroquinolin-2(1*H*)-ones **109** via Pd-catalysed cyclopropane ring expansion [53].

*N*-(1′-Alkoxy)cyclopropyl-2-haloanilines **107** were converted to 3,4-dihydroquinolin-2(1*H*)-ones **109** via Pd-catalysed cyclopropane ring expansion (Scheme 34) [53]. Good yields of **108** were obtained using electron-rich biphenyl-based phosphine ligands; however, the more sterically demanding 2-dicyclohexylphosphino-2′,4′,6′-triisopropylbiphenyl (XPhos) provides the better yield. The use of K_2_CO_3_ as base led to the best result while KO*t*-Bu did not afford the desired compound. A lower reaction temperature resulted in a lower yield and DMF provided better yields than toluene or 1,4-dioxane. Bromo- and iodoaniline derivatives gave better yields in a shorter reaction time than chloroaniline. Hydrolysis of 2-alkoxy-3,4-dihydroquinoline **108** resulted in the formation of 3,4-dihydroquinolin-2(1*H*)-ones **109** in good yields. The reaction tolerates a variety of functional groups such as ester, nitrile, ether and ketone groups. The substrate bearing a nitro group did not give the desired compound.

A plausible mechanism for the formation of **108** starts with the oxidative addition of aryl halide **107** to Pd(0) followed by intramolecular ligand exchange to give a four-membered azapalladacycle. Further Pd rearrangement accompanied with cyclopropane ring opening gave the energetically favourable seven-membered azapalladacycle [53]. Finally, reductive elimination produces 2-methoxy-3,4-dihydroquinoline **108**, with concomitant regeneration of the reactive Pd(0) species. The hydrolysis of 2-methoxy-3,4-dihydroquinolines **108** furnishes the 3,4-dihydroquinolin-2(1*H*)-ones **109** [53].

**Scheme 35:**
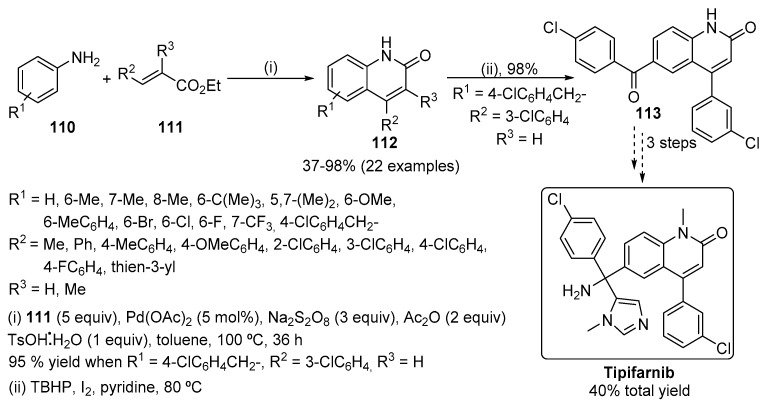
Synthesis of quinolin-2(1*H*)-ones **112** by a one-pot catalysed successive ammonolysis, C-H bond activation and cyclization reactions, using simple anilines **110** as substrates [54].

Liu and co-workers reported a concise and general strategy for the synthesis of quinolin-2(1*H*)-ones **112** by a one-pot catalysed cascade that includes successive ammonolysis, C-H bond activation and cyclization reactions, using simple anilines **110** as substrates (Scheme 35) [54]. Under the optimized reaction conditions, a wide range of quinolin-2(1*H*)-ones **112** was prepared in good to excellent yields. Anilines containing electron-donating, electron-withdrawing groups or halogens as substituents are effective in the reaction. For *m*-toluidine and 3-trifluoromethylaniline, C−H bond activation occurred solely at *p*-position to the methyl or trifluoromethyl group to provide the corresponding products in 96% and 53% yields, respectively. However, the reactions with anilines bearing trifluoromethyl, nitro or carboxyl groups at the *p*-position, did not give the desired quinolin-2(1*H*)-ones. When using cinnamates, the electronic character of the substituents on the aryl ring significantly influenced the efficiency of the cyclization. Cinnamates with electron-donating groups on the aryl moiety displayed higher reactivity than those with electron-withdrawing groups. No product was obtained when using ethyl (2*E*)-3-(2-trifluoromethylphenyl)propenoate but heterocyclic substituents containing propenoate and alkyl-substituted propenoates, such as ethyl crotonate and ethyl methacrylate, afforded the desired quinolin-2(1*H*)-ones **112** in low to moderate yields (37–68%). The utility of this method was demonstrated by a formal synthesis of Tipifarnib, an orally active inhibitor of farnesylprotein transferase with potent activity against neoplastic diseases, antineoplastic activity in solid tumours, such as breast cancer and in haematological malignancies found in leukaemia (Scheme 35).

**Scheme 36:**
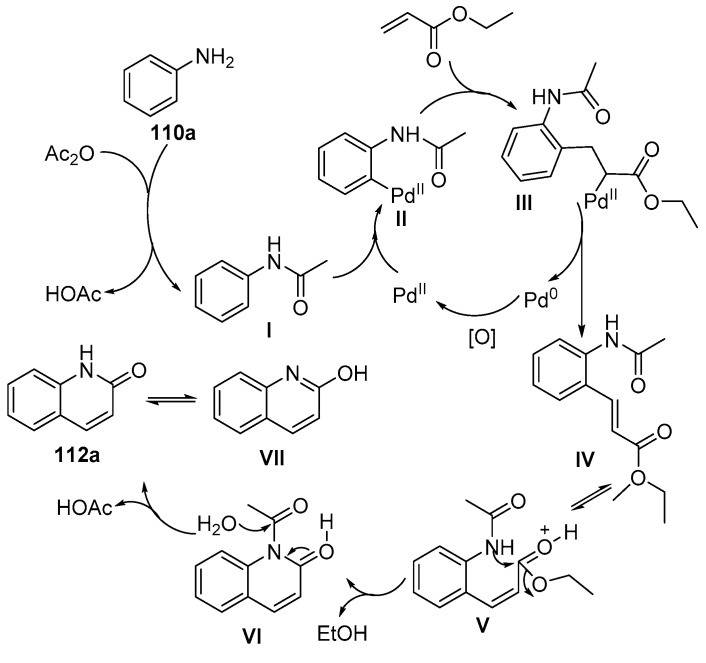
Plausible mechanism for the successive ammonolysis, C-H bond activation and cyclization of anilines **110** [54].

A plausible mechanism for this cascade process involves the formation of an aryl-Pd complex II via C−H activation at the C2-position of the substrate assisted by an in situ *N*-acetylation. Next, Heck-type coupling of II with acrylate through Pd complex III forms intermediate IV and releases the Pd(0) species. Then oxidation of Pd(0) by Na_2_S_2_O_8_ to Pd(II) completes the metal catalytic cycle. Under acidic conditions, the intermediate IV could be transferred to intermediate V and followed the ammonolysis of the ester with the amide to give the intermediate VI, which after hydrolysis gave the expected quinolin-2(1*H*)-one (Scheme 36) [54].

**Scheme 37:**
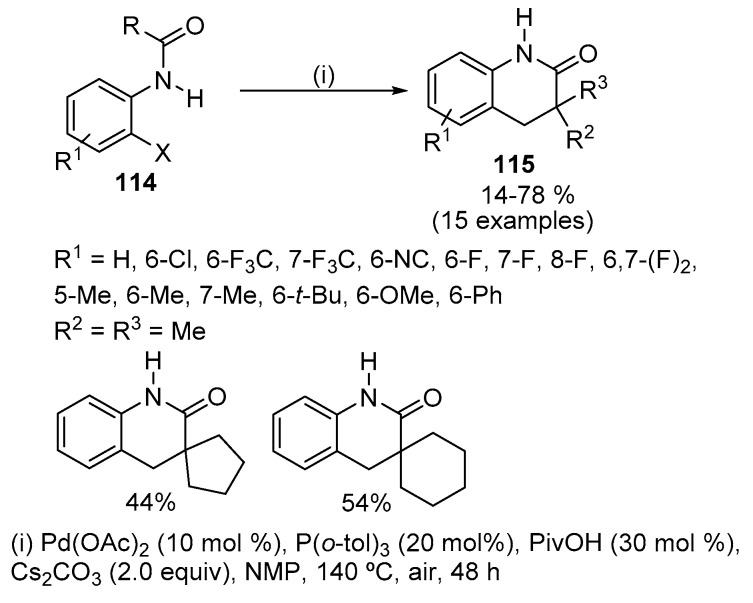
Synthesis of 3,3-disubstituted 3,4-dihydroquinolin-2(1*H*)-ones **115** by Pd-catalysed, oxidative-addition-initiated activation and arylation of inert C(sp^3^)-H bonds [55].

3,3-Disubstituted-3,4-dihydroquinolin-2(1*H*)-ones **115** were synthesized from easily available *o*-halogenated acetylide derivatives **114** by the Pd-catalysed oxidative-addition-initiated activation and arylation of inert C(sp^3^)-H bonds (Scheme 37) [55]. Pd(OAc)_2_ and P(*o*-tol)_3_ were used as the catalyst and ligand, respectively, to improve the efficiency of the reaction. Polar solvents, such as *N*-methyl-2-pyrrolidone (NMP) gave the best results. The use of PivOH (Piv = pivaloyl) as additive is not essential; however the product was obtained in a slightly better yield in the presence of this additive. Regarding the reactivity of the C-X bonds, chloro-substituted substrates showed a low reactivity and iodo-substituted compounds also gave a lower yield than bromo-substituted ones. When there are substituents at 3- or 6-position, the cyclized products were obtained in low yields, which indicate that steric hindrance plays a key role in influencing the reaction efficiency. Furthermore, some sensitive functional groups, such as nitro, ester and acyl groups were not tolerated. Nonetheless, by changing the acyl group from the pivaloyl group to a 1-methylcyclopentanecarbonyl or a 1-methylcyclohexanecarbonyl group, it is possible to synthesize polycyclic compounds with spiro-centres. However, in the presence of a C(sp^2^)-H bond located at a suitable position for intramolecular C-H activation, the desired product of the inert C(sp^3^)-H activation could not be observed. A further advantage of this reaction is that it could be performed in air. Mechanistic studies showed that a relatively rare seven-membered palladacycle is a key intermediate of the catalytic cycle [55].

**Scheme 38:**
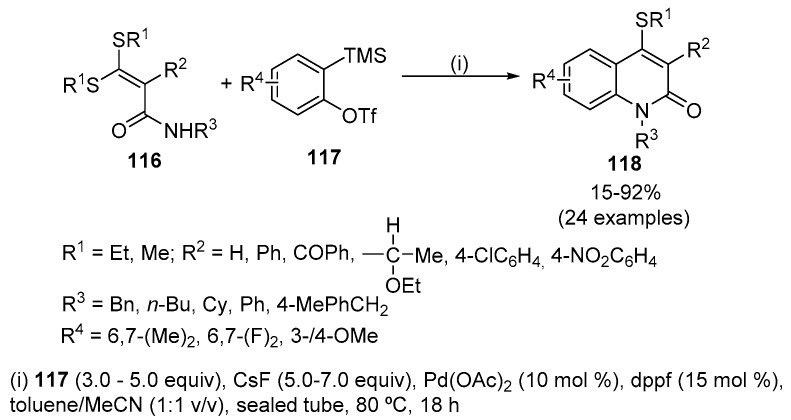
Synthesis of quinolin-2(1*H*)-ones **118** by Pd-catalysed C-S activation/aryne insertion/coupling sequence [56].

A wide range of α-carbamoyl ketene dithioacetals **116** readily reacted with arynes **117** to selectively afford functionalized quinolin-2(1*H*)-ones **118** in high yields under neutral reaction conditions by a C-S activation/aryne insertion/intramolecular coupling sequence catalysed by Pd(OAc)_2_ (Scheme 38) [56]. The use of dppf, as a ligand, dramatically improved the aryne annulation and both palladium and the ligand play a key role in the insertion of benzyne into a C-S bond. Other phosphine-containing ligands, such as PCy_3_ (Cy = cyclohexyl), PPh_3_ and Xantphos, were found to be less efficient than dppf for this annulation. Additionally, either decreasing the amount of the catalyst or lowering the reaction temperature resulted in unsatisfactory yields of **118a** (R^1^ = Me, R^2^ = 4-NO_2_C_6_H_4_, R^3^ = Bn, R^4^ = H). The reaction scope is quite general, however for dithioacetals which do not have a substituent at the α-position (R^2^ = H) the reaction was more complex and the quinolin-2(1*H*)-one **118** (R^2^ = H) was obtained in a low yield (27%), along with quinolin-2(1*H*)-one **118** (R^2^ = Ph) that results from the first α-phenylation of the dithioacetal (R^2^ = H) with benzyne by a Pd-catalysed α-C-H activation and subsequent annulation with the benzyne. Finally, the reaction was found to be sensitive to substrates **116** bearing a free NH_2_; in this case, the obtained product was only detected by NMR and HRMS-ESI studies of the reaction mixture after 18 h of reaction time. When the unsymmetrical aryne 4-methoxy-2-(trimethylsilyl)-phenyl trifluoromethanesulfonate was employed, a mixture of two isomeric quinolin-2(1*H*)-ones was obtained [56].

**Scheme 39:**
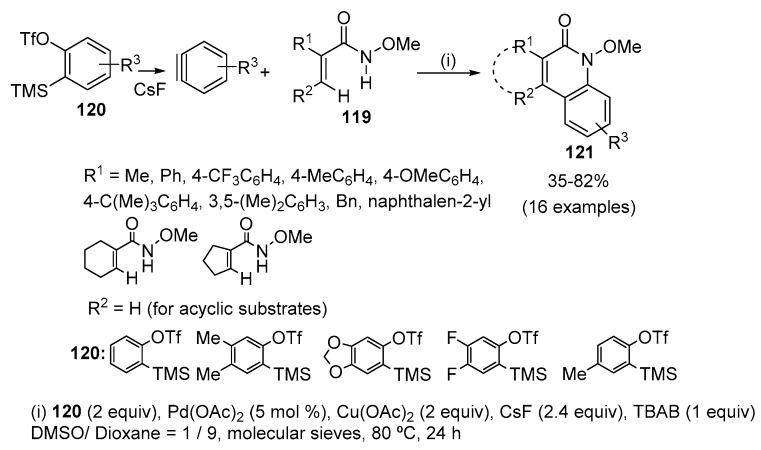
Synthesis of quinolones **121** by Pd-oxidative annulation between acrylamides **119** and arynes **120** [57].

The Pd-catalysed oxidative annulation of electron-deficient acrylamides **119** with benzyne precursors **120**, in the presence of Pd(OAc)_2_, Cu(OAc)_2_ and CsF, in a mixture of dioxane and DMSO as solvent at 80 °C, allowed the formation of quinolin-2(1*H*)-one **121** in good yields (Scheme 39) [57]. Pd(PPh_3_)_4_ can also be employed but the product is generated in lower yield. Tetrabutylammonium bromide (TBAB) was found to be an important additive leading to highly improved yields. Acrylamides **119** bearing alkyl or aromatic substituents at α-position are good substrates in the reaction with benzyne. Cyclic substrates also react efficiently to give the expected fused-quinolin-2(1*H*)-ones **121** in moderate to good yields (55–74%) but cinnamide and crotonyl amide did not react. Substituted arynes bearing both electron-donating and electron-withdrawing substituents reacted with acrylamide **119**: R^1^ = Ph, R^2^ = H, generating the corresponding quinolin-2(1*H*)-ones **121** in low to moderate yield (35–61%); with the asymmetric aryne, a 1:1 mixture of regioisomers was obtained. The methoxy group on the nitrogen atom is a very important protecting group. It was easily removed by NaH to give free quinolin-2(1*H*)-one **121** in 93% yield. The oxidative annulation of an *N*-methylacrylamide with benzyne gave the corresponding quinolin-2(1*H*)-one in only 25% yield under the same reaction conditions.

**Scheme 40:**
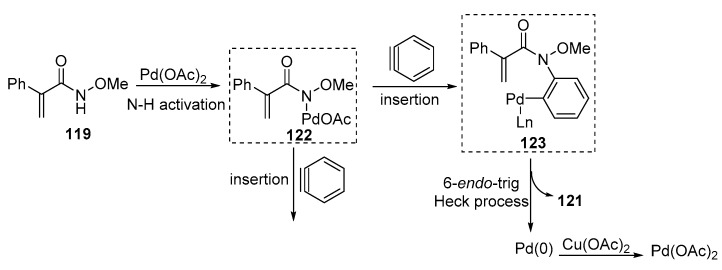
Plausible mechanism for the formation of quinolones **121** [57].

Although two possible reaction pathways for the oxidative annulation are possible [57], the most probable one involves the N-H activation of acrylamide **119** followed by aminopalladation of intermediate **122** to benzyne originating the palladium intermediate **123**, which went through a Heck-type reaction, insertion to the electron-deficient double bond and subsequent β-hydrogen elimination, producing the expected quinolin-2(1*H*)-one **121** (Scheme 40).

**Scheme 41:**
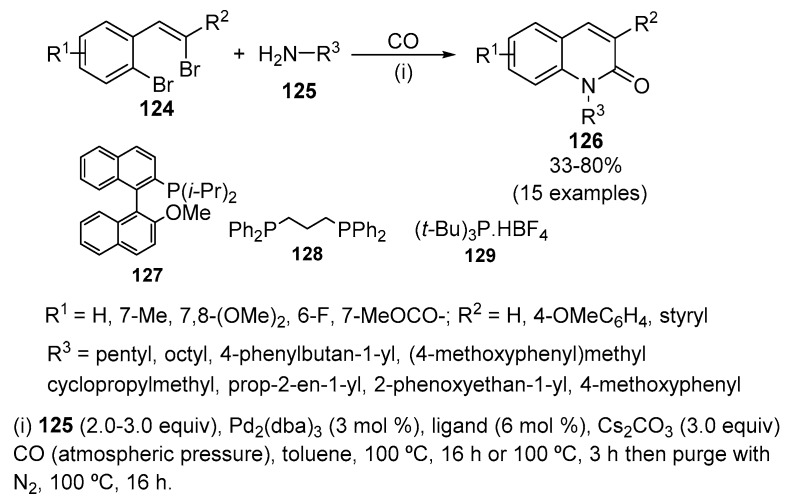
Synthesis of quinolin-2(1*H*)-ones **126** by Pd-catalysed aminocarbonylation/ intramolecular amidation cascade sequence starting from 2-(2-haloalkenyl)aryl halides **124** [58].

Pd-catalysed intermolecular aminocarbonylation/intramolecular amidation cascade sequences were employed to efficiently and selectively convert a range of 2-(2-haloalkenyl)aryl halides **124** to the corresponding quinolin-2(1*H*)-ones **126** (Scheme 41) [58]. In the presence of Pd_2_(dba)_3_ and Cs_2_CO_3_ a variety of ligands, such as P(*i*-Pr)_2_ **127**, diphosphine dppp **128** and P(*t*-Bu)_3_ **129**, efficiently delivered quinolin-2(1*H*)-ones **126** in reasonable yields. Alkyl amines and *p*-methoxybenzylamine were suitable *N*-nucleophiles for this reaction. With allylamine, the reaction was performed at 50 °C for 2 h before heating at 100 °C. With *O*-phenylethanolamine CO atmosphere had to be removed after 3 h of reaction in the standard conditions. The use of *p*-anisidine required Xantphos as ligand and afforded the *N*-arylquinolone in only 33% yield.

A better yield was obtained following a discrete two-step process involving isolation of the intermediate *N*-arylamide and then re-subjection of the amide to ring closure conditions. Xantphos was employed as the ligand for both steps and allowed the isolation of the corresponding quinolin-2(1*H*)-one in 65% yield for the two steps. A range of electron-donating and electron-withdrawing substituents are tolerated in the aryl ring of the dihalide substrate. This method also allows the synthesis of quinolin-2(1*H*)-ones having aryl or styryl substituents at C-3 position.

Isoquinoline derivatives can be obtained using the same conditions but delaying the introduction of the CO atmosphere [58]. A limitation of this approach is the requirement to employ a sterically demanding *N*-nucleophile, as the use of less hindered coupling partners results in competitive indole formation.

## 3. Synthesis of Quinolin-4(1*H*)-ones

### 3.1. Sonogashira Reaction

**Scheme 42:**
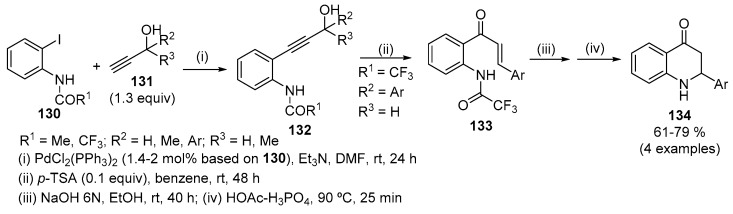
Synthesis of 2,3-dihydroquinolin-4(1*H*)-ones **134** from 2-substituted anilides **132** obtained through Pd-catalysed reaction of 2-iodoanilides **130** with acetylenic carbinols **131** [59,60].

2-Iodoanilides **130** reacted with terminal propargyl alcohols **131** under Pd-catalysed conditions to yield the corresponding 2-substituted anilides **132** (Scheme 42) [59,60]. PdCl_2_(PPh_3_)_2_ was found to be the best catalyst. Propargyl alcohols **131** in the presence of Et_3_N appear to be capable to reduce Pd(II) to Pd(0) before the oxidative addition of Pd(0) to the aryl halide could occur. Only in one case (R^1^ = CF_3_; R^2^ = R^3^ = H) the corresponding indole, obtained by spontaneously cyclization of 2-substituted anilides **132**, was isolated. By acid-catalysed Meyer-Schuster rearrangement, 2-substituted anilides **132** were converted to *N*-substituted 2′-aminochalcones **133** which upon alkaline hydrolysis, deprotection and cyclization, under acidic conditions, afforded 2-aryl-2,3-dihydroquinolin-4(1*H*)-ones **134** (Scheme 42).

**Scheme 43:**
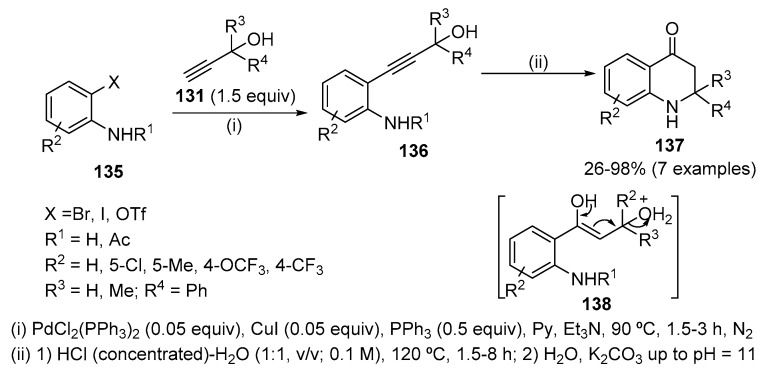
Synthesis of 2,3-dihydroquinolin-4(1*H*)-ones **137** via Sonogashira coupling of 2-(pseudo)haloanilines **135** with propargyl alcohols **131** followed by acid-catalysed tandem Rupe rearrangement-Donnelly-Farrell ring closure of anilines **136** [61].

The Sonogashira coupling of 2-(pseudo)halogenated anilines **135** with propargyl alcohols **131** followed by a Brønsted acid-catalysed cyclization of the resulting 2-(3-hydroxypropynyl)anilines **136** afforded 2,3-dihydroquinolin-4(1*H*)-ones **137** (Scheme 43) [61]. Anilines **136** were converted into quinolin-4(1*H*)-ones by acid-catalysed tandem Rupe rearrangement-Donnelly-Farrell ring-closure reaction upon treatment with concentrated HCl-H_2_O (1:1, *v*/*v*), at 120 °C, followed by basic workup. The Rupe rearrangement of **136**, in the case of free aniline, involves a regioselective hydration-dehydration rearrangement of the alkyne moiety, probably via aldol **138**, to give the corresponding α,β-unsaturated ketone, which by acid catalysed 6-*endo*-trig Michael-type ring closure is converted into dihydroquinolin-4(1*H*)-ones **137** (Scheme 43). The cyclization of the *N*-acetyl derivatives occurs via Rupe rearrangement followed by acetamide hydrolysis, in situ, immediately prior to cyclization [61]. This reaction allows the introduction of different groups at C-2 of the quinolin-4(1*H*)-one skeleton (e.g., R^3^ = H, Me; R^4^ = Ph) thus providing access to a wide variety of 2-substituted 2,3-dihydroquinolin-4(1*H*)-ones **137**.

**Scheme 44:**
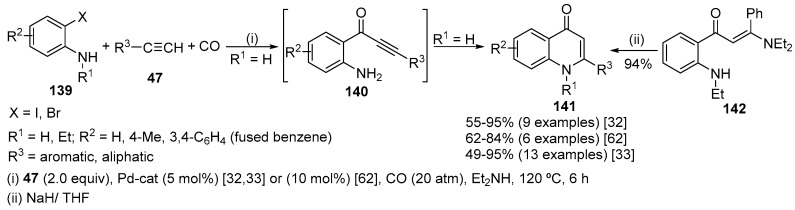
Synthesis of 2-substituted quinolin-4(1*H*)-ones **141** by Pd-catalysed carbonylative coupling of 2-haloanilines **139** with terminal acetylenes **47** [32,33,62].

The Pd-catalysed carbonylative coupling of 2-haloanilines **139** with terminal arylacetylenes **47**, initially reported by Torii and Kalinin [32,33,62], appears to be the most versatile method for the synthesis of 2-substituted quinolin-4(1*H*)-ones **141**. The desired compounds were obtained in good yields using either PdCl_2_(PPh_3_)_2_ or PdCl_2_(dppf) and an excess of Et_2_NH which acts as solvent and base and plays a key role in the cyclization step (Scheme 44). 2-Iodoanilines are preferred to 2-bromoanilines and react both as a free base and in the hydrochloride form although the latter in lower yield [62]. The reaction of arylacetylenes generally gave better yields than aliphatic acetylenes, which may be due to the acidity of the acetylene proton. Functional groups, such as thiophenyl, acetal, tetrahydropyran (THP), ester, keto and ether, tolerate the reaction conditions. When the nitrogen of the anilines has a substituent, decrease of the nucleophilicity retards the cyclization. In fact, reaction with alkylated aniline, under the same reaction conditions, led to the corresponding enamine **142** as main product (52%) and only 20% of quinolin-4(1*H*)-one **141** was obtained, although subsequent treatment of the enamine with sodium hydride in THF led to quinolin-4(1*H*)-one **141** quantitatively [32,33]. Decrease of CO pressure or temperature drops the reaction yield.

**Scheme 45:**
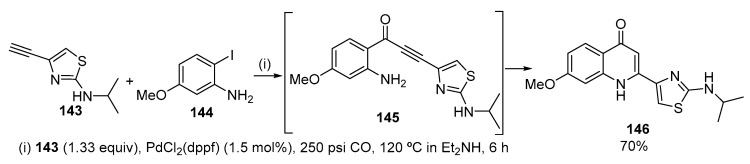
Synthesis of the quinolin-4(1*H*)-one substructure **146** by Pd-catalysed carbonylative Sonogashira coupling of 2-iodo-5-methoxyaniline **144** with thiazolylacetylene **143** [63].

Pd-catalysed carbonylative Sonogashira coupling of 2-iodo-5-methoxyaniline **144** with thiazolylacetylene **143** afforded quinolin-4(1*H*)-one **146**, a key substructure of the hepatitis C virus NS3 protease inhibitor BILN2061 (Scheme 45) [63]. This approach may also provide a practical and general access to polysubstituted quinolones related to structure **146**.

**Scheme 46:**
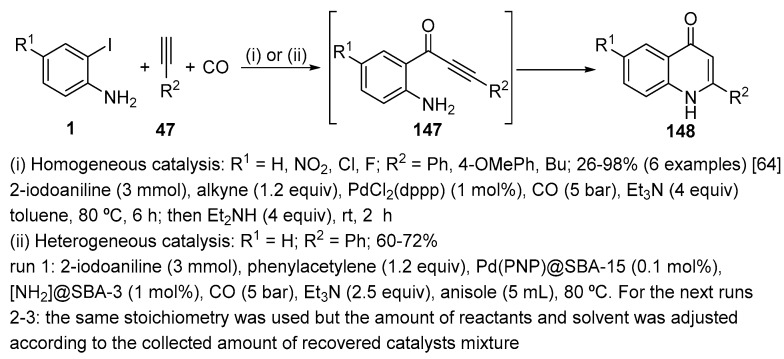
Synthesis of 2-substituted quinolin-4(1*H*)-ones **148** by a carbonylative Sonogashira coupling reaction under homogeneous and heterogeneous Pd-catalysis [24,64,65,66].

Pd-catalysed carbonylative Sonogashira coupling between 2-iodoanilines **1**, alkynes **47** and CO (5 bar), using Et_3_N as the base in the presence of PdCl_2_(dppp) [dppp = 1,3-*bis*(diphenylphosphino)propane] as catalyst selectively afforded intermediate **147**. In a second step, an organocatalyzed cyclization occurs after the addition of Et_2_NH to give the expected quinolin-4(1*H*)-ones **148** in high selectivity and yields [Scheme 46, (i)] [64]. Although successful, this one-pot two-step multi-catalysis method suffers from the need of homogenous catalysts, which are tedious to remove and could result in high Pd and ligand contamination of the final products that is not acceptable when dealing with animal and human health. The use of heterogeneous catalysts associating the [Pd(PNP)]@SBA-15 catalyst to a grafted amine catalyst as [NH_2_]@SBA-3 in a one-pot tandem [Pd/amine] mode allowed, for example, the selective synthesis of 2-phenylquinolin-4(1*H*)-one in a suitable 61% isolated yield [Scheme 46, (ii)] [65]. This approach resulted in a strong decrease of Pd-contamination in the final products as only 3 to 5 ppm of Pd was found in the crude quinolin-4(1*H*)-ones, while 40 ppm was measured when using homogeneous catalytic system. The overall reaction time was also reduced from 7 to 3 days (in the same reaction conditions). Recycling of the {[Pd(PNP)]@SBA-15/[NH_2_]@SBA-3} catalyst mixture was successful for 3 runs [66].

**Scheme 47:**
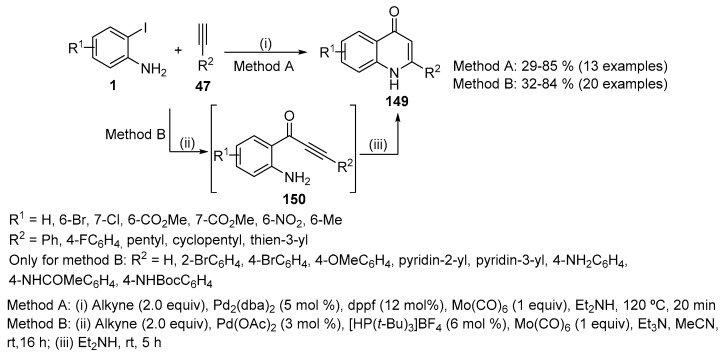
Synthesis of quinolin-4(1*H*)-ones **149** via carbonylative Sonogashira cross-coupling reaction using molybdenum hexacarbonyl as a CO source [67].

Two different protocols were developed for the Sonogashira coupling of 2-iodoanilines **1** with alkynes **47** and cyclization toward functionalized quinolin-4(1*H*)-ones **149** (Scheme 47) [67]. In the first protocol (Method A), quinolin-4(1*H*)-ones **149** were obtained after 20 min of microwave (MW) heating at 120 °C using Mo(CO)_6_, Pd_2_(dba)_3_ with an excess dppf, as ligand and Cs_2_CO_3_ in diethylamine. The reaction scope is quite general, however, when using 2-iodo-4-nitroaniline, the corresponding product was obtained in low yield, due to the thermally induced nitro group reduction by Mo(CO)_6_. To overcome this limitation, a second protocol (Method B) was developed using milder conditions, extending the scope of the reaction to reduction-prone or other sensitive groups, such as nitro and bromo substituents. The coupling reaction was performed at room temperature using a different catalytic system, Mo(CO)_6_, Pd(OAc)_2_, [HP(*t*-Bu)_3_]BF_4_ followed by the addition of diethylamine to promote the cyclization of the arylalkynone intermediate **150**. The formation of quinolin-2(1*H*)-ones was never detected using these two methods, on contrary to the previous observations of Chen and co-workers [35], who reported a Mo(CO)_6_ protocol for the formation quinolin-2(1*H*)-ones starting from **1** and **47**.

### 3.2. Buchwald-Hartwig Reaction

**Scheme 48:**
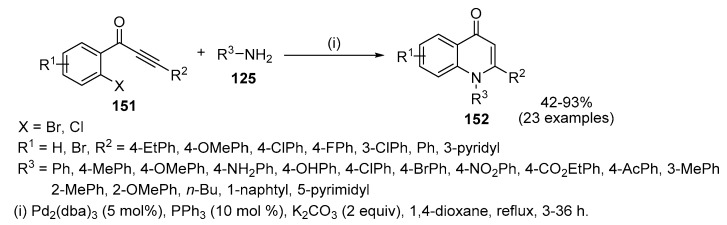
Synthesis of quinolin-4(1*H*)-ones **152** by a Pd-catalysed tandem amination reaction [68].

An efficient Pd-catalysed tandem amination approach, starting from easily accessible 2-haloaryl acetylenic ketones **151** and primary amines **125**, involving a sequential double C-N bond formation, furnished functionalized quinolin-4(1*H*)-ones **152** in good to excellent yields in one step (Scheme 48) [68]. The reaction of **151** (X = Br, R^1^ =H) with aniline in the presence of Pd(PPh_3_)_4_ in 1,4-dioxane, using K_2_CO_3_ as base, gave the corresponding quinolin-4(1*H*)-one **152** in 71% yield. A similar result was obtained using the PdCl_2_(dppf)-CH_2_Cl_2_ complex as catalyst. Improvements were made using Pd_2_(dba)_3_-CHCl_3_ as catalyst combined with Xantphos or dppp, however PPh_3_ proved to be the best ligand affording quinolin-4(1*H*)-one **152** in 84% yield. A range of commercially available aryl amines can be employed to give the corresponding products in moderate to good yields (61–93%). Even those with active amino or hydroxy groups remain intact under the reaction conditions. However, with aliphatic amines such as butylamine the product was obtained in moderate yield (42%). This reaction is also compatible with different types of ynones substituted with aryl and pyridyl groups.

**Scheme 49:**
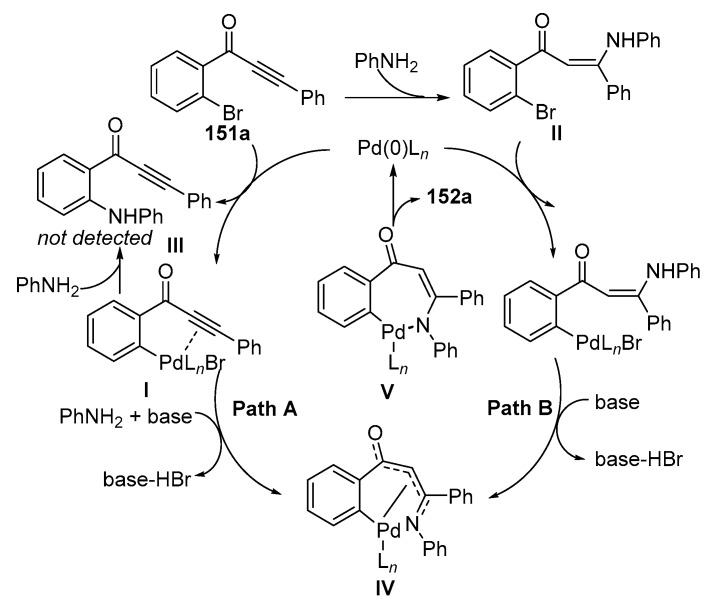
Plausible mechanism for the synthesis of **152** from **151** [68].

Two pathways were proposed for this reaction (Scheme 49), which may involve either oxidative addition of Pd(0) to the C-Br bond in **151** (intermediate I in Path A) or conjugate addition of aniline to **151** (intermediate II in Path B) in the first step. The formed intermediate I presumably leads to III through Buchwald-Hartwig amination or activation of a C≡C bond in I through coordination to the Pd and attack by aniline to form intermediate IV. Both pathways will go through intermediates IV and V, followed by reductive elimination of Pd(0) to give the desired quinolin-4(1*H*)-one **152**. Based on some experiments conducted by Xu and Zhao [68], path A could be the major pathway to afford the target quinolone.

**Scheme 50:**
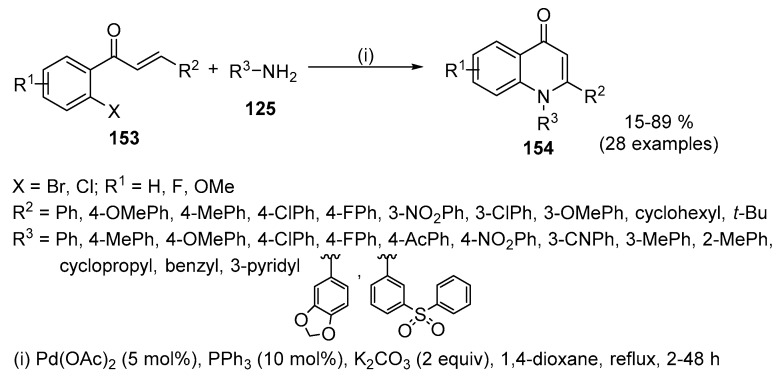
Synthesis of 1,2-disubstituted-quinolin-4(1*H*)-ones **154** through a Buchwald-Hartwig coupling and catalytic dehydrogenation sequence [69].

An efficient Pd-catalysed tandem amination protocol for the synthesis of 1,2-disubstituted quinolin-4(1*H*)-ones **154** was developed starting from easily accessible chalcones **153** and primary amines **125**, in which the Pd-catalyst [Pd(OAc)_2_] plays a dual role, namely, in the Buchwald–Hartwig coupling and catalytic dehydrogenation (Scheme 50) [69]. Pd_2_(dba)_3_ that was a good catalyst in the Pd-catalysed tandem amination of 2-haloaryl acetylenic ketones **151** and primary amines **125** [68] was less effective than Pd(OAc)_2_ in this transformation (55% and 74%, respectively). The procedure using PPh_3_ as a ligand in refluxing anhydrous 1,4-dioxane and K_2_CO_3_ as base was efficient with aromatic, heteroaromatic and aliphatic amines. Arylamines containing electron-donating groups gave higher yields (79–87%) than those with electron-withdrawing groups (54–78%) and aliphatic amines gave the expected quinolin-4(1*H*)-ones **154** in slightly lower yields (45–52%). Different functional groups (R^1^ and R^2^) of chalcones **153** were all compatible with the reaction conditions. Due to the low oxidative addition reactivity of the C–Cl bond, 2-chloro-substituted chalcones gave quinolin-4(1*H*)-ones **154** in lower yield than the corresponding bromo-derivatives, even upon raised temperature.

### 3.3. Stille Reaction

**Scheme 51:**
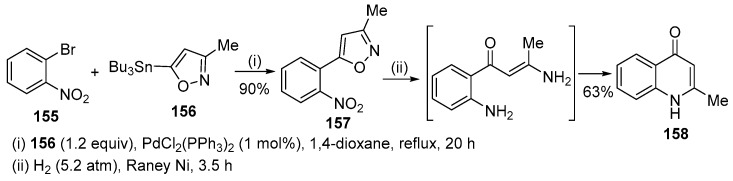
Synthesis of 2-methylquinolin-4(1*H*)-one **158** via Stille reaction followed by catalytic hydrogenation [70].

Pd-catalysed cross-coupling reaction of 5-(tributylstannyl)-3-methylisoxazole **156** with 2-bromonitrobenzene **155** followed by the catalytic hydrogenation of **157** over Raney nickel afforded 2-methylquinolin-4(1*H*)-one **158** in 57% overall yield (Scheme 51) [70].

**Scheme 52:**
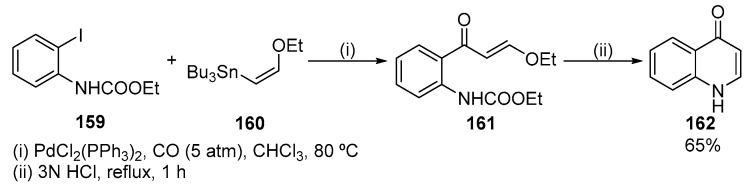
Synthesis of quinolin-4(1*H*)-one **162** via Stille reaction followed by cyclization [71].

The Pd-catalysed carbonylative coupling of ethyl 2-iodophenylcarbanylate **159** with (*Z*)-tributyl(2-ethoxyvinyl)stannane **160**, under CO atmosphere, afforded ethyl (*E*)-2-(3-ethoxy-1-oxoprop-2-en-1-yl)phenyl carbanylate **161**. Then compound **161** underwent cyclization under acidic conditions to give quinolin-4(1*H*)-one **162** (Scheme 52) [71].

### 3.4. Cyclocarbonylation Reaction

**Scheme 53:**
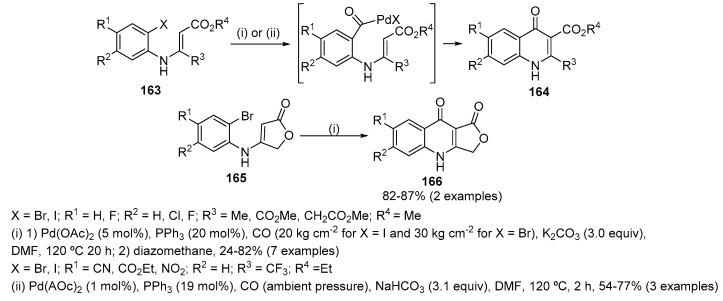
Synthesis of quinolin-4(1*H*)-ones **164** and **166** via Pd-catalysed carbonylative cyclization of 2-haloenamines **163** and **165** [72,73,74].

Carbonylation of 3-substituted 3-(2-haloarylamino)prop-2-enoates **163** in the presence of Pd-catalyst under CO atmosphere, at 120 °C, resulted in heterocyclization to form a variety of 2-substituted quinolin-4(1*H*)-one-3-carboxylates **164** (Scheme 53) [72]. Cyclocarbonylation of iodoenamine **163** (X = I) was effective at 20 kg·cm^−2^, whereas 30 kg·cm^−2^ were required for the bromoenamine **163** (X = Br). After treatment with diazomethane, to avoid the presence of quinolin-4(1*H*)-one-3-carboxylic acid, quinolin-4(1*H*)-ones **164** were obtained in moderate to good yields (55-82%) although derivatives involving 2-methoxycarbonyl group or 6,7-difluoride gave lower yields (24% and 37%, respectively) [Scheme 53, (i)]. Carbonylative heterocyclization of other functionalized enamines **165** resulted in the successful formation of the desired products **166** in 82% and 87% yield; furthermore, the hydrolysis of lactone **166** may provide other intriguing quinolin-4(1*H*)-one carboxylic acids [72]. This method is of practical importance because of the wide choice of the group R^3^ in addition to the fact that the benzene ring may carry a diverse number of substituents. Under similar conditions but using CO at atmospheric pressure, Stanforth and co-workers reported the synthesis of three 2-trifluoromethylated quinolin-4(1*H*)-ones in 54–77% yield [Scheme 53, (ii)] [73,74].

**Scheme 54:**
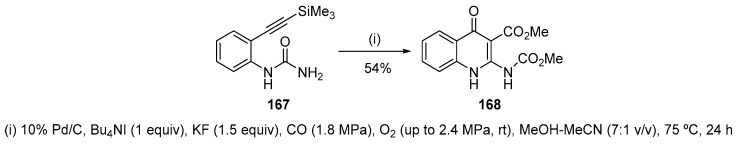
Synthesis of quinolin-4(1*H*)-one **168** via Pd-catalysed oxidative carbonylation of 2-ethynylaniline **167** [75,76].

Pd-catalysed cyclization-alkoxycarbonylation of 1-[(2-trimethylsilyl-ethynyl)phenyl]urea **167** using Pd/C-Bu_4_NI as catalyst in the presence of KF, for the in situ deprotection, gave quinolin-4(1*H*)-one **168** (Scheme 54) [75,76]. Formation of the compound **168** is quite intriguing and it was described as arising from some sort of rearrangement, probably via intermediate formation of a benzoxazine derivative. A reasonable mechanistic hypothesis was proposed by the authors but requires further investigation [45,75,76]. The nature of the substituent was found to be crucial for the product formation. In the case of an unsubstituted urea **167** quinolin-4(1*H*)-one **168** was the only isolated reaction product; but when it is substituted, no quinolin-4(1*H*)-one was obtained [75].

**Scheme 55:**
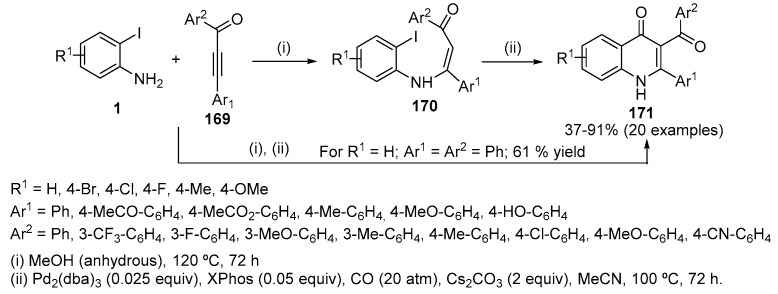
Two-step and sequential syntheses of 3-aroylquinolin-4(1*H*)-ones **171** by Pd-catalysed carbonylative cyclization of *N*-(2-iodoaryl)enaminones **170** [77].

Pd-catalysed carbonylative cyclization of readily available *N*-(2-iodoaryl)enaminones **170** afforded 2-substituted-3-aroylquinolin-4(1*H*)-ones **171** in very satisfactory yields (37–91%) (Scheme 55) [77]. Pd_2_(dba)_3_ was found to be the best catalyst but the use of a bulky monodentate ligand such as 2-dicyclohexylphosphino-2’,6’-dimethoxy-1,1′-biphenyl (SPhos) or XPhos is required as well as 20 atm of CO. Good to high yields are usually obtained with enaminones bearing both electron-donating and electron-withdrawing as well as neutral aromatic rings. A moderate yield of the desired 3-aroylquinolin-4(1*H*)-one derivative **171** is obtained with an *N*-(2-iodoaryl)-β-enaminone containing a bromine substituent in the aniline moiety. The synthesis of 2-substituted-3-aroylquinolin-4(1*H*)-ones **171** is also possible without isolation of the enaminone intermediate by adding Pd_2_(dba)_3_, XPhos, Cs_2_CO_3_, MeCN and CO (20 atm) to the crude mixture derived from the reaction of 2-iodoanilines **1** with α,β-ynones **169** after evaporation of the volatile materials. Under these conditions **171a** (R^1^ = H, Ar^1^ = Ar^2^ = Ph) was isolated in 61% overall yield.

**Scheme 56:**
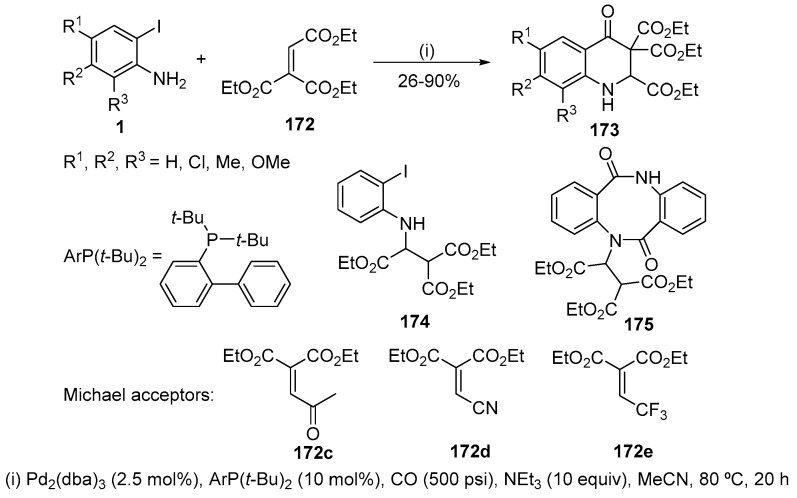
Synthesis of 2,3-dihydroquinolin-4(1*H*)-ones **173** by Pd-catalysed intermolecular cyclocarbonylation of 2-iodoanilines **1** and diethyl ethoxycarbonyl butendienoate **172** [78].

Highly functionalized 2,3-dihydroquinolin-4(1*H*)-ones **173** were obtained in one step, with moderate to good yields, by Pd-catalysed intermolecular cyclocarbonylation of 2-iodoanilines **1** with diethyl ethoxycarbonylbutendienoate **172** (Scheme 56) [78]. The method involves a Michael addition and subsequent carbonylation reactions using the catalytic system of Pd_2_(dba)_3_/2-(di-*t*-butylphosphino)biphenyl in MeCN at 80 °C under 500 psi of CO. Solvents like CH_2_Cl_2_ or THF promote the formation of the Michael addition product **174**, in more than 80% yield, being the unique reaction product since no carbonylation occurs. In general, better yields were obtained with electron-donating phosphines, such as trialkylphosphines and dialkylarylphosphines; however, 2-(di*-t*-butylphosphino)biphenyl was the better ligand. Xantphos behaved differently promoting the formation of **175** much more selectively than when using any other ligands. The reaction is sensitive to the electronic nature of the substituents at the *p*-position relatively to the iodide group. Highly electron-donating or electron-withdrawing groups, such as methoxy and chlorine, afforded **173** in lower yields (39% and 26%, respectively). Additionally, substituents have influence on the rate of the carbonylation and Michael addition steps and both can take place independently. Thus, a favourable balance of the rate between these two reactions is important to achieve successful results. In the reaction of **1** with a tetrasubstituted olefin the initial Michael addition cannot take place, probably due to steric effects and therefore no product was isolated. Other types of Michael acceptors **172c**, **172d** and **172e** were tested leading to unsatisfactory yields, even after a brief screening (of phosphines and bases) to optimize the reaction conditions (14–35%).

**Scheme 57:**
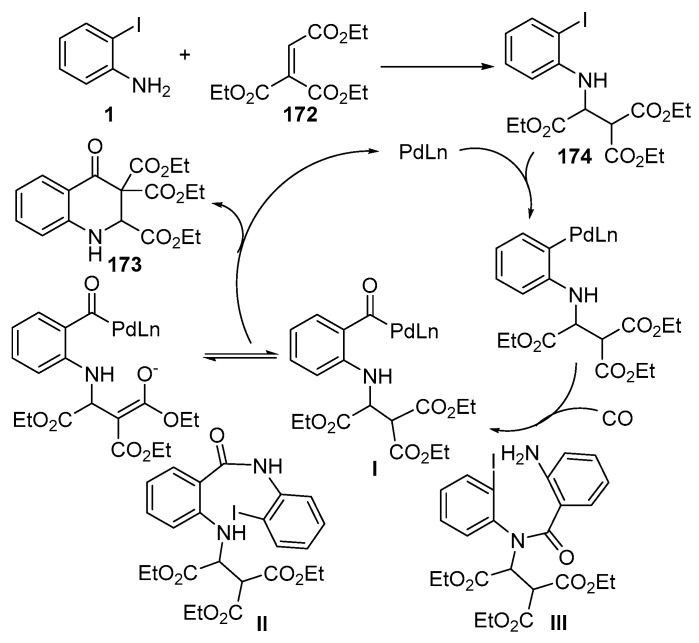
Probable reaction mechanism for the formation of quinolin-4(1*H*)-ones **173** [78].

A plausible reaction mechanism suggests the occurrence of a Michael addition of 2-iodoaniline **1** to diethyl ethoxycarbonylbutendienoate **172**, as the first step, to produce the Michael adduct **174**. Then the phosphine-ligated Pd(0) species undergoes oxidative addition to the C-I bond of **174**, followed by CO insertion giving the aroyl-Pd intermediate I. Nucleophilic attack of the internal malonate anion on this intermediate I ends the catalytic cycle affording quinolin-4(1*H*)-ones **173** regenerating the Pd(0) species. Formation of **175** was explained by intermolecular double carbonylation of **1** with **174** formed in situ. First intermolecular carbonylation takes place between **1** and **174** affording an acyclic amide II and/or III, which can then undergo a second intramolecular carbonylation to give the final product **175** (Scheme 57) [78].

**Scheme 58:**
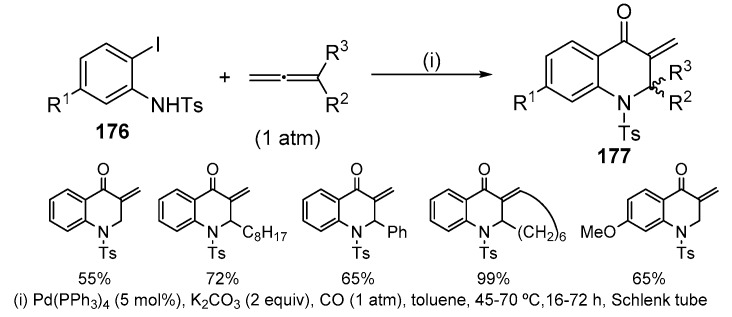
Synthesis of quinolin-4(1*H*)-ones **177** via a Pd-catalysed cascade carbonylation-allene insertion [79].

Quinolin-4(1*H*)-ones **177** were obtained by a Pd(0)-catalysed termolecular queuing process involving oxidative addition to aryl iodide **176**, followed by carbonylation, allene insertion and capture of the resulting п-allyl-Pd(II) species by an internal *N*-nucleophile [79]. CO at atmospheric pressure was used in contrast to Alper′s similar reported cascades [80], which were performed at high pressures of CO (20 atm). Under optimal conditions, using allene (1 atm) and CO (1 atm), a (3 + 1 + 2)-cycloaddition reaction of 2-iodo-1-tosylaniline occurred giving quinolin-4(1*H*)-ones **177** in good yields (55–99%) (Scheme 58). Substituents are tolerated on both the allene and the aryl iodide allowing access to a variety of heterocycles with *s*-*cis*-enone moieties.

**Scheme 59:**
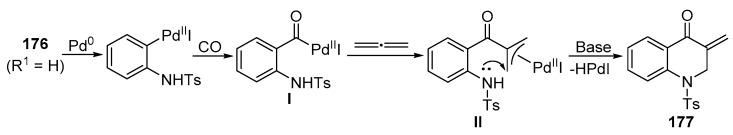
Proposed reaction mechanism for the formation of quinolin-4(1*H*)-ones **177** [79].

**Scheme 60:**
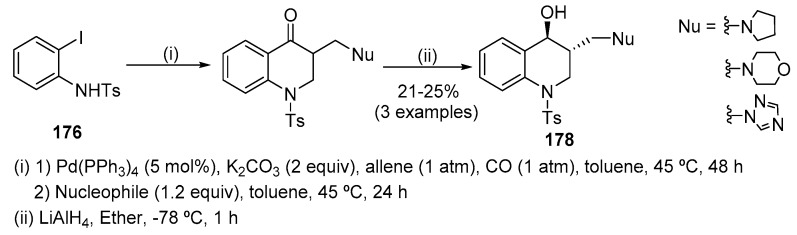
Synthesis of γ-aminoalcohols **178** via Pd-catalysed cascade carbonylation-allene insertion-Michael addition followed by reduction of the corresponding quinolin-4(1*H*)-ones [79].

In the first step of the catalytic cycle, the Pd(0) catalyst undergoes oxidative addition to the aryl iodide **176** bond followed by coordination and insertion of CO. Then addition of acyl-Pd(II) intermediate I to the allene at the central carbon atom originated a п-allyl-Pd(II) species II which undergoes nucleophilic attack by the internal nucleophile to give the enone product **177** (Scheme 59) [79]. These authors also described the one-pot quinolin-4(1*H*)-one synthesis-Michael addition starting from 2-iodo-1-tosylaniline **176**, CO (1 atm) and allene (1atm) in toluene at 45 °C during 48 h. CO was released before the addition of the nucleophile and subsequent Michael addition completed after further 24 h (Scheme 60) [79]. Both aliphatic and heteroaromatic *N*-nucleophiles were effective and in the case of 1,2,4-triazole only the 1-substituted triazole was observed. As the obtained Michael adducts were sensitive to retro-Michael addition, their reduction with LiAlH_4_ was performed without prior isolation, obtaining the corresponding γ-amino alcohols **178** as single stereoisomers.

### 3.5. Oxidative Carbonylation

**Scheme 61:**
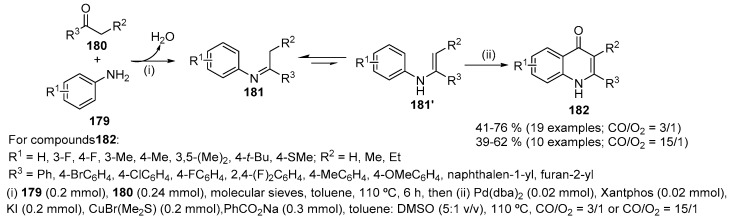
Synthesis of quinolin-4(1*H*)-ones **182** by Pd-catalysed oxidative carbonylation of ketones with amines in the presence of carbon monoxide at atmospheric pressure [81].

Ley and co-workers synthesized quinolin-4(1*H*)-ones **182** via Pd-catalysed oxidative carbonylation starting from simple and commercially available amines **179** and ketones **180** using CO at atmospheric pressure (Scheme 61) [81]. First, an imine **181** is formed from ketone and amine by dehydration condensation. Then, enamine derived from imine/enamine isomerization, undergoes electrophilic attack by Pd(II) to form an intermediate which undergoes intramolecular C−H activation and CO insertion. Subsequent reductive elimination affords the final product and releases Pd(0), which is oxidized by copper and O_2_ to regenerate Pd(II) and complete the catalytic cycle [81]. From different palladium salts, Pd(dba)_2_ was found to be the best option and the use of Xantphos as a ligand improves the product yield. CuBr(Me_2_S), PhCO_2_Na and KI are necessary for the reaction which could also proceed smoothly to give the desired product under nonexplosive conditions (CO/O_2_ = 15/1). For *m*-substituted anilines **179** the reaction with acetophenones gave the desired products in moderate to good yields but with poor selectivity. However, this protocol provides a straightforward route to access useful quinolin-4(1*H*)-ones from inexpensive chemicals. As a wide range of functional groups is well tolerated, various ketones, heterocyclic ketones and amines are workable substrates that can be employed to generate quinolin-4(1*H*)-ones in good yields.

### 3.6. Other Reactions

**Scheme 62:**
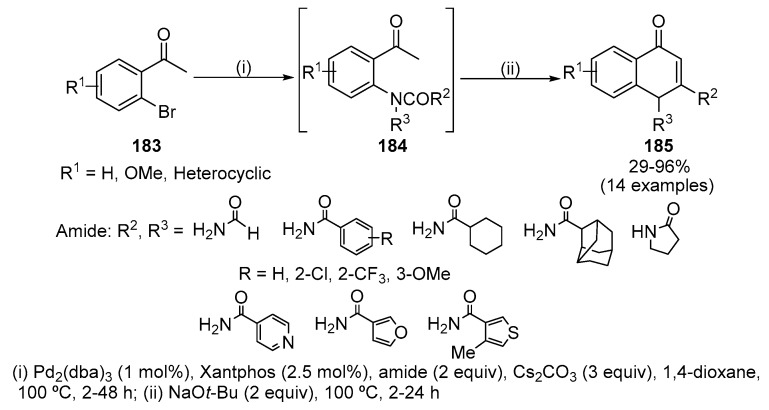
Synthesis of 2-substituted quinolin-4(1*H*)-ones **185** via sequential Pd-catalysed amidation followed by base-promoted intramolecular cyclization [82].

A mild, one pot sequential Pd-catalysed amidation of 2′-bromoacetophenones **183** followed by base-promoted intramolecular cyclization, afforded 2-substituted quinolin-4(1*H*)-ones **185** in 29–96% yields over two steps (Scheme 62) [82]. The best solvent-base combination was 1,4-dioxane/Cs_2_CO_3_. The addition of a strong base, either NaOH or NaO*t*-Bu, was found to be necessary to avoid the hydrolysis of the amide before cyclization. The reaction scope was quite general for both coupling partners although acyclic secondary amides were not successful. Indeed, this method allows the synthesis of a wide range of 2-substituted quinolin-4(1*H*)-ones **185**.

**Scheme 63:**
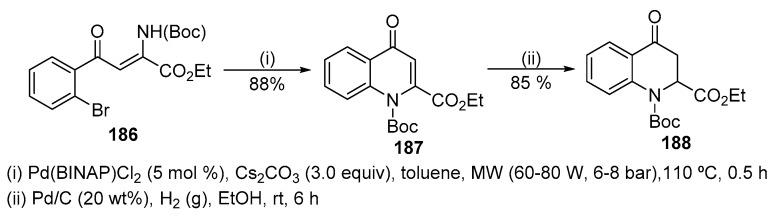
Synthesis of 2,3-dihydroquinolin-4(1*H*)-one **188** by Pd-catalysed intramolecular *N*-arylation of Z-enamine **186** and further reduction [83].

The regioselective Pd-catalysed intramolecular *N*-arylation of (*Z*)-enamine **186** afforded quinolin-4(1*H*)-one **187** in 88% yield, which was further reduced to the corresponding 2,3-dihydroquinolin-4(1*H*)-one **188** (Scheme 63) [83].

**Scheme 64:**
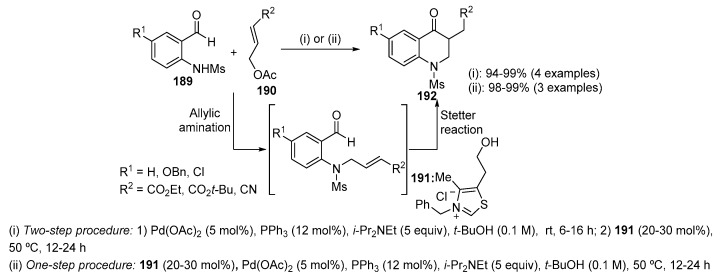
Synthesis of 3-substituted 2,3-dihydroquinolin-4(1*H*)-ones **192** by Pd-catalysed allylic amination-thiazolium salt-catalysed Stetter cascade reaction [84].

Pd-catalysed allylic amination-thiazolium salt-catalysed Stetter reaction between 2-aminobenzaldehydes **189** and allylic acetates **190** is an interesting method to prepare 3-substituted 2,3-dihydroquinolin-4(1*H*)-ones **192** (Scheme 64) [84,85]. The key to the success of this method is the compatibility of the second catalysis with the conditions of the first Pd-catalysis. Under optimized conditions, the one-pot sequential two-step multi-catalytic cascade process afforded 2,3-dihydroquinolin-4(1*H*)-ones **192** in excellent yields (94–99%). This procedure tolerates 2-aminobenzaldehydes **189** bearing electron-donating and electron-withdrawing substituents on the aromatic ring, although, in contrast to that of γ-acetoxy-α,β-unsaturated esters **190** (R^2^ = CO_2_Et, CO_2_*t*-Bu) the reaction did not proceed for γ-acetoxy-α,β-unsaturated nitrile **190** (R^2^ = CN) [Scheme 64, (i)]. When performing the one-pot sequential multi-catalytic cascade process in the presence of both catalysts (one-step procedure), the reaction proceeded well with the three different allylic acetates **190** giving 2,3-dihydroquinolin-4(1*H*)-ones **192** in excellent yields (98–99%), but, on the other hand, the allylic amination occurred only for the unsubstituted 2-aminobenzaldehyde **189**: (R^1^ = H, 98%) [Scheme 64, (ii)].

**Scheme 65:**
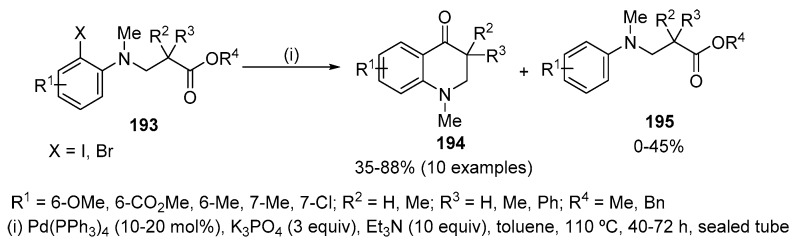
Synthesis of 2,3-dihydroquinolin-4(1*H*)-ones **194** by Pd(0)-catalysed intramolecular coupling of β-(2-iodoanilino)esters **193** [86].

Pd(0)-catalysed intramolecular coupling of β-(2-iodoanilino)esters **193** afforded several 2,3-dihydroquinolin-4(1*H*)-ones **194** in moderate to good yields (Scheme 65) [86]. The use of Pd(PPh_3_)_4_ as the catalyst and Cs_2_CO_3_ as base in THF resulted exclusively in the reduction product **195**. In contrast, using K_3_PO_4_ as base in combination with Et_3_N and toluene led to an increase in the yield of dihydroquinolin-4(1*H*)-ones **194**. These compounds **194** resulted from the nucleophilic substitution at the alkoxycarbonyl group. Substituents on the aromatic ring have little effect on the carbopalladation reaction evidencing that the nucleophilicity of the aryl-Pd species does not appear to be affected by the electronic properties of the substituent. The low yield for the product derived from **193** (R^1^ = CO_2_Me) is mainly a consequence of an increase in the rate of the competitive retro-Michael fragmentation of the β-aminoester. Benzyl ester counterpart of **193** (R^4^ = Bn) can also be used as substrate; however, the desired product is formed in better yields when using methyl ester (R^4^ = Me) (50% and 65%, respectively). The reaction of amino esters **193** without α-hydrogen atoms to the carbonyl group proceeded smoothly to give exclusively the corresponding dihydroquinolin-4(1*H*)-ones **194** in high yields (79–88%). On the other hand, no competition between nucleophilic attack at the carbonyl group and α-arylation was observed in the reactions of amino esters **193** which contain α-hydrogen atoms to the carbonyl group; however, dihydroquinolin-4(1*H*)-ones **194** (35–67%) were obtained together with the reduction products **195** (25–45%).

**Scheme 66:**
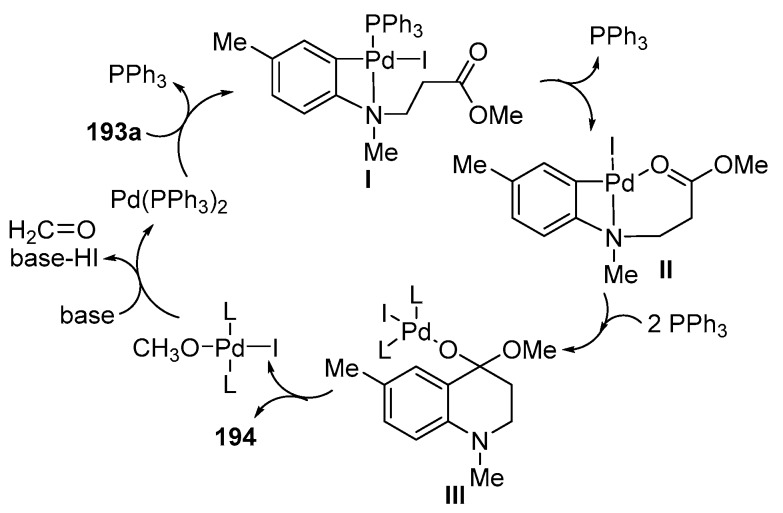
Proposed mechanism for the formation of dihydroquinolin-4(1*H*)-ones **194** [86].

This Pd(0)-catalysed cyclization involves the oxidative addition of the aryl iodide to a Pd(0) species affording a four-membered azapalladacycle I. A carbopalladation between the α-aryl Pd moiety and the alkoxycarbonyl group would give the alkoxide-Pd(II) chelate III. The coordination of the N atom to the Pd centre in I brings the carbonyl group nearer to the metal to facilitate the formation of a transient chelated intermediate II in which the carbonyl group is coordinated to the Pd centre and increases the electron density on the Pd centre to enable the otherwise unfavourable carbopalladation reaction. β-Alcoxide elimination from III would afford the dihydroquinolin-4(1*H*)-one **194** and a Pd(II) alkoxide, which would finally undergo β-hydride elimination regenerating the Pd(0) catalyst. The reduction sequence is supported by the isolation of significant amounts of benzaldehyde in the reaction of benzyl ester **193** (R^4^ = Bn) (Scheme 66).

## 4. Transformation of Quinolin-2(1*H*)-ones

### 4.1. Heck Reaction

**Scheme 67:**
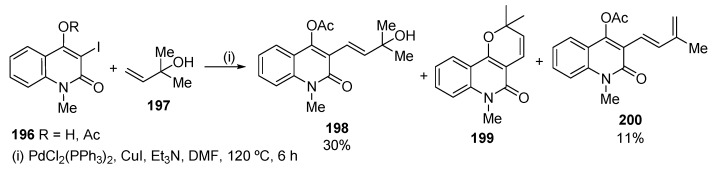
Heck reaction of 3-iodoquinolin-2(1*H*)-one **196** with 2-methyl-3-buten-2-ol **197** [87].

Pd-catalysed Heck reaction of 3-iodoquinolin-2(1*H*)-one **196** with 2-methyl-3-buten-2-ol **197** afforded the quinolone dimers paraensidimerins′ precursor **198** (Scheme 67) [87]. The reaction of 4-acetoxyquinolin-2(1*H*)-one **196** (R = Ac), obtained by acetylation of 4-hydroxy-3-iodo-1-methyl quinolin-2(1*H*)-one **196** (R = H), gave three products: (i) the expected quinolone allylic alcohol **198** (30%); (ii) the diene **200** (11%), probably obtained by dehydration of allylic alcohol **198** by the Et_3_NHI salt by-product formed in the reaction mixture; and (iii) the known alkaloid *N*-methylflindersine **199**, probably formed by deacetylation and subsequent intramolecular cyclization during the course of the reaction.

**Scheme 68:**
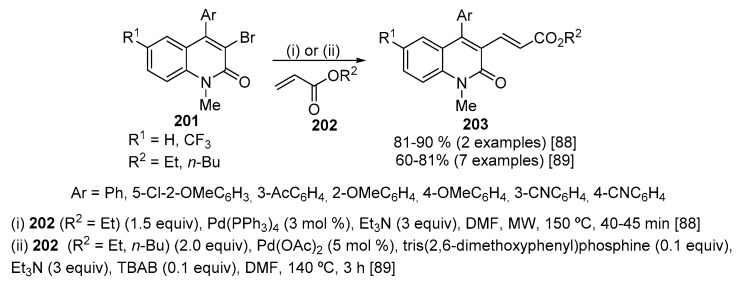
Synthesis of quinolin-2(1*H*)-ones **203** via Heck cross-coupling reaction [88,89].

Successful Heck vinylations of 4-aryl-3-bromo-1-methylquinolin-4(1*H*)-ones **201** with acrylates **202** were achieved by Kappe and co-workers using Pd(PPh_3_)_4_ and Et_3_N in DMF, affording 3-substituted-quinolin-4(1*H*)-ones **203** (Scheme 68) [88]. Similar transformations were performed by Wu and co-workers using a different catalyst system, Pd(OAc)_2_ in the presence of tris(2,6-dimethoxyphenyl)phosphine and TBAB, using also Et_3_N and DMF (Scheme 68) [89].

**Scheme 69:**
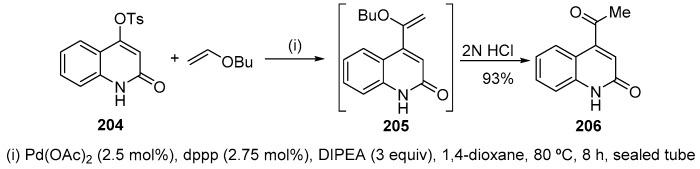
Synthesis of 4-acetylquinolin-2(1*H*)-one **206** via Heck reaction of 4-tosylquinolin-2(1*H*)-one **204** with butyl vinyl ether followed by acidic treatment [90].

The highly α-regioselective Heck coupling of 4-tosylquinolin-2(1*H*)-one **204** with butyl vinyl ether afforded 4-acetylquinolin-2(1*H*)-one **206** in very good yield (93%) (Scheme 69) [90]. The α-product **205** was obtained in very high α/β regioselectivity (>99/1) and after acidic treatment led to 4-acetylquinolin-2(1*H*)-one **206**. Although α-regioselectivity in Heck coupling can be obtained with triflates as substrates, tosylates are less toxic, less expensive and, importantly, more stable.

**Scheme 70:**
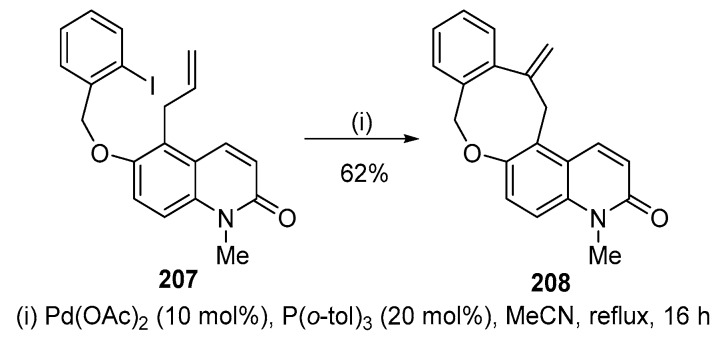
Synthesis of benzoxocine-fused quinolin-2(1*H*)-one **208** by intramolecular Heck reaction of benzylallylquinolin-2(1*H*)-one **207** [91].

The intramolecular Heck reaction of the benzylallylquinolin-2(1*H*)-one precursor **207** originated a benzoxocine-fused quinolin-2(1*H*)-one **208** (Scheme 70) [91]. The reaction led to the regioselective formation of the 8-*exo* cyclization product **208** and no 9-*endo* product was observed in this case.

**Scheme 71:**
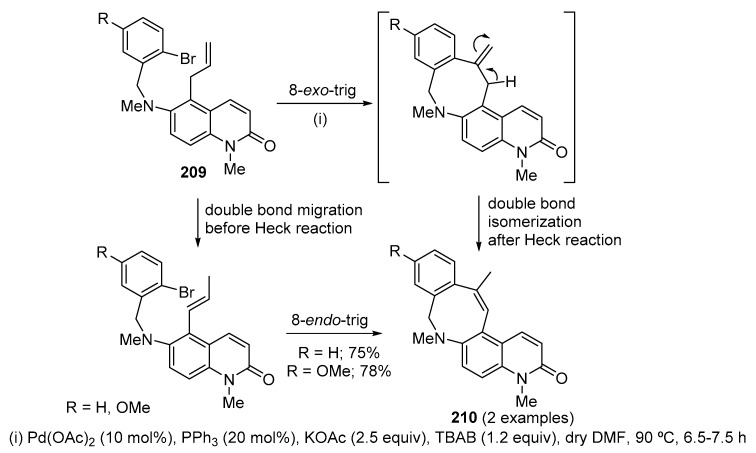
Probable mechanistic pathway for the synthesis of quinolin-2(1*H*)-one annulated benzoazocines **210** by intramolecular Heck reaction of allylic quinolin-2(1*H*)-ones **209** [92].

Majumdar and co-workers reported a Pd-catalysed intramolecular Heck reaction of inactivated allylic quinolin-2(1*H*)-ones **209** that led to the formation of quinolin-2(1*H*)-one annulated benzoazocines **210** (Scheme 71) [92]. The intramolecular Heck reaction afforded exclusively the *endo*-cyclic product **210** in good yields. Once exclusively cyclization via the 8-*exo* mode is unusual, in this case, it was suggested that the formation of the *endo*-cyclic products may occur via two possible pathways; the 8-*exo* mode of cyclization followed by double-bond isomerization, or, alternatively, a double-bond isomerization prior to the Heck reaction leading to the subsequent 8-*endo*-Heck cyclization (Scheme 71) [92].

**Scheme 72:**
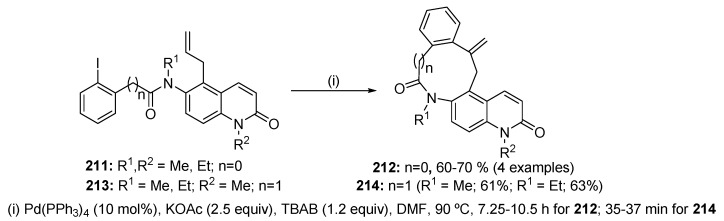
Synthesis of quinolin-2(1*H*)-one annulated benzazocinones **212** and quinolin-2(1*H*)-one annulated benzazoninones **214** by intramolecular Heck reaction of allylic quinolin-2(1*H*)-ones **211** and **213** [93,94].

Later, the same authors described the synthesis of quinolin-2(1*H*)-one annulated benzazocinones **212** by applying the same methodology to inactivated allylic quinolin-2(1*H*)-ones **211** (Scheme 72) [93]. In this case, the optimal reaction conditions required Pd(PPh_3_)_4_ as the catalyst. This highly regioselective ligand-free Heck coupling reaction leads exclusively to the expected 8-*exo*-Heck product **212**, in good yields, without any contamination of the 9-*endo*-Heck or 8-*exo*-isomerized product. The formation of the 8-*exo*-Heck product is favoured because of the lower steric and transannular interactions. These authors have also prepared two quinolin-2(1*H*)-one annulated benzazoninones **214** starting from inactivated allylic quinolin-2(1*H*)-ones **213** (Scheme 72) [94]. The Heck reaction proceeded in reasonably good yields with shorter reaction times and only the product corresponding to the 9-*exo* mode of cyclization was obtained.

**Scheme 73:**
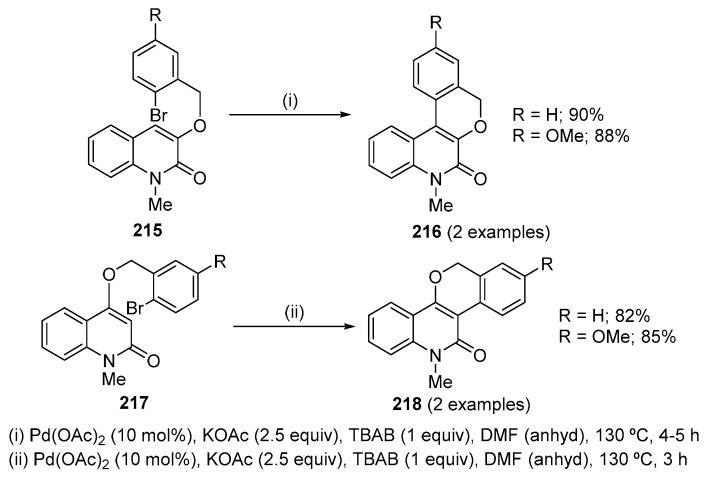
Synthesis of tetracyclic quinolones **216** and **218** by intramolecular Heck reaction of 3- and 4-(2-bromobenzyloxy)quinolin-2(1*H*)-ones **215** and **217** [97].

Pd-catalysed intramolecular Heck cyclization of previously synthesized 3- and 4-(2-bromobenzyloxy)quinolin-2(1*H*)-ones **215** and **217** [95,96], under Jeffery’s two-phase protocol afforded four tetracyclic quinolones **216** and **218** in very good yields (Scheme 73) [97].

**Scheme 74:**
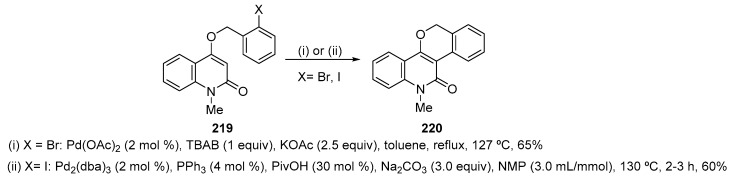
Synthesis of 12-methyl-6*H*-isochromeno[4,3-*c*]quinolin-11(12*H*)-one **220** by intramolecular direct arylation of quinolin-2(1*H*)-ones **219** [98,99].

Intramolecular regioselective arylation of *N*-methylquinolin-2(1*H*)-one **219** (X = Br) was achieved using a simplified catalyst system, Pd(OAc)_2_, TBAB and KOAc in toluene and Jeffrey’s conditions for the Mizoroki-Heck-type reaction, affording 12-methyl-6*H*-isochromeno[4,3-*c*] quinolin-11(12*H*)-one **220** in 65% yield (Scheme 74) [98]. The same compound **220** was obtained, in good yield (60%), by intramolecular direct arylation of quinolin-2(1*H*)-one **219** (X = I) which was performed using a Pd(0) source and pivalic acid as a crucial additive (Scheme 74) [99]. Mechanistic studies suggested that the reaction proceeds through an initial oxidative addition followed by a pivalate assisted Concerted-Metalation-Deprotonation (CMD)-type mechanism, although a S_E_Ar route was not ruled out [99].

**Scheme 75:**
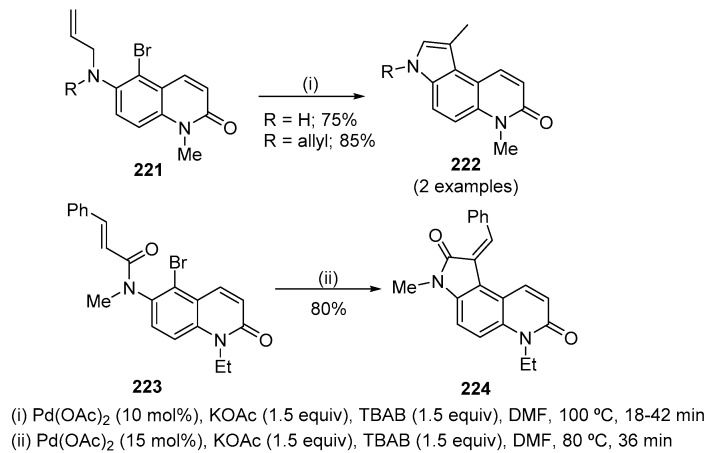
Synthesis of pyrrolo[3,2-*f*]quinolin-7(6*H*)-ones **222** and **224** by intramolecular Heck reaction of 5-bromoquinolin-2(1*H*)-ones **221** and **223** [100].

A ligand free intramolecular Heck reaction of 6-amino-5-bromoquinolin-2(1*H*)-ones **221** catalysed by Pd(OAc)_2_ gave new pyrrole-fused quinolin-2(1*H*)-ones **222** (Scheme 75) [100]. This methodology is applicable to secondary and tertiary amines. Under similar reaction conditions the intramolecular Heck reaction of quinolin-2(1*H*)-one **223** afforded the corresponding fused quinolin-2(1*H*)-one **224**.

**Scheme 76:**
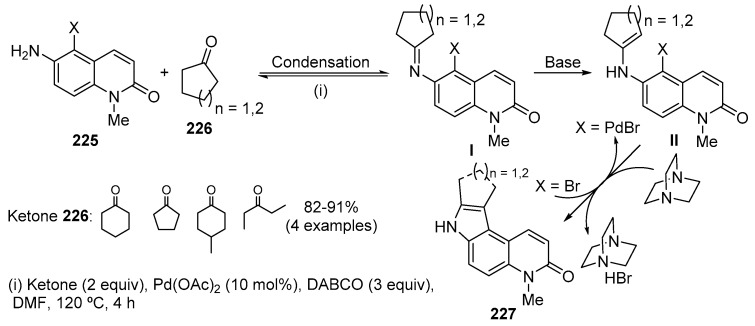
Proposed mechanism for the formation of pyrrolo[3,2-*f*]quinolin-7(6*H*)-ones **227** from intramolecular Heck reaction of enamines prepared in situ [101].

Other pyrrolo[3,2-*f*]quinolin-7(6*H*)-ones **227** were synthesized, in very good yields, by ligand free Pd(OAc)_2_-catalyzed intramolecular Heck reaction of enamines, prepared in situ from the condensation of 6-amino-5-bromo-1-methylquinolin-2(1*H*)-ones **225** with cyclic and acyclic ketones **226** (Scheme 76) [101]. The reaction was only effective when using DMF as solvent and other Pd sources [PdCl_2_, PdCl_2_(PPh_3_)_2_, Pd(PPh_3_)_4_] were less efficient than Pd(OAc)_2_. From the screened bases (DABCO, KOAc, NaOAc, Et_3_N), DABCO was found the most suitable and it was not necessary to protect the amino group. First, substrates **225** condense with the ketone to generate I and produce the enamines II in the presence of base. The formed enamines subsequently underwent intramolecular Pd-catalysed Heck reaction to afford the products **227** (Scheme 76).

### 4.2. Suzuki-Miyaura Reaction

**Scheme 77:**
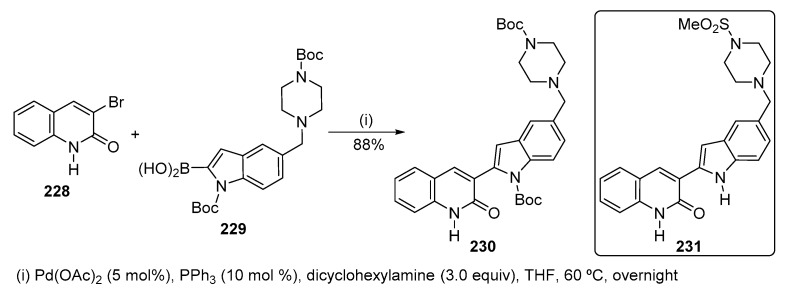
Synthesis of indol-2-ylquinolin-2(1*H*)-one **230** by Suzuki-Miyaura cross-coupling reaction of quinolin-2(1*H*)-one **228** with boronic acid derivative **229** [102].

An efficient convergent synthesis of the potent and selective KDR inhibitor 3-{5-[[4-(methylsulfonyl)-1-piperazinyl]methyl]-1*H*-indol-2-yl}quinolin-2(1*H*)-one **231** involved a Suzuki coupling of two substrates **228** and **229**, as the main step to construct the indol-2-ylquinolin-2(1*H*)-one moiety **230**, the key pharmacophore of this type of KDR inhibitors. The cross-coupling was run in THF with Pd(OAc)_2_ (0.5 mol%) and PPh_3_; however, the usual basic aqueous system led to substantial deboronation. Dicyclohexylamine was found to be an excellent activator and slow addition of boronic acid to the catalyst/bromoquinolinone mixture minimized deboronation (Scheme 77) [102]. Formerly, Fraley and co-workers have also adopted the Suzuki-Miyaura cross-coupling of diversely substituted indolylboronic acids with 3-iodoquinolin-2(1*H*)-ones, using Pd(PPh_3_)_4_ as catalyst, to synthesize other related KDR inhibitors [103].

**Scheme 78:**
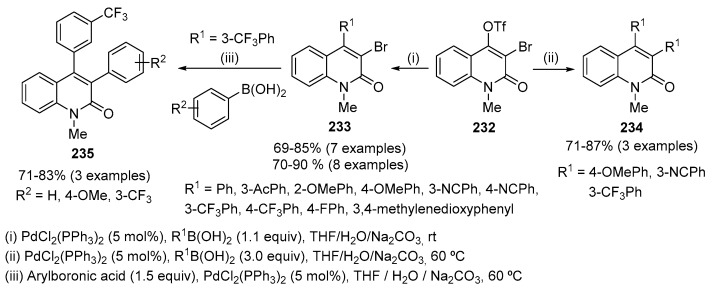
Synthesis of 4- and 3,4-substituted quinolin-2(1*H*)-ones **233** and **234** via regioselective Suzuki-Miyaura reactions of 3-bromo-4-trifloxyquinolin-2(1*H*)-ones **232** and further transformation of quinolin-2(1*H*)-one **233** [104,105].

Pd-catalysed cross-coupling reactions of 3-bromo-4-trifloxyquinolin-2(1*H*)-ones **232** with arylboronic acids afforded 4- and 3,4-substituted quinolin-2(1*H*)-ones **233** and **234** (Scheme 78) [104]. Regiocontrolled cross-coupling reaction of 3-bromo-4-trifloxyquinolin-2(1*H*)-one **232** was achieved by tuning the temperature and the amount of arylboronic acid. When using PdCl_2_(PPh_3_)_2_ as catalyst in the reaction with 4-methoxyphenylboronic acid (1.5 equiv) at 50 °C, the corresponding product **233** was obtained in 20% yield together with disubstituted compound **234** (60%). At room temperature, **233** was obtained as the major product (78%) and only traces of **234** were detected. Reducing the amount of 4-methoxyphenylboronic acid to 1.1 equiv, **233** was the only product (81%). At 60 °C with a higher excess of 4-methoxyphenylboronic acid (2.5–3.0 equiv), only **234** was generated (87%). Both electron-withdrawing and electron-donating-substituted arylboronic acids are suitable coupling partners, giving similar reaction yields. Compound **233** (R^1^ = 3-CF_3_Ph) was further elaborated by Suzuki-Miyaura reaction with different arylboronic acids affording the corresponding products **235** in good yields (Scheme 78) [105]. The reaction of 3-bromo-4-trifloxyquinolin-2(1*H*)-one **232** with arylboronic acids, catalysed by PdCl_2_(PPh_3_)_2_, afforded 4-aryl-3-bromoquinolin-2(1*H*)-ones **233** (Scheme 78) [105]. Various aryl groups could be easily introduced at the 4-position of quinolin-2(1*H*)-one scaffold in good to excellent yields.

**Scheme 79:**
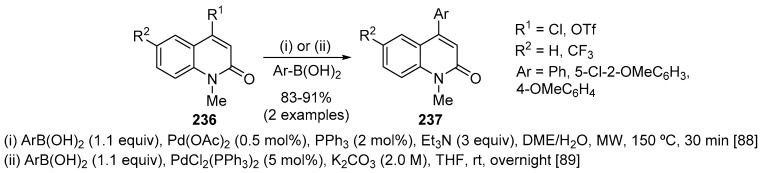
Synthesis of 4-arylquinolin-2(1*H*)-ones **237** via Suzuki-Miyaura cross-coupling reaction of quinolin-2(1*H*)-ones **236** with arylboronic acids [88,89].

Derivatization of 4-chloro- and 4-trifloxy-1-methylquinolin-4(1*H*)-ones **236**, by introducing 4-aryl substituents, was performed by Pd-catalysed Suzuki-Miyaura reaction. The best conversions and isolated product yields (83–91%), for the reaction of **236** (R = Cl) with arylboronic acids, were achieved by using a combination of Pd(OAc)_2_ (5 mol%), PPh_3_, a 3:1 mixture of 1,2-dimethoxyethane (DME):water as solvent, together with either Na_2_CO_3_ or Et_3_N as base, at 150 °C (Scheme 79) [88]. When employing **236** (R = OTf) as electrophile, the coupling product was isolated in 88% yield, using PdCl_2_(PPh_3_)_2_, Na_2_CO_3_ and a mixture of THF and water as solvent (Scheme 79) [89]. The presence of water in the solvent mixture was found to be crucial for these reactions to proceed properly.

**Scheme 80:**
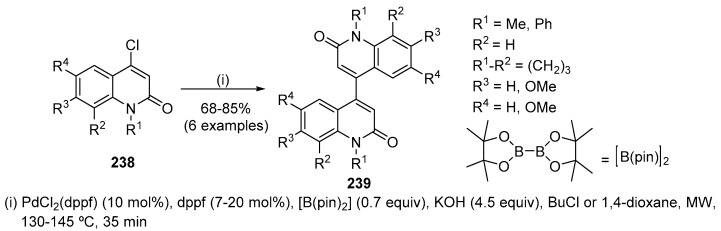
Synthesis of 4,4′-bisquinolones **239** by one-pot borylation-Suzuki cross-coupling of 4-chloroquinolin-2(1*H*)-ones **238** [106].

Pd-catalysed one-pot borylation-Suzuki cross-coupling reaction of 4-chloroquinolin-2(1*H*)-ones **238**, under controlled MW irradiation, gave functionalized 4,4′-bisquinolones **239** in good to excellent yields (Scheme 80) [106]. Excellent conversions were achieved using a strong base such as KOH in combination with PdCl_2_(dppf). Among the tested solvents, DMSO, DMF or toluene promote the formation of the dehalogenated product while 1,4-dioxane, dichloromethane and 1,2-dichloroethane (DCE) minimized dehalogenation; however, 1-chlorobutane proved to be the best solvent in this particular case.

**Scheme 81:**
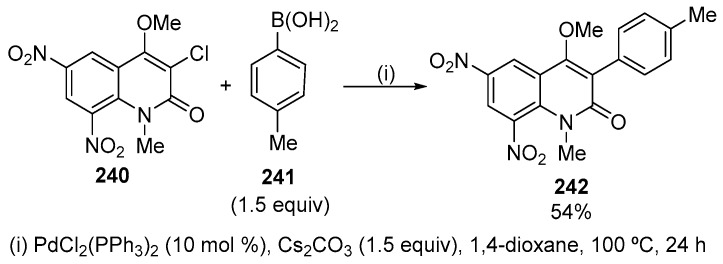
Synthesis of quinolin-2(1*H*)-one **242** by Suzuki-Miyaura cross-coupling of dinitroquinolin-2(1*H*)-one **240** with 4-methylphenylboronic acid **241** [107].

Dinitroquinolin-2(1*H*)-one **240** was subjected to Suzuki-Miyaura cross-coupling reaction with 4-methylphenylboronic acid **241**. In the presence of PdCl_2_(PPh_3_)_2_ as catalyst, the arylated product **242** was obtained in moderate yield (Scheme 81) [107].

### 4.3. Sonogashira Reaction

**Scheme 82:**
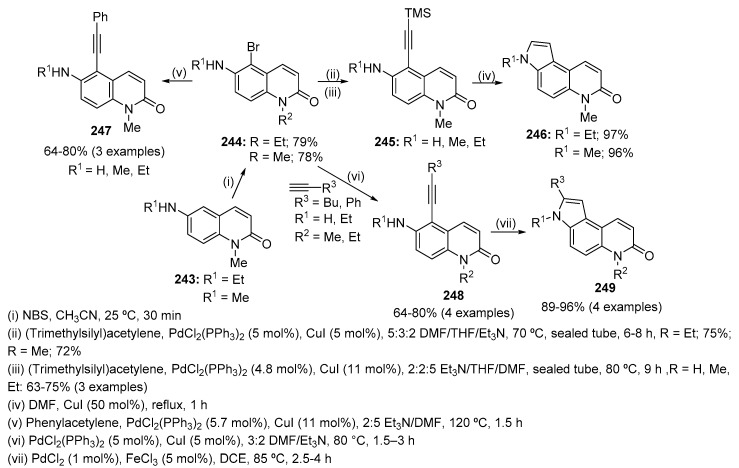
Sonogashira coupling of 6-amino-5-bromoquinolin-2(1*H*)-ones **244** with different acetylenes and cyclization of the resulting compounds into pyrrolo[3,2-*f*]quinolin-7(6*H*)-ones **246** and **249** [108,111,112].

Sequential coupling and cyclization reactions of aryl halides with (trimethylsilyl)acetylene with concurrent elimination of the trimethylsilyl (TMS) substituent furnished pyrrolo[3,2-*f*]quinolin-7(6*H*)-ones **246** in excellent yields (Scheme 82) [108]. Acetylenic amines possessing an electron-donating group on the nitrogen atom also underwent Cu(I)-catalysed cyclization. Quinolones **243** were prepared from commercially available quinolines [109,110], then brominated and the resulting bromo-derivatives **244** were transformed into the required precursors for heteroannulation **245** by a Sonogashira coupling with (trimethylsilyl)acetylene using PdCl_2_(PPh_3_)_2_ as catalyst and CuI as the co-catalyst in THF/DMF mixed solvent containing Et_3_N. The reactions were optimized by smoothly heating the reaction in a sealed tube. Heteroannulation of the acetylenic amines to give **246** was achieved by refluxing the precursors **245** in DMF in the presence of 50 mol% of CuI. Another work reported the synthesis of acetylenic amines **245** and **247** by Sonogashira coupling of the corresponding 6-amino-5-bromoquinolin-2(1*H*)-ones **244** with (trimethylsilyl)acetylene or phenylacetylene, respectively. Both reactions were performed using PdCl_2_(PPh_3_)_2_ as catalyst and CuI as co-catalyst, although in slightly different experimental conditions (Scheme 82) [111].

Acetylenic amines **248**, obtained from **244** by a Sonogashira coupling, underwent an intramolecular hydroamination reaction, catalysed by PdCl_2_/FeCl_3_, to give pyrrolo[3,2-*f*]quinolin-7(6*H*)-ones **249** (Scheme 82) [112]. PdCl_2_(PPh_3_)_2_ and Pd(OAc)_2_ were also tested as Pd(II) sources but were ineffective, unlike PdCl_2_ that was efficient in only 1 mol%. FeCl_3_ is necessary for this hydroamination reaction to occur and may facilitate the reoxidation of Pd(0) to Pd(II) in the catalytic cycle. This cyclization proceeded well in 1,2-dichloroethane at reflux, in the presence of aromatic- and aliphatic-substituted alkynes and with both protected and unprotected amines.

**Scheme 83:**
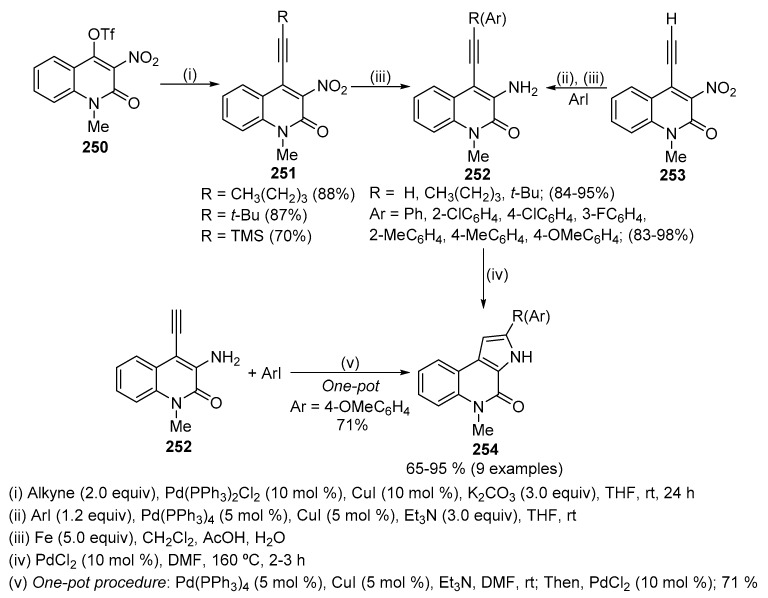
Synthesis of 3*H*-pyrrolo[2,3-*c*]quinolin-4(5*H*)-ones **254** from 1-methyl-3-nitro-4-trifloxyquinolin-2(1*H*)-one **250** by Pd-catalysed Sonogashira cross-coupling reaction and cyclization [113].

Pd-catalysed Sonogashira reaction of 1-methyl-3-nitro-4-trifloxyquinolin-2(1*H*)-one **250** with various alkynes, in the presence of Pd(PPh_3_)_2_Cl_2_/CuI in THF at room temperature, using K_2_CO_3_ as the base, afforded the coupling products **251** in moderate to good yields (Scheme 83) [113]. Aliphatic alkynes are excellent substrates, whereas no product was detected when using aromatic alkynes. A strategy was developed to install aromatic substituents at the alkynes moiety at the C-4 of the quinolin-2(1*H*)-one core by subjecting compound **253** (R = H) to Sonogashira coupling with aryl iodides. This cross-coupling reaction afforded the corresponding 3-amino-4-arylethynyl quinolin-2(1*H*)-ones **252** in high yields with various aryl iodides. Reduction of the nitro group of **251**, mediated by iron powder in AcOH/H_2_O at room temperature, generated the 4-alkynyl-3-aminoquinolin-2(1*H*)-ones **252** in excellent yields. Finally the intramolecular hydroamination of 3-amino-4-aryl-ethynylquinolin-2(1*H*)-ones **252** in DMF, in the presence of PdCl_2_, gave the desired 3*H*-pyrrolo[2,3-*c*]quinolin-4(5*H*)-ones **254** in moderate to good yields; however, arylacetylenic substrates with electron-withdrawing groups on the R position were less reactive than those with electron-donating groups (Scheme 83) [113]. The one-pot reaction between 3-amino-4-ethynylquinolin-2(1*H*)-one **252** (R = H) and *p*-iodoanisole was also successfully achieved affording 3*H*-pyrrolo[2,3-c]quinolin-4-(5*H*)-one **254** in 71% yield (Scheme 83) [113].

**Scheme 84:**
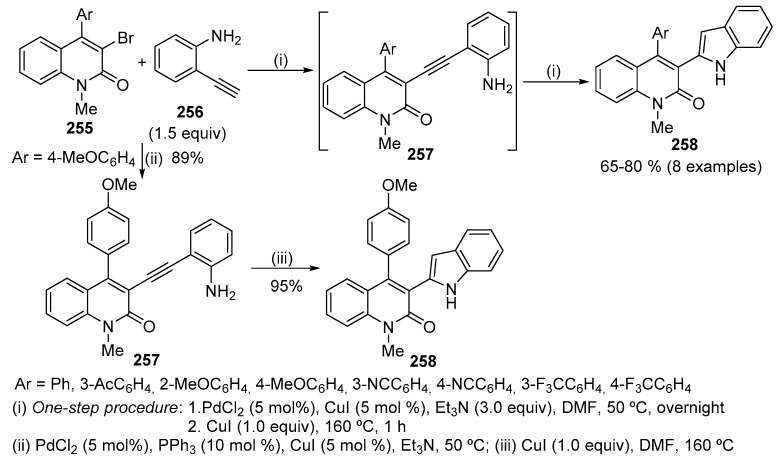
Synthesis of 1*H*-indol-2-yl-(4-aryl)quinolin-2(1*H*)-ones **258** via Sonogashira coupling of quinolin-2(1*H*)-ones **255** with 2-ethynylaniline **256** and CuI-mediated cyclization of **257** [105].

According to Wu and co-workers, the Sonogashira reaction of quinolin-2(1*H*)-ones **255** with 2-ethynylaniline **256** proceeded smoothly in the presence of PdCl_2_ (5 mol%), CuI (5 mol%) and PPh_3_ (10 mol%) in Et_3_N at 50 °C to afford the desired products **257** in 89% yield. CuI-mediated cyclization of **257** led to the desired 1*H*-indol-2-yl-(4-aryl)quinolin-2(1*H*)-ones **258** in very good yield (95%) (Scheme 84) [105]. These authors have also attempted the one-pot reaction of 3-bromo-4-(4-methoxyphenyl)quinolin-2(1*H*)-one **255** (Ar = 4-MeOC_6_H_4_) with 2-ethynylaniline **256**. After completion of the reaction of **255** with **256**, CuI (1.0 equiv) was added directly in the reaction mixture and it was stirred at 160 °C for another 1 h affording the expected product **257** (Ar = 4-MeOC_6_H_4_) in 79% yield. They have demonstrated the generality of this one-pot procedure by preparing a series of other derivatives which were obtained in moderate to good yields (65–80%) (Scheme 84) [105].

**Scheme 85:**
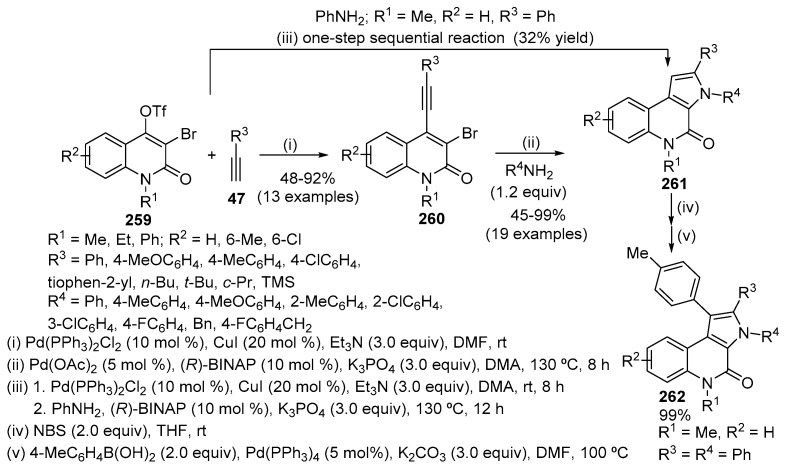
Synthesis of 3*H*-pyrrolo[2,3-*c*]quinolin-4(5*H*)-ones **261** via Pd-catalysed domino reaction of 4-alkynyl-3-bromoquinolin-2(1*H*)-ones **260** [114].

4-Alkynyl-3-bromoquinolin-2(1*H*)-ones **260**, prepared by Sonogashira reaction of 3-bromo-4-trifloxyquinolin-2(1*H*)-ones **259** with terminal alkynes **47**, underwent Pd-catalysed domino reaction with amines affording 3*H*-pyrrolo[2,3-*c*]quinolin-4(5*H*)-ones **261** (Scheme 85) [114]. Both aryl and alkyl substituents in the quinolin-2(1*H*)-one **260** and in the terminal alkyne **47** were well tolerated with exception of trimethylsilyl acetylene. The electronic nature of the arylamine does not influence the outcome of the domino reaction. In addition, *o*-substituted anilines are well tolerated. Substrates **260** with an alkyl group as R^1^ were more reactive than with an aryl group. An attempt to perform the synthesis of 3*H*-pyrrolo[2,3-*c*]quinolin-4(5*H*)-one **261** in a one-pot and sequential manner, from of 3-bromo-4-trifloxyquinolin-2(1*H*)-one **259**, phenylacetylene and aniline, afforded the expected product in 32% yield. Diversity of 3*H*-pyrrolo[2,3-*c*]quinolin-4(5*H*)-ones **261** was increased after a bromination of pyrrole ring with NBS and Suzuki coupling with *p*-tolylboronic acid that led to 5-methyl-2,3-diphenyl-1-(*p*-tolyl)-3*H*-pyrrolo[2,3-*c*]quinolin-4(5*H*)-one **262** in 99% yield (Scheme 85) [114].

### 4.4. Buchwald-Hartwig Reaction

**Scheme 86:**
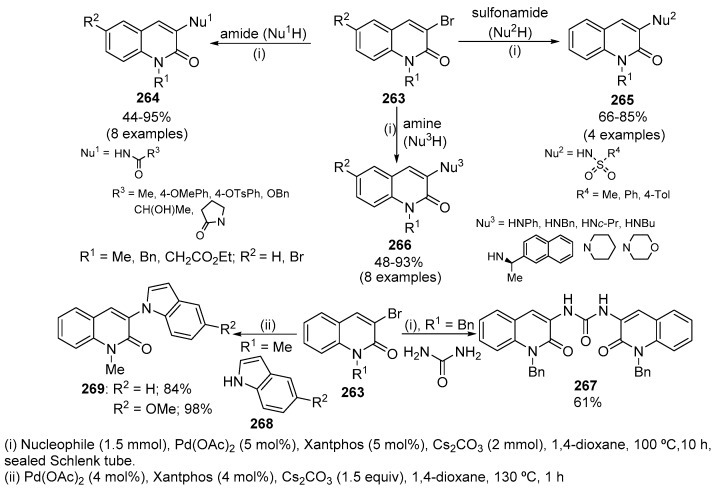
Synthesis of 3-(*N*-substituted)-aminoquinolin-2(1*H*)-ones **264**–**267** and **269** by Pd-catalysed C–N coupling reaction of 3-bromoquinolin-2(1*H*)-ones **263** [115,116].

Pd-catalysed C–N coupling reaction, starting from 3-bromoquinolin-2(1*H*)-ones **263**, originated 3-(*N*-substituted)aminoquinolin-2(1*H*)-ones **264**–**267** in good to excellent yields (Scheme 86) [115]. Several nucleophiles, including amines, amides and benzylcarbamate, as well as the less nucleophilic alkyl- or arylsulfonamides, were effective in this transformation. In all the cases, the reactions occurred rapidly in 1,4-dioxane, using Pd(OAc)_2_ as a catalyst, Xantphos as a ligand and Cs_2_CO_3_ as a base (Scheme 86). In the presence of two carbon–bromine bonds, as in the quinolin-2-(1*H*)-ones **263** (R^2^ = Br), the coupling proceeded at the more activated C-3 position and yielded the mono-substituted product (44–48%) together with the disubstituted one (15–27%). The C-N bond forming reaction was also performed with urea, in the same experimental conditions, leading to the double heteroarylated coupling product **267** in good yield (61%) (Scheme 86) [115]. Also, Pd-catalysed coupling reaction of 3-bromo-1-methylquinolin-2(1*H*)-one **263**, as the electrophilic partner, with indole and 5-methoxyindoles **268** afforded the coupling products **269** in excellent yields (Scheme 86) [116].

### 4.5. Aminocarbonylation Reaction

**Scheme 87:**
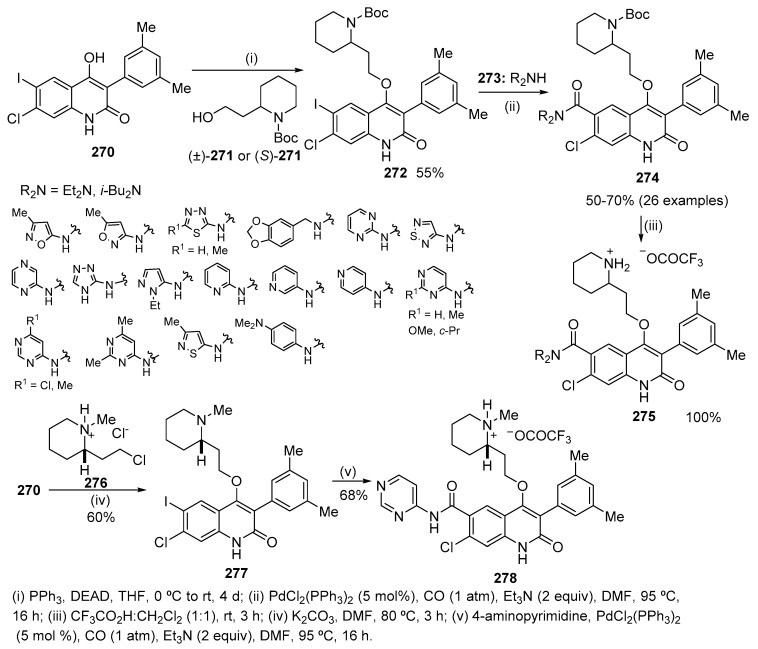
Synthesis of quinolin-2(1*H*)-one-6-carboxamides **275** and **278** by Pd-catalysed aminocarbonylation of 4-hydroxyquinolin-2(1*H*)-one **270** with diverse amines **273** and 4-aminopyrimidine [117].

Pd-catalysed aminocarbonylation of 4-hydroxyquinolin-2(1*H*)-one **270** afforded quinolin-2(1*H*)-one-6-carboxamides **275** which are potent antagonists of gonadotropin releasing hormone receptors [117]. *O*-Alkylation of **270** with either (±)-**271** or (*S*)-**271** using the Mitsunobu protocol afforded the *O*-alkyl ethers (±)-**272** and (*S*)-**272**. Then Pd-catalysed aminocarbonylation of the iodo compound **272** with a variety of primary and secondary amines **273** led to the protected quinolin-2(1*H*)-one-6-carboxamides **274**. Removal of Boc protecting group of piperidines **274** using trifluoroacetic acid afforded the targeted amides **275** in quantitative yields (Scheme 87) [117]. Quinolin-2(1*H*)-one-6-carboxamide **278** was also prepared by alkylation of 4-hydroxyquinolin-2(1*H*)-one **270** with (*S*)-2-(2-chloroethyl)-1-methylpiperidine **276** followed by aminocarbonylation of intermediate **277** with 4-aminopyrimidine (Scheme 87) [117].

**Scheme 88:**
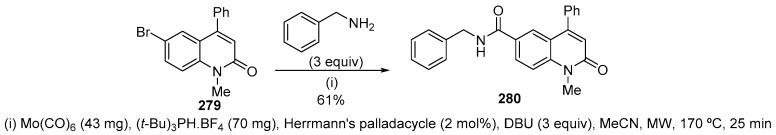
Synthesis of quinolin-2(1*H*)-one **280** via aminocarbonylation of 6-bromo-4-phenylquinolin-2(1*H*)-one **279** with benzylamine [88].

Kappe and co-workers carried out the microwave-assisted aminocarbonylation of 6-bromo-4-phenylquinolin-2(1*H*)-one **279** with benzylamine. The reaction was performed using molybdenum hexacarbonyl as a solid source of CO and Herrmann’s palladacycle in combination with Fu′s salt [(*t*-Bu)_3_PH.BF_4_] as a catalyst system, in acetonitrile at 170 °C for 25 min. affording the expected amide **280** in 61% yield (Scheme 88) [88,89].

**Scheme 89:**
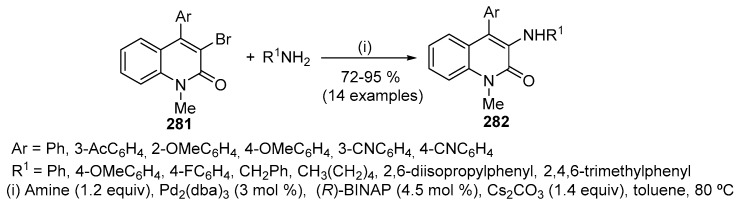
Pd-catalysed reaction of 4-aryl-3-bromoquinolin-2(1*H*)-one **281** with amines [89].

Aminocarbonylation of 4-aryl-3-bromoquinolin-2(1*H*)-one **281** under the conditions reported by Wu and co-workers, [Pd_2_(dba)_3_ (3 mol%), (*R*)-BINAP (4.5 mol%), Cs_2_CO_3_, toluene, 80 °C], afforded the expected 3-amino derivatives **282** in good to excellent yields (Scheme 89) [89]. Similar results were obtained for electron-donating and electron-withdrawing groups attached on the aromatic ring of substrates. Aliphatic amines were found to be good partners whereas no reaction occurred with bulky amines.

### 4.6. Other Reactions

**Scheme 90:**
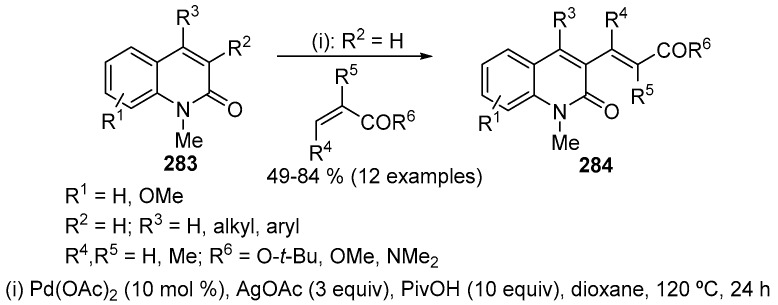
Synthesis of 3-alkenyl-4-substituted-quinolin-2(1*H*)-ones **284** by Pd-catalysed intermolecular C-H alkenylation with acrylates or acrylamide [45].

Quinolin-2(1*H*)-ones **283** were functionalized at the 3-position through an intermolecular C-H alkenylation reaction with acrylates or acrylamide, in the presence of Pd(OAc)_2_ and AgOAc as oxidant, leading to the corresponding 3-alkenyl-4-substituted-quinolin-2(1*H*)-ones **284** in good yields and with complete regio- and stereoselectivity, even with 4-unsubstituted quinolin-2(1*H*)-ones (Scheme 90) [45].

**Scheme 91:**
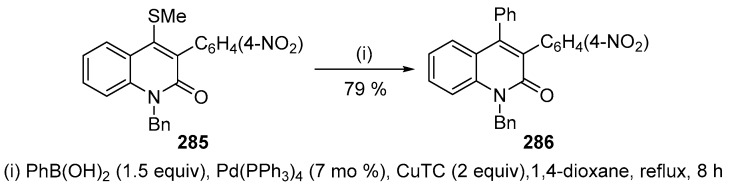
Functionalization of alkylthioquinolin-2(1*H*)-one **285** by Pd-catalysed cross-coupling reaction with phenylboronic acid [56].

The alkylthio functional group of quinolin-2(1*H*)-one **285** was functionalized by Pd-catalysed cross-coupling with phenylboronic acid in the presence of copper-(I)-thiophene-2-carboxylate (CuTC) affording 4-phenylquinolin-2(1*H*)-one **286** in 79% yield (Scheme 91) [56].

**Scheme 92:**
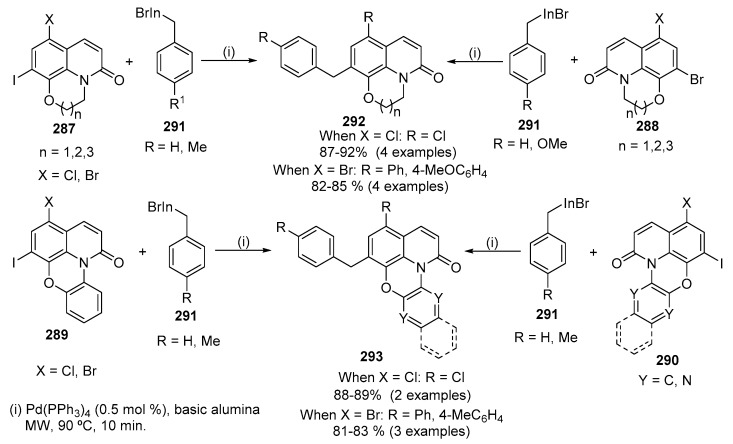
Basic alumina-supported synthesis of quinolin-2(1*H*)-one-based diarylmethanes **292** and **293** via Pd-catalysed cross-coupling reactions with benzyl indium reagents **291** [118].

Quinolin-2(1*H*)-one-based diaryl methane derivatives **292** and **293** were prepared via basic alumina supported Pd-catalysed cross-coupling of halogenated quinolones **287**–**290** (X = Cl, Br, I) with freshly prepared benzyl indium organometallic reagent **291**, using microwave heating (Scheme 92) [118]. Only 0.5 mol% of Pd(PPh_3_)_4_ with basic alumina could afford high yield of product just in 10 min. Increase of the reaction time did not alter the product yield. No addition of base from outside is required to promote the catalytic reaction in basic alumina, which itself acts as the base during the course of the reaction, whereas neutral alumina or silica gels are very ineffective in absence of base. When the reaction was performed using an oil bath at 120 °C low yield of product was achieved even after overnight heating. Basic alumina can be reused after calcinations at 150 °C for 5 h (after washing with water and acetone) and could be recycled at least 3–4 times with minimum loss on its activity. The procedure offers a broad synthetic route for the construction of new polynuclear heteroaromatic systems having potential biological activity.

## 5. Transformation of Quinolin-4(1*H*)-ones

### 5.1. Heck Reaction

**Scheme 93:**
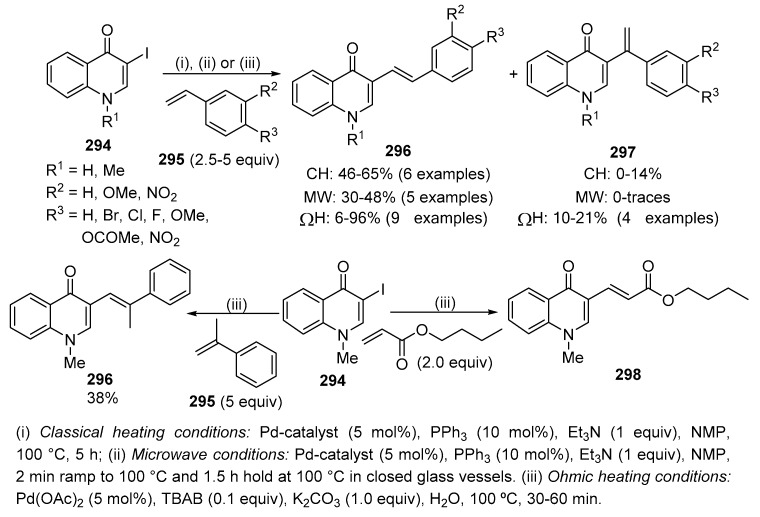
Synthesis of (*E*)-3-substituted-quinolin-4(1*H*)-ones **296**, **297** and **298** by Heck reaction of 3-iodoquinolin-4(1*H*)-ones **294** with styrenes **295** and butyl acrylate, respectively [119,122].

The Heck reaction of 3-iodoquinolin-4(1*H*)-ones **294** with styrene derivatives **295** afforded (*E*)-3-styrylquinolin-4(1*H*)-ones **296** (Scheme 93) [119]. The reaction of 3-iodoquinolin-4(1*H*)-one **294** (R^1^ = H) with styrene afforded the (*E*)-3-styrylquinolin-4(1*H*)-one **296** (R^1^ = R^2^ = R^3^ = H) in moderated yield (46%) using Pd(PPh_3_)_4_ as catalyst, PPh_3_ as ligand and Et_3_N as base in NMP at 100 °C. Change of the Pd source [Pd(OAc)_2_ or Pd/C], the ligand [P(*o*-tol)_3_] [tol = 4-methylphenyl(tolyl)] or the base (NaOAc or K_2_CO_3_) did not improve the yields; instead when P(*o*-tol)_3_ was used, the branched regioisomer 3-(1-phenylethenyl)quinolin-4(1*H*)-one **297** was obtained as the main product (**296**: 10%; **297**: 16%). This Heck procedure revealed to be efficient only when 3-iodoquinolin-4(1*H*)-one was *N*-methylated. The Heck reaction of 3-iodo-1-methylquinolin-4(1*H*)-one **294** led to the (*E*)-1-methyl-3-styrylquinolin-4(1*H*)-one **296** in better yield (55%) although the branched regioisomer **297** was also isolated (14%). When the reaction was performed under MW, shortening of the reaction time occurred but lower yields were obtained (40% in 1.5 h). The best catalyst found for the unsubstituted styrene [Pd(PPh_3_)_4_] did not work well with substituted styrenes, except for styrene **295** [R^2^ = NO_2_; R^3^ = H (CH: 65%; MW: 45%]. For other styrenes **295**, PdCl_2_ proved to be more efficient (R^2^ = H; R^3^ = OMe, CH: 59%, MW: 36%; R^2^ = R^3^ = OMe, CH: 55%, MW: 30%; R^2^ = H; R^3^ = F, CH: 56%; MW: 48%). Later, Silva and co-workers have performed the Heck reaction of 3-iodoquinolin-4(1*H*)-ones **294** with styrene derivatives **295** in an ohmic heating (ΩH) reactor [120,121], in aqueous media, using Pd(OAc)_2_ as catalyst and TBAB as phase transfer catalyst in the presence of an inorganic base [122]. Butyl acrylate was also used as a coupling partner to give a different 3-substituted quinolin-4(1*H*)-one **298** (Scheme 93) [122]. This methodology is environmentally friendly, due to the use of water as solvent and there is no need for costly and toxic phosphine ligands. Yields were dependent on the amount of styrene or acrylate and on the electronic and steric effects of the substituents on the styrene moiety. Moreover, neutral and electron-donating substituents favoured the formation of the branched isomer. In general, yields were moderate to good and in most cases better than those obtained by the conventional procedures that used organic solvents. In addition, ohmic heating proved to be more efficient than conventional and microwave heating.

**Scheme 94:**
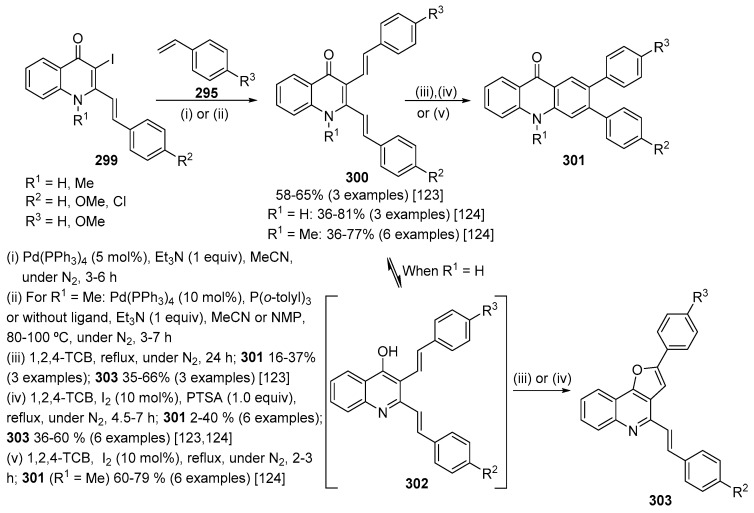
Synthesis of 2,3-diarylacridin-9(10*H*)-ones **301** and (*E*)-2-aryl-4-styrylfuro[3,2-*c*]quinolines **303** via Heck reaction of (*E*)-3-iodo-2-styrylquinolin-4(1*H*)-ones **299** with styrenes **295** [123,124].

The same authors described the Heck reaction of (*E*)-3-iodo-2-styrylquinolin-4(1*H*)-ones **299** with styrenes **295**, leading to (*E*,*E*)-2,3-distyrylquinolin-4(1*H*)-ones **300** in good yields (58–65%) (Scheme 94) [123,124]. Pd(PPh_3_)_4_ was the best catalyst [Pd(OAc)_2_ and PdCl_2_ proved to be unsuccessful], MeCN was the most appropriated solvent and Et_3_N the most suitable base. When heated at high temperatures, the obtained (*E*,*E*)-2,3-distyrylquinolin-4(1*H*)-ones **300** cyclized in two different ways (Scheme 94). Electrocyclization and further in situ oxidation leads to 2,3-diarylacridin-9(10*H*)-ones **301**, whereas tautomerisation, cyclization by nucleophilic attack of the oxygen of the hydroxy group to the β-position of the 3-styryl group and further in situ oxidation produced (*E*)-2-phenyl-4-styrylfuro[3,2-*c*]quinolines **303**. When the reaction was performed in refluxing 1,2,4-trichlorobenzene, acridin-9(10*H*)-ones **301** were obtained in low yields (16–37%) and (*E*)-2-phenyl-4-styrylfuro[3,2-*c*]quinolines **303** as the main products (35–66%). The use of acidic medium, by addition of *p*-toluenesulfonic acid (PTSA), in order to displace the tautomerism of 4-hydroxyquinoline **302** to the quinolone **300** and the addition of iodine to facilitate the isomerization of the double bonds improved the yield of acridin-9(10*H*)-ones **301** (2–40%), although (*E*)-2-phenyl-4-styrylfuro[3,2-*c*]quinolines **303** were still obtained as main products (36–60%) [123,124]. Another method, which gave only 2,3-diaryl-10-methylacridin-9(10*H*)-ones **301** (R^1^ = Me), involves the Heck reaction of *N*-protected (*E*)-3-iodo-1-methyl-2-styrylquinolin-4(1*H*)-ones **299** (R^1^ = Me) and styrenes **295**, leading to (*E*,*E*)-1-methyl-2,3-distyrylquinolin-4(1*H*)-ones **300** (R^1^ = Me), which when heated at high temperatures cyclized through tandem-electrocyclization and oxidation processes affording the expected acridin-9(10*H*)-ones **301** (R^1^ = Me) (Scheme 94)[124].

**Scheme 95:**
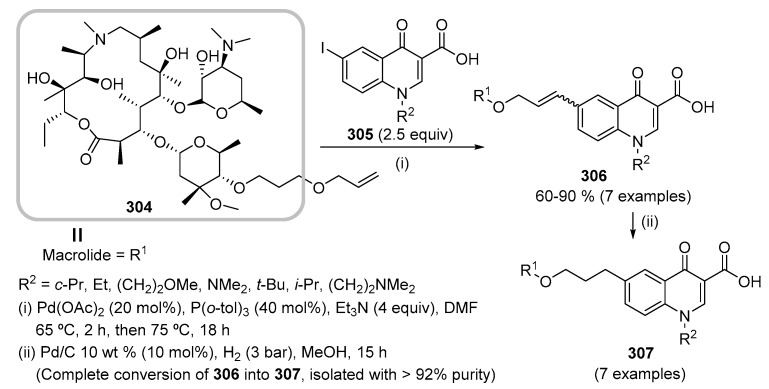
Synthesis of macrolones **307** through Heck reaction of macrolide **304** with 6-iodoquinolin-4(1*H*)-ones **305** followed by Pd/C hydrogenation [125].

The Heck reaction of 6-iodoquinolin-4(1*H*)-ones **305** with macrolides (from azithromycin) **304** allowed the synthesis of compounds with high antibacterial activity [125]. Macrolones (macrolide + quinolone) **306** were isolated in good to excellent yields when using the catalytic system Pd(OAc)_2_/P(*o*-tol)_3_. The optimized protocol required 2.5 mol excess of quinolone in DMF as the solvent and two different temperatures (65 °C and 75 °C). The desirable macrolones **307** were isolated with > 92% purity after complete hydrogenation of **306** catalysed by Pd/C (Scheme 95) [125].

### 5.2. Suzuki-Miyaura Reaction

**Scheme 96:**
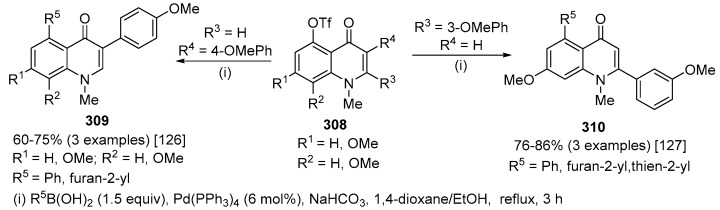
Synthesis of quinolin-4(1*H*)-ones **309** and **310** by Suzuki-Miyaura coupling reaction of 5-trifluoromethanesulfonatequinolin-4(1*H*)-ones **308** with boronic acids [126,127].

3-(4-Methoxyphenyl)-5-trifluoromethanesulfonatequinolin-4(1*H*)-ones **308** were converted into derivatives **309**, in moderate to good yields, by a modified Suzuki methodology (Scheme 96) [126]. This protocol was successfully applied to the synthesis of 2-(3-methoxyphenyl)quinolin-4(1*H*)-one analogues **310** [127].

**Scheme 97:**
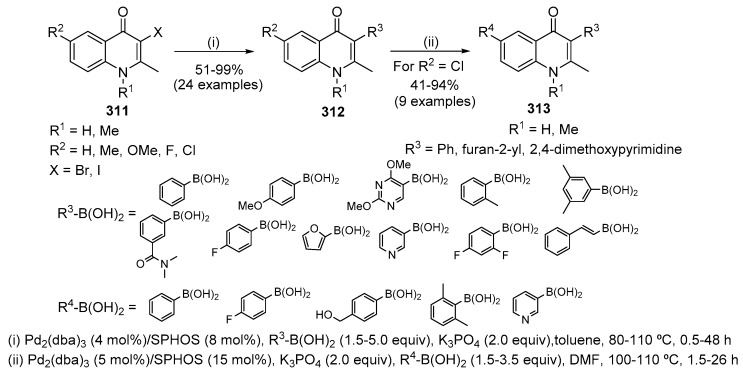
Synthesis of 3-substituted-quinolin-4(1*H*)-ones **312** and their transformation into quinolin-4(1*H*)-ones **313** via Suzuki-Miyaura cross-coupling reactions with boronic acids [128].

Mono- and/or sequential Suzuki-Miyaura cross-coupling reactions of quinolin-4(1*H*)-ones **311** with boronic acids afforded novel medicinally important 3-substituted-quinolin-4(1*H*)-ones **312** (Scheme 97) [128]. When using a Pd/SPHOS catalytic system, the 3-substituted-quinolin-4(1*H*)-ones **312** were obtained in high yields; however, better yields were obtained with 3-iodo- than with 3-bromoquinolin-4(1*H*)-ones. For the less soluble quinolin-4(1*H*)-ones, DMF was required as solvent, while the use of toluene for *N*-methylated quinolone substrates was found to be superior. This reaction was effective for coupling quinolin-4(1*H*)-ones with all major subclasses of substrates including aryl, heteroaryl, both electron-donating and electron-withdrawing, alkenyl, fluorinated and pyridyl, as well as sterically hindered substrates, with moderate to excellent yields.

The divergent nature of this synthetic route was demonstrated using 6-chloro-3-iodoquinolin-4(1*H*)-ones **311**, taking advantage of the reactivity differences between the iodo- and chloro- substituents. Previously, the 3-position was subjected to Suzuki-Miyaura coupling (Scheme 97) and then a subsequent coupling with different boronic acids at 6-position was performed, generating complex quinolin-4(1*H*)-ones **313** (Scheme 97). Very good yields were obtained for 1-methylquinolin-4(1*H*)-ones (70–94%) while the *N*-H containing 6-chloro-3-(2-furanyl)quinolin-4(1*H*)-ones only produced modest yields of the coupling product (41–47%) [128].

**Scheme 98:**
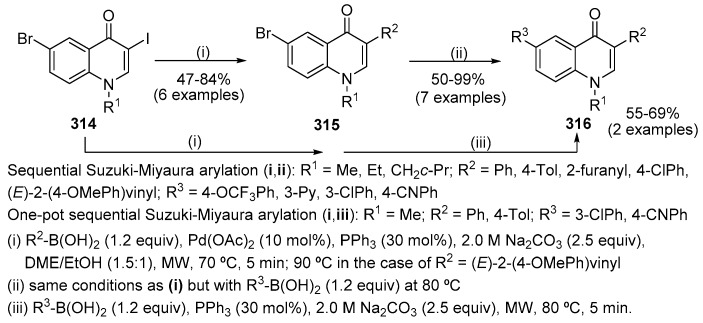
Synthesis of trisubstituted quinolin-4(1*H*)-ones **316** by sequential Suzuki-Miyaura (i, ii) or one-pot double Suzuki-Miyaura (i, iii) reactions of 1-substituted-6-bromo-3-iodoquinolin-4(1*H*)-ones **314** with arylboronic acids [129].

Sequential Suzuki-Miyaura arylation at 3- and 6-position of 1-substituted 6-bromo-3-iodoquinolin-4(1*H*)-ones **314** was carried out regioselectively under standard conditions and controlled reaction temperature [129]. The chemoselective 3-functionalization of the 6-bromo-3-iodoquinolin-4(1*H*)-ones **314** with arylboronic acids afforded 3-aryl-6-bromoquinolin-4(1*H*)-ones **315** in good yields (80–84%), except when 2-furanboronic acid (49%) and 4-methoxyphenylvinylboronic acid (47%) were employed, without concurrent formation of regioisomeric and/or *bis*-coupling products [Scheme 98, (i)]. Further elaboration of compounds **315** into trisubstituted quinolin-4(1*H*)-ones **316** was achieved through sequential Suzuki-Miyaura cross-coupling reaction by exploiting the reactivity of bromine at C-6 position [Scheme 98, (ii)]. The reaction was performed under the same conditions used to generate compounds **315** by increasing the reaction temperature to 80 °C. The trisubstituted quinolin-4(1*H*)-ones **316** were obtained in good yields (67–99%) with complete conversion of the substrates **315** except when using 4-(trifluoromethoxy)phenylboronic acid (50%) [129]. The one-pot sequential Suzuki-Miyaura reaction was also performed to afford the trisubstituted derivatives **316** in overall yields comparable or slightly lower than those obtained in the stepwise synthesis [Scheme 98, (i, ii); 70% for **316** (R^1^ = Me, R^2^ = Ph, R^3^ = 3-ClPh and 72% for **316**: R^1^ = Me, R^2^ = 4-Tol, R^3^ = 3-CNPh)] in a total reaction time of 10 min, without the isolation of the monoarylated intermediate [Scheme 98, (i, iii)] [129].

**Scheme 99:**
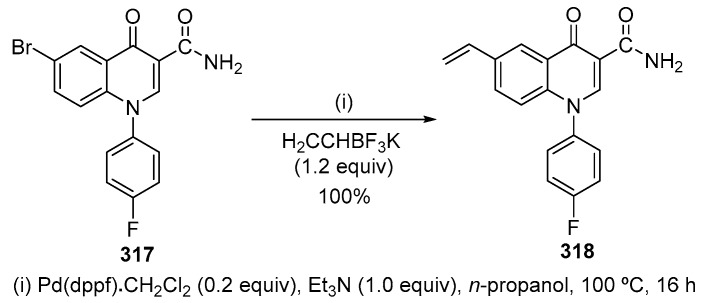
Synthesis of 6-vinylquinolin-4(1*H*)-one **318** by Suzuki-Miyaura coupling of 6-bromoquinolin-4(1*H*)-one **317** with potassium vinyltrifluoroborate [130].

The 6-bromoquinolin-4(1*H*)-one **317** was converted to the corresponding vinyl derivative **318** via a Suzuki coupling with potassium vinyltrifluoroborate (Scheme 99) [130].

**Scheme 100:**
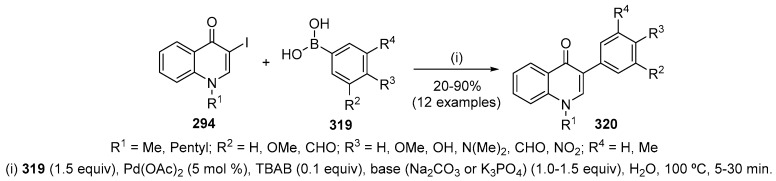
Ohmic heating-assisted synthesis of 3-arylquinolin-4(1*H*)-ones **320** by Suzuki-Miyaura reaction of 1-substituted 3-iodoquinolin-4(1*H*)-ones **294** with arylboronic acids **319** [131].

Potential bioactive 3-arylquinolin-4(1*H*)-ones **320** were synthesized, using ohmic heating, following an efficient and ligand-free protocol for the Suzuki−Miyaura coupling of 1-substituted-3-iodoquinolin-4(1*H*)-ones **294** with several arylboronic acids **319**, in water, using Pd(OAc)_2_ as a catalyst and TBAB as the phase transfer catalyst (Scheme 100) [131]. The reaction is sensitive to the electronic and steric effects of the substituents in the arylboronic acid; in general, arylboronic acids having electron-donating substituents gave better yields. However, the extensive substrate generality, ease of execution and short reaction time make this method exploitable for the generation of libraries of substituted 3-arylquinolin-4(1*H*)-ones **320**. The reaction of **294** (R^1^ = Me) with phenylboronic acid was also performed in classical heating conditions and using microwave irradiation but, for a reaction time of 15 min, ohmic heating was the most efficient heating method [131]. After a simple workup, the Pd/catalyst-H_2_O-TBAB system could be reused for at least seven cycles without significant loss of activity [131].

**Scheme 101:**
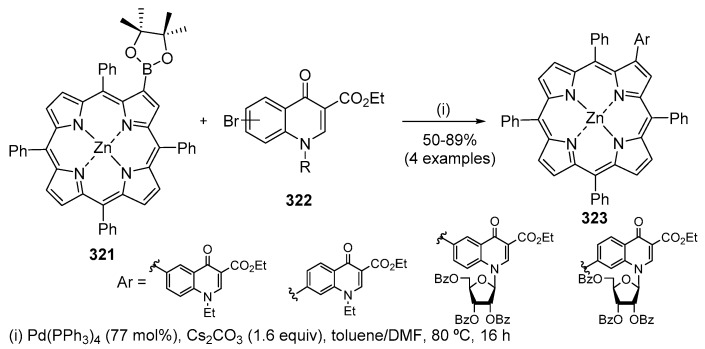
Synthesis of porphyrin-quinolin-4(1*H*)-one conjugates **323** by Suzuki-Miyaura reaction of β-borylated porphyrin **321** with 6- or 7-bromoquinolin-4(1*H*)-ones **322** [132].

Porphyrin-quinolin-4(1*H*)-one conjugates **323** were synthesized by Suzuki-Miyaura coupling reaction of a β-borylated porphyrin **321**, prepared by borylation of the corresponding 3-bromotetraphenylporphyrinatozinc(II) with pinacol borane in the presence of PdCl_2_(PPh_3_)_2_ in dicloroethane at 65 °C for 18 h [132], with 6- or 7-bromoquinolin-4(1*H*)-ones **322** containing *N*-ethyl and *N*-D-ribofuranosyl substituents (Scheme 101) [132]. Quinolones **322** bearing *N*-ethyl substituent were more reactive than those bearing *N*-ribonucleosides providing the corresponding porphyrin-quinolin-4(1*H*)-one conjugates **323** in very good yields (82–89%). The other conjugates **323** were isolated in lower yields (50–51%) but nearly 50% of the starting porphyrin **321** was recovered in both cases. Attempts to improve these yields (e.g., by increasing the reaction time and by increasing the number of equivalents of the bromoquinolin-4(1*H*)-ones) were not successful, probably due to steric effects caused by the ribofuranosyl group. Basic hydrolysis and deprotection of the ribose moieties ester and benzoyl groups in conjugates **323** followed by the acid demetallation afforded the corresponding conjugates that were evaluated as singlet oxygen generators.

**Scheme 102:**
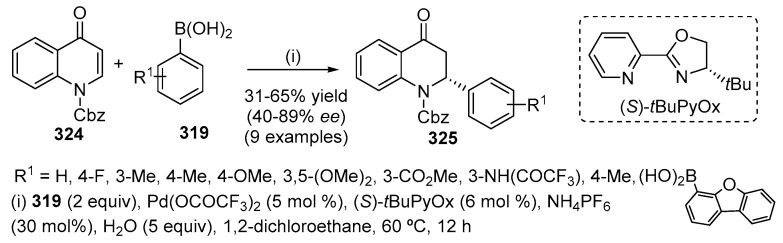
Synthesis of 2-aryl-2,3-dihydroquinolin-4(1*H*)-ones **325** by Pd-catalysed asymmetric conjugate addition of arylboronic acids **319** to quinolin-4(1*H*)-ones **324** [133].

The enantioselective conjugate addition of arylboronic acids **319** to *N*-carboxybenzyl(Cbz)- quinolin-4(1*H*)-ones **324** utilizing the Pd/PyOX catalyst system, under an atmosphere of air, afforded novel 2-aryl-2,3-dihydroquinolin-4(1*H*)-ones **325** with high enantioselectivity in moderate to excellent yields (Scheme 102) [133]. Nitrogen-containing heteroaromatic and simpler boronic acid derivatives were successfully employed as nucleophiles in the 1,4-addition to quinolin-4(1*H*)-ones. Alkyl- and halogen-substituted boronic acids gave reasonable yields (45–65%) and enantioselectivities (67–89% ee) and disubstituted boronic acids were also well tolerated and gave similar results. More sterically demanding boronic acids led to lower product yield.

### 5.3. Sonogashira Reaction

**Scheme 103:**
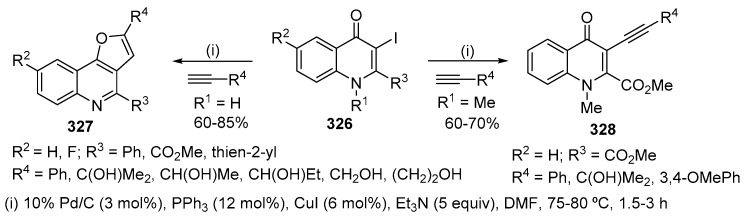
Pd/C-PPh_3_ mediated synthesis of 2-substituted furo[3,2-*c*]quinolines **327** and 3-alkynyl quinolin-4(1*H*)-ones **328** [134].

The tandem coupling-cyclization process, involving Sonogashira reaction followed by the electrophilic or transition-metal-mediated cyclization of the resulting alkynes, having a suitable nucleophilic group in the proximity of the triple bond, generated diverse 2-substituted furo[3,2-*c*]quinolines **327** (Scheme 103) [134,135]. Better yields were obtained when using Pd(PPh_3_)_4_ (85%) or PdCl_2_(PPh_3_)_2_ (80%) instead of Pd/C-PPh_3_ (70%), although the latter is cheaper. DMF was found to be a better solvent when compared to THF or MeCN and CuI is crucial in this reaction; otherwise only de-iodinated product is formed. The absence of PPh_3_ when using Pd/C resulted in a poor yield (22%). Good yields of the furo[3,2-*c*]quinolines **327** were obtained regardless the nature of terminal alkynes used. The key features of the present tandem-coupling-cyclization process are the transition-metal-mediated activation of the triple bond of the 3-alkynyl quinoline generated in situ followed by an intramolecular attack of the oxygen on the activated triple bond with subsequent proton transfer and release of the metal ion to give the desired furoquinolines **327**. The NH of the quinolin-4(1*H*)-one ring has a critical role facilitating the participation of C-4 quinoline oxygen in the cyclization step. Indeed, when 3-iodo-1-methyl-4-oxo-1,4-dihydroquinoline-2-carboxylic acid methyl esters **326** reacted with terminal alkynes, under the same conditions, only 3-alkynylquinolin-4(1*H*)-ones **328** were isolated as a result of a normal Sonogashira coupling reaction and formation of furoquinolines **327** was not observed, even in trace amounts (Scheme 103).

**Scheme 104:**
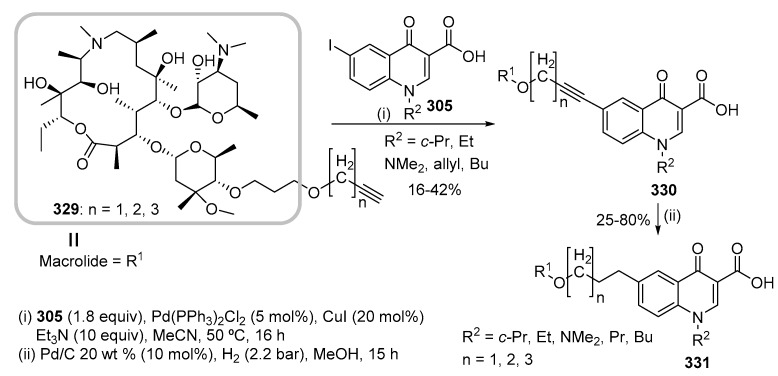
Synthesis of macrolones **331** via Sonogashira reaction of macrolide **329** with 6-iodoquinolin-4(1*H*)-ones **305** followed by Pd/C hydrogenation [125].

Diverse macrolone derivatives, which possess high antibacterial activity, were prepared starting from 6-iodoquinolin-4(1*H*)-ones **305** via Sonogashira reaction [125,136,137]. For example, macrolones **330** were prepared by Sonogashira reaction of 6-iodoquinolin-4(1*H*)-ones **305** with macrolides **329**. Then, Pd/C catalysed hydrogenation was performed to obtain the desirable macrolones **331** (Scheme 104) [125].

**Scheme 105:**
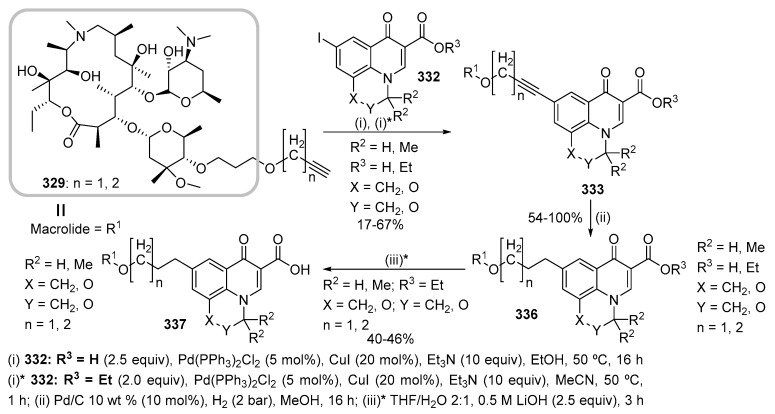
Synthesis of macrolones **336** and **337** via Sonogashira reaction followed by Pd/C hydrogenation and hydrolysis of the corresponding esters **336** [125].

Another group of macrolones (with tricyclic quinolone moiety) **336** (R^3^ = H) and **337** were also synthesized by Sonogashira approach although, only when ethyl esters **332** of the parent acids were used, the reaction proceeded in shorter reaction times and better yields [Scheme 105, (i): R^3^ = H, (i)*: R^3^ = Et]. After hydrogenation [Scheme 105, (ii), esters **336**, R^3^ = Et, were hydrolysed to the corresponding carboxylic acids **337**, Scheme 105, (iii)*] [125].

**Scheme 106:**
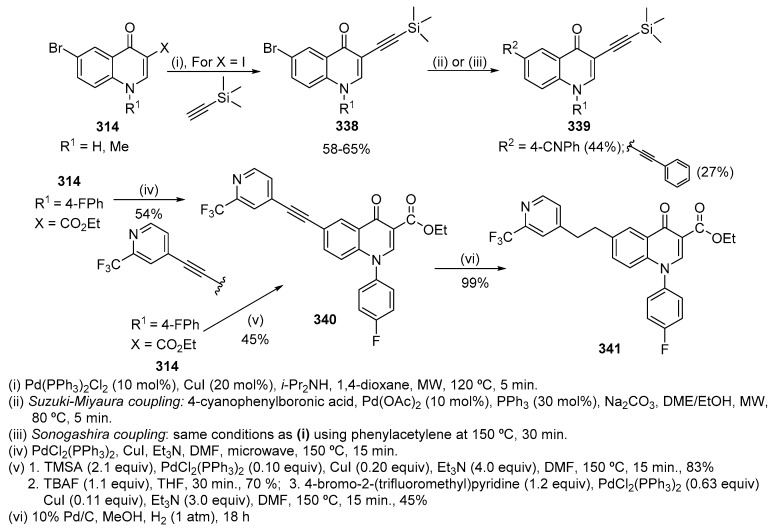
Sonogashira reaction of 6-bromo-3-iodoquinolin-4(1*H*)-ones **314** and subsequent Suzuki and Sonogashira reactions at 6-position of quinolin-4(1*H*)-ones **338** [129,130].

The Sonogashira reaction of 6-bromo-3-iodoquinolin-4(1*H*)-ones **314** with TMSA afforded the expected coupling products in very satisfactory yields, with no further conversion to furo[3,2-*c*]quinoline derivatives (Scheme 106) [129]. Suzuki and Sonogashira cross-coupling reactions at 6-position of substrates **338** yielded the trisubstituted quinolin-4(1*H*)-ones **339**. In general, harder conditions were necessary to functionalize the 6-position due to the lower reactivity of bromine compared to iodine but, on the other hand, the increased structural complexity made substrates **338** more prone to decomposition and side products’ formation. Therefore, cross-coupling reactions at 6-position proceeded with slightly lower efficiency, leading to products **339** in moderate to low yields.

Another 6-bromoquinolin-4(1*H*)-one **314** (R^1^ = 4-FPh, X = CO_2_Et) was converted to the corresponding alkyne derivatives via Sonogashira couplings; the coupling of **314** with a terminal alkyne (R^2^CCH) gave **340** directly, whereas, the coupling with trimethylsilylacetylene followed by fluoride mediated silyl cleavage gave a 6-alkyne intermediate that was coupled to an aryl bromide (R^2^Br) to afford **340** (Scheme 106) [130]. The Pd-catalysed hydrogenation of the alkyne moiety of **340** provided derivative **341** [130].

**Scheme 107:**
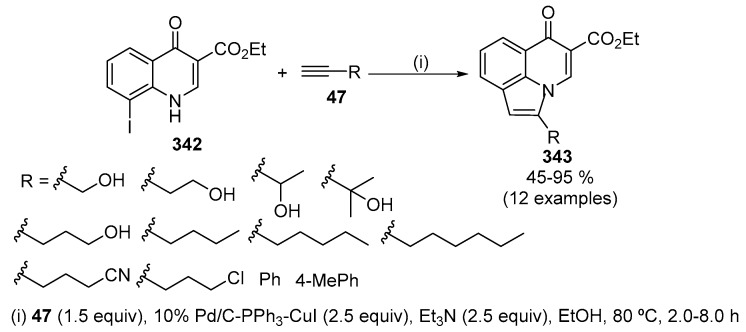
Synthesis of 2-substituted 6-oxopyrrolo[3,2,1-*ij*]quinolines **343** by Pd/C-mediated coupling-cyclization of 8-iodo-4-oxo-1,4-dihydroquinoline-3-carboxylic acid ethyl ester **342** with terminal alkynes **47** [138].

A practical synthesis of 2-substituted 6-oxopyrrolo[3,2,1-*ij*]quinolines **343** was achieved following a single-step Pd/C-mediated coupling-cyclization strategy. The methodology involves the reaction of 8-iodo-4-oxo-1,4-dihydroquinoline-3-carboxylic acid ethyl ester **342** with a variety of terminal alkynes **47** in the presence of 10% Pd/C–PPh_3_–CuI as a catalyst system in EtOH (Scheme 107) [138]. This process was found to be general since both aliphatic and aromatic alkynes are tolerated, as well as alkyl side chains containing primary, secondary or tertiary hydroxyl group or a cyano group.

### 5.4. Stille Reaction

**Scheme 108:**
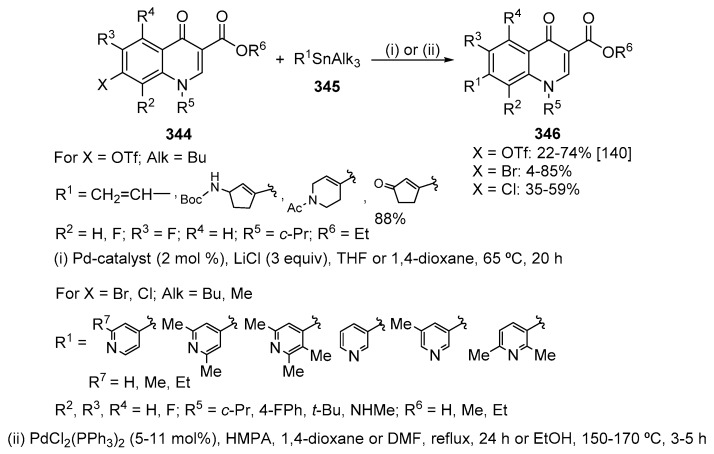
Pd-catalysed Stille cross-coupling reaction of 7-quinolyltriflates **344** with functionalized stannanes **345** [139, 140, 141].

A series of 1-cyclopropylquinolin-4(1*H*)-ones **346** (R^5^ = *c*-Pr), bearing a vinyl, a 1-cyclopentenyl or a 1,2,3,6-tetrahydropyridin-4-yl group at C-7, were synthesized by Pd-catalysed cross-coupling of 7-quinolyltriflate **344** (X = OTf) with an appropriately functionalized vinylstannane **345** (1.2 equiv) [Scheme 108, (i)] [139,140]. The reaction with a range of vinylstannanes **345** bearing different functional groups afforded the corresponding quinolin-4(1*H*)-ones **346** in moderate to good yields (22–88%) with complete chemo- and regioselectivity; the coupling takes place exclusively at the C-7 position of the quinolone nucleus even in the presence of the C-6 fluorine or the C-2 α,β-unsaturated keto-ester moiety. In some cases, additional amounts of the tin reagent and/or the catalyst [either PdCl_2_(PPh_3_)_2_ or Pd(PPh_3_)_4_] were necessary to improve the yield of coupled product. The low yield obtained with the cyclopentenylstannane possessing a bulky *t*-butoxycarbonyl group at the 3-position (31%) was attributed to unfavourable steric hindrance. Later, novel 7-pyridinylquinolin-4(1*H*)-ones (**346**, R^1^ = pyridinyl group) were synthesized, following the same methodology but starting from 7-bromo- or 7-chloroquinolin-4(1*H*)-ones **344** (X=Br or Cl), [Scheme 108, (ii)] [141]. Likewise, the quinolin-4(1*H*)-one esters **346** were hydrolysed to the corresponding acids (50–73%), with sodium hydroxide or hydrochloric acid, with concomitant removal of the *N*-protecting groups.

**Scheme 109:**
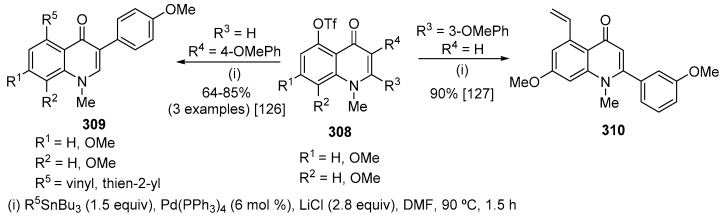
Synthesis of quinolin-4(1*H*)-ones **309** and **310** by Stille coupling reaction between 5-trifluoromethanesulfonatequinolin-4(1*H*)-ones **308** and tributyl(vinyl)stannanes [126,127].

5-Substituted-3-(4-methoxyphenyl)quinolin-4(1*H*)-ones **309** have been synthesized in good yields via Stille cross-coupling reaction of the corresponding 3-(4-methoxyphenyl)-5-trifluoromethanesulfonatequinolin-4(1*H*)-ones **308** with tributyl(vinyl)stannane (67–85%) or with tributyl(thien-2-yl)stannane (64%) (Scheme 109) [126]. This protocol was successfully applied to the synthesis of 2-(3-methoxyphenyl)quinolin-4(1*H*)-one analogue **310** [127].

### 5.5. Buchwald-Hartwig Reaction

**Scheme 110:**
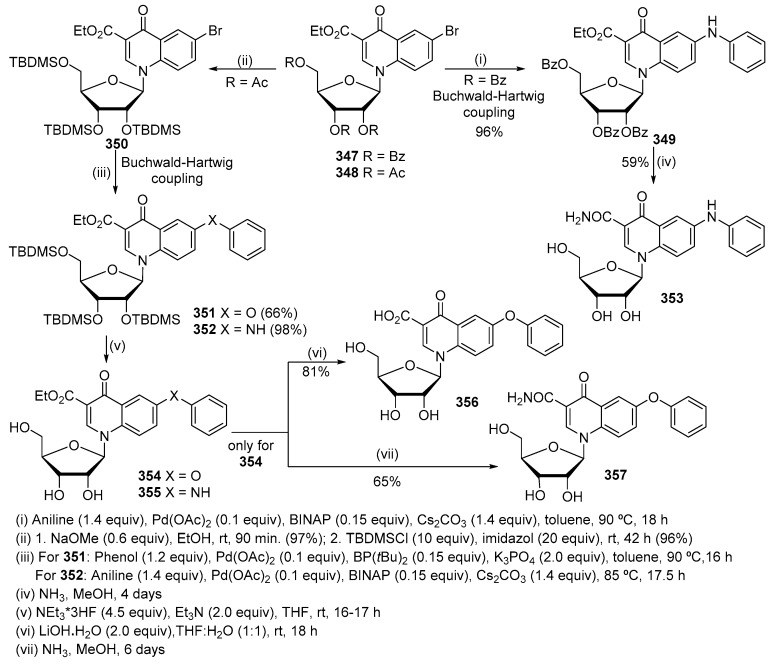
Synthesis of 6-substituted-quinolin-4(1*H*)-one nucleosides by Pd-catalysed Buchwald-Hartwig cross-coupling reactions of 6-bromoquinolin-4(1*H*)-one nucleosides **347** and **348** with aniline and phenol [142].

Four 6-substituted-quinolin-4(1*H*)-one nucleosides linked to aniline or phenol via *N*- or heteroatom-bridges were synthesized by Pd-catalysed Buchwald-Hartwig cross-coupling reactions starting from 6-bromoquinolin-4(1*H*)-one nucleosides **347** and **348**. Different standard deprotection steps resulted in free ribose for all compounds **353**–**357** and either the free acid **356**, the amides **353** and **357** or the esters **354** and **355** in the 3-position of quinolin-4(1*H*)-one (Scheme 110) [142].

**Scheme 111:**
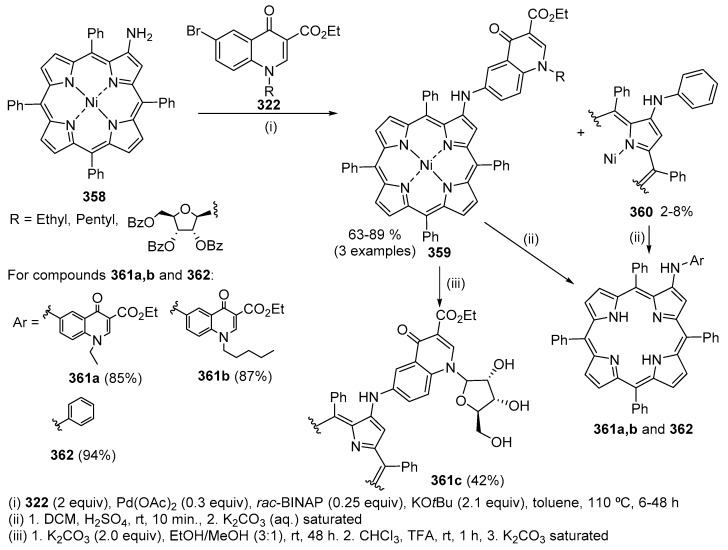
Synthesis of porphyrin-quinolin-4(1*H*)-one conjugates **361** and **362** by Pd-catalysed amination of 6-bromoquinolin-4(1*H*)-ones **322** with (2-amino-5,10,15,20-tetraphenylporphyrinato) nickel(II) **358** followed by demetallation [143].

Pd-catalysed amination reactions of 6-bromoquinolin-4(1*H*)-ones **322** containing *N*-ethyl, *N*-pentyl and *N*-ribofuranosyl substituents with (2-amino-5,10,15,20-tetraphenylporphyrinato) nickel(II) **358** followed by demetallation, allowed the access to porphyrin-quinolin-4(1*H*)-one conjugates **361** and **362** (Scheme 111) [143]. The use of Pd(OAc)_2_ in combination with *rac*-BINAP, as the ligand, led to complete consumption of starting material **322** and to the formation of the conjugates **359** in moderate to very good yields (63–89%). The yields were lower and longer reaction time was required in the case of quinolones having bulky groups, such as pentyl and ribofuranosyl groups, probably due to steric effects. The formation of the by-products **360** is promoted by the transfer of a *rac*-BINAP phenyl group to the metal ion centre followed by a reductive-elimination step during the catalytic cycle. The conjugates **361a** demonstrated good to high capability to generate singlet oxygen evidencing potential application in the inactivation of the Gram-positive bacteria *Staphylococcus aureus* [143].

**Scheme 112:**
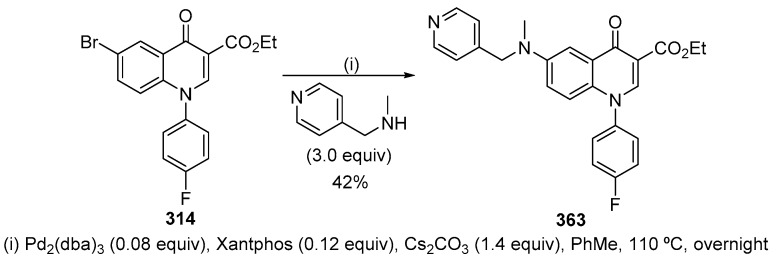
Synthesis of quinolin-4(1*H*)-one **363** by Pd-catalysed amination of 6-bromoquinolin-4(1*H*)-one **314** [130].

Buchwald-Hartwig amination of 6-bromoquinolin-4(1*H*)-one **314** with a commercially available amine afforded quinolin-2(1*H*)-one **363** in 42% yield (Scheme 112) [130].

### 5.6. Aminocarbonylation Reaction

**Scheme 113:**
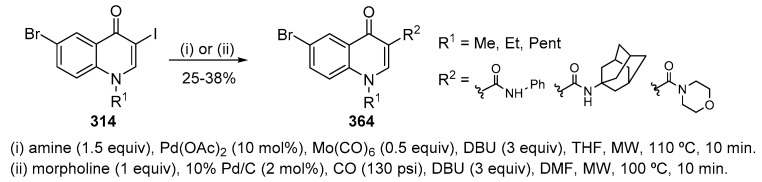
Pd-catalysed aminocarbonylation of 6-bromo-3-iodoquinolin-4(1*H*)-ones **314** [129].

A Pd-catalysed carbonylation of 6-bromo-3-iodoquinolin-4(1*H*)-ones **314** with amines, using Pd(OAc)_2_ as catalyst and Mo(CO)_6_ as the CO source, provided compounds **364** in low yields (25–38%) [Scheme 113, (i)] [129]. Slight modifications of the experimental conditions did not improve the reaction yield; even when gaseous CO and Pd/C were used as the catalyst [Scheme 113, (ii)] the aminocarbonylation product was obtained in similar yield (35%).

### 5.7. Other Reactions

**Scheme 114:**
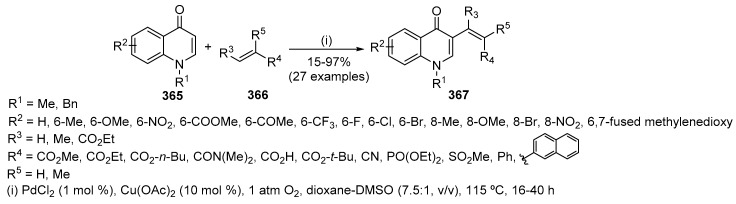
Direct 3-alkenylation of quinolin-4(1*H*)-ones **365** [144].

Pd-catalysed 3-alkenylation of quinolin-4(1*H*)-ones **365** with compounds **366** was efficiently achieved with 1% catalyst loading affording 3-substituted-quinolin-4(1*H*)-ones **367** (Scheme 114) [144]. From a series of tested catalysts [Pd(OAc)_2_, PdCl_2_, Pd(TFA)_2_], PdCl_2_ was the most efficient at low loadings. The addition of Cu(OAc) (10 mol%) was necessary to achieve high yield of the product. The presence of O_2_ in the reaction medium, which acts as a co-oxidant, is favourable. Regarding the reaction scope, a substituent is required on the nitrogen; coupling of free (NH) quinolone with acrylate gave no product. The presence of electron-donating groups at the C-6 position provided high yields while electron-withdrawing groups gave lower yields; however, high yield was obtained when CF_3_ group was at that position and with halogens (6-F, 6-Cl and 6-Br). 8-Substituted quinolones have a similar reactivity as 6-substituted ones but generally with lower yields, probably due to steric effects. Even the 6,7-(methylenedioxy)-substituted quinolone gave the product in modest yield. Terminal acrylates, *N*,*N*-dimethylacrylamide, styrene and 2-vinylnaphthalene gave the corresponding products in high yields while modest to reasonable yields were obtained with acrylic acid, sterically hindered acrylates, diethyl fumarate, methacrylonitrile, vinyl phosphonate and methyl vinyl sulfone.

**Scheme 115:**
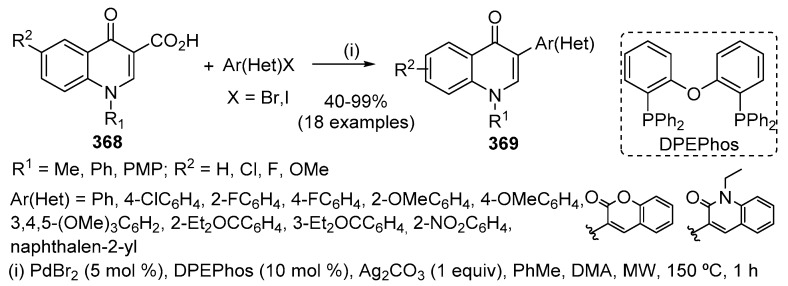
Metal-catalysed decarboxylative coupling of quinolin-4(1*H*)-one 3-carboxylic acids **368** with (hetero)-aryl halides [145].

An efficient and practical metal-catalysed decarboxylative cross-coupling reaction of quinolin-4(1*H*)-one 3-carboxylic acids **368** with various (hetero)-aryl halides was described (Scheme 115) [145]. An extensive screening of various reaction parameters (Pd, ligand, solvent, base and temperature) showed that PdI_2_, Pd(OAc)_2_ and PdCl_2_ were less effective than PdBr_2_. The nature of the phosphine ligand has an important influence on the reaction selectivity and optimal reaction conditions of **368** (R^1^ = Ph; R^2^ = H) with 4-iodoanisole involved the combination of the bidentate phosphine DPEphos with PdBr_2_ in toluene/dimethylacetamide (DMA) at 150 °C. The use of microwave irradiation provided shortening of reaction time and increase of quinolin-4(1*H*)-ones **369** yield (MW: 1 h, 81%; CH: 8 h, 77% and 1 h, 60%). The bimetallic system PdBr_2_/Ag_2_CO_3_ is necessary for the coupling to occur; no product could be formed in the absence of PdBr_2_ or when Ag_2_CO_3_ was replaced by other bases. Using optimal conditions under microwave irradiation, electron-rich and electron-deficient, *o*-, *m*- and *p*-substituted aryl iodides and bromides, all efficiently underwent decarboxylative coupling in good yields (40–99%) and the coupling with heterocyclic halides was also successful (40% for 3-bromocoumarin and 57% for 3-bromoquinolin-2(1*H*)-one). Both *N*-alkyl- and *N*-arylquinolin-4(1*H*)-one 3-carboxylic acids **368** having electron-donating or electron-withdrawing groups on the aromatic nucleus led to the formation of the corresponding coupled products **369** in good yields (60–90%). Excellent chemical selectivity was observed for **368** (R^2^ = Cl) preserving the C-Cl bond, which could undergo further metal-catalysed functionalization reactions. This protocol is an attractive alternative to the existing methods for the synthesis of 3-(hetero)-arylquinolin-4(1*H*)-ones **369** and was also applied to the synthesis of 1-methyl-3-phenylquinolin-2(1*H*)-one (44%).

**Scheme 116:**
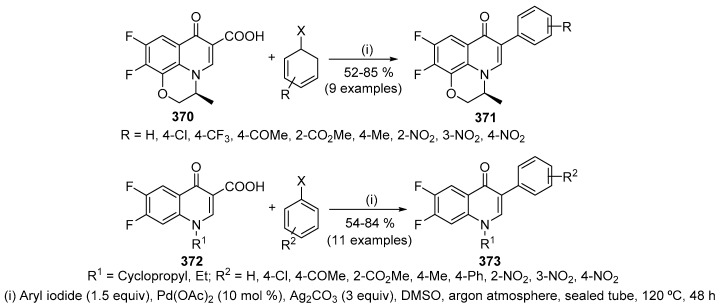
Synthesis of 3-arylquinolin-4(1*H*)-ones **371** and **373** by Pd-catalysed decarboxylative cross-coupling of quinolin-4(1*H*)-one 3-carboxylic acids **370** and **372** with aryl halides [146].

The ligand-free palladium-catalysed decarboxylative functionalization of quinolinone-3-carboxylic acids **370** and **372** with aryl halides afforded biologically important 3-arylquinolin-4(1*H*)-ones **371** and **373** in moderate to high yields (Scheme 116) [146]. The reaction proceeded smoothly under an argon atmosphere at relatively low temperature by using Pd(OAc)_2_ as the catalyst and Ag_2_CO_3_ as the oxidant. Aryl iodides gave better results than aryl bromides and various functionalities were compatible under the reaction conditions. Electronic effects strongly influenced the reaction. The coupling of aryl iodides bearing electron-donating substituents provided the corresponding products in high yields but iodobenzene derivatives with electron-deficient groups were significantly less reactive.

**Scheme 117:**
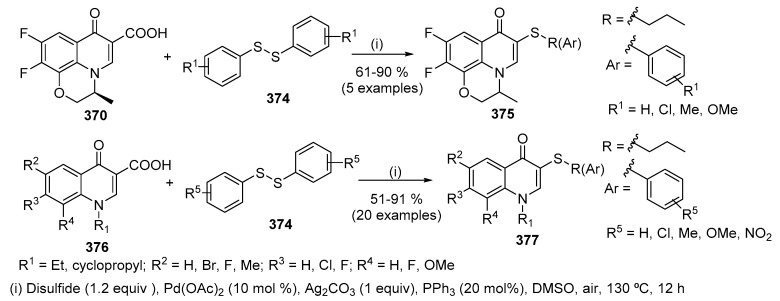
Pd-catalysed direct thioetherification of quinolin-4(1*H*)-one-3-carboxylic acids **370** and **376** [147].

Pd-catalysed direct thioetherification of quinolone derivatives **370** and **376** with diaryl disulfides **374** via decarboxylative C-S cross-couplings proceeded smoothly under air in the presence of Pd(OAc)_2_ and Ag_2_CO_3_ in DMSO (Scheme 117) [147]. The solvent has an important role in the reaction, DMSO being superior to other solvents and no product was isolated when the reaction was performed under argon or nitrogen atmosphere. Disulfides **374** substituted with electron-withdrawing groups gave the product in lower yields. In addition, when some radical scavengers such as TEMPO or HQ were used in this C-S coupling, the reaction was inhibited and no product was detected. The reaction mechanism initiates with the formation of an organometallic species via decarboxylation by reaction with the silver salt. Subsequently, the Pd(II) reacts with the diaryl disulfides to generate a Pd(II) species. Then, an aryl-Pd(II) species was produced by way of a transmetalation reaction between the organometallic and Pd(II) species. Finally, the reductive elimination of the aryl Pd(II) afforded the target product and Pd(0) species, which can be oxidized to Pd(II) by oxygen and continue the catalytic cycle [147]. This protocol is an alternative to existing approaches to construct aryl sulphides of quinolone derivatives which may be used as important intermediates in the synthesis of new drug candidates.

**Scheme 118:**
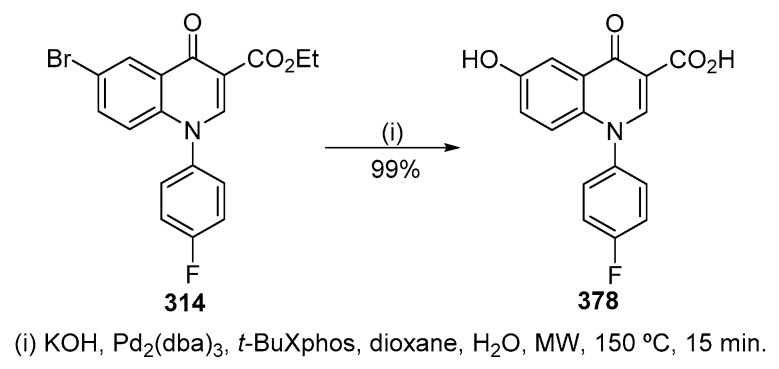
Synthesis of 6-hydroxyquinolin-4(1*H*)-one **378** by Pd-catalysed hydroxylation [130].

Pd-catalysed hydroxylation of 6-bromoquinolin-4(1*H*)-one **314** allowed the synthesis of quinolin-4(1*H*)-one **378** with a 6-hydroxy group (Scheme 118) [130].

**Scheme 119:**
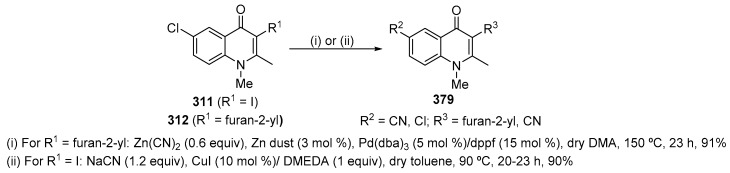
Cyanation of 6-chloro-1-methylquinolin-4(1*H*)-ones **311** and **312** [128].

6-Chloroquinolin-4(1*H*)-one **312** was efficiently converted into 3-furanyl-6-cyanoquinolin-4(1*H*)-one **379**, in high yields, using a Pd/dppf catalyst with Zn(0) as the in situ reductant (Scheme 119) [128]. The same conditions failed to catalyse the cyanation of 6-chloro-3-iodoquinolin-4(1*H*)-one **311**. However, using a copper iodide/DMEDA system with sodium cyanide resulted in 3-cyanation with excellent yield (Scheme 119) [128].

**Scheme 120:**
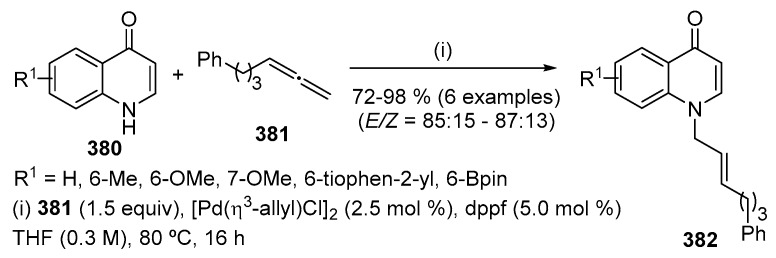
Synthesis of *N*-allylated quinolin-4(1*H*)-ones **382** by Pd-catalysed addition of quinolin-4(1*H*)-ones **380** to terminal allenes **381** [148].

Pd-catalysed addition of quinolin-4(1*H*)-ones **380** to terminal allenes **381**, using an achiral Pd-catalyst, furnished linear *N*-allylated quinolin-4(1*H*)-ones **382** in high yields and high (*E*)-selectivity (Scheme 120) [148]. Compatible functional groups on the quinolin-4(1*H*)-ones were methyl, methoxy, a thiophene and a boronic ester, enabling further functionalization through cross-coupling reactions.

**Scheme 121:**
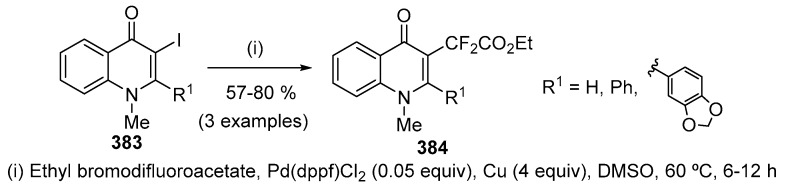
Synthesis of difluoro-containing quinolin-4(1*H*)-ones **384** by Pd-catalysed cross-coupling reaction of 3-iodo-quinolin-4(1*H*)-one **383** with ethyl bromodifluoroacetate [149].

**Scheme 122:**
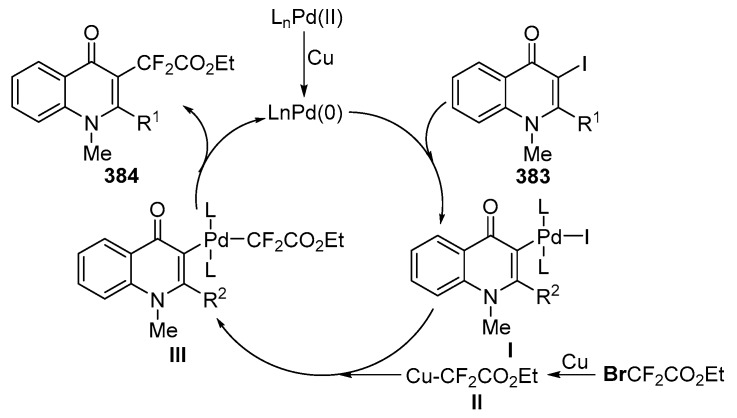
Mechanism of the copper-mediated Pd-catalysed cross-coupling reaction [149].

Quinolin-4(1*H*)-ones **383** undergo Pd-catalysed cross-coupling reaction with ethyl bromodifluoroacetate, in the presence of copper mediators, providing difluoro-containing quinolin-4(1*H*)-ones **384** (Scheme 121) [149]. Among the tested Pd-catalysts, Pd(dppf)Cl_2_ provided compounds **384** in less time and higher yields. Solvent has a significant impact on the yields with DMSO giving the best result. This method was employed in the modification of graveolinine, a natural quinolin-4(1*H*)-one alkaloid with interesting antibacterial, spasmolysis and antitumor activities. Treatment of 3-iodograveolinine (**383**, R^1^ = benzo[*d*][1,3]dioxole) with ethyl bromodifluoroacetate gave the target compound in 78% yield. Since a Pd(II) catalyst is employed in this reaction, first it may be reduced by copper to an active Pd(0) species, which then reacts via an oxidative addition into the C−I bond of compound **383** to form intermediate I. Meanwhile, the unstable copper ethyl difluoroacetate complex II is formed and intermediate III is generated rapidly via a reaction between intermediates I and II. Finally, reduction elimination affords the expected quinolin-4(1*H*)-one **384**, regenerating the Pd(0) catalyst (Scheme 122) [149].

## 6. Conclusions

Pd-catalysed reactions are of paramount importance in the synthesis and transformation of quinolin-2(1*H*)-ones and quinolin-4(1*H*)-ones, as it was evidenced by the several examples presented along these review article. In some of these examples, especially in the synthesis of quinolones, the claimed Pd-catalysed reaction is crucial for the formation of the appropriate substrate for cyclization into the desired quinolone, in spite of not being used in the cyclization step.

Among the Pd-catalysed reactions developed for the synthesis and transformation of quinolones, the cross-coupling reactions, which require the use of an already activated counterpart, for instance a halogenated derivative, which is coupled to another appropriate substrate, are the most common. More recently, several works have been focused on the development of protocols for direct C-H functionalization, thus allowing the construction of more complex and highly substituted quinolone derivatives in a more straightforward way. In addition, efforts have been made to develop new protocols for already known Pd-catalysed reactions, aiming to meet the green chemistry requirements and to facilitate the reactions′ scale-up. Some important advances have been achieved in this area by replacing homogeneous Pd-catalysts by heterogeneous Pd-catalysts, by using ligand-free conditions or room temperature. Another interesting example, in the carbonylation reactions, is the replacement of gaseous CO by more convenient solid sources of CO, such as molybdenum hexacarbonyl [Mo(CO)_6_], facilitating the reactions’ scale-up. All the Pd-catalysed reactions herein presented have profoundly changed the protocols for the construction of various quinolone-type compounds, some of them of recognized importance due to their relevant biological activities. In spite of the great advances of Pd-catalysed reactions achieved in the last 20 years, this field of research is still wide-open for innovation and will continue to advance as even more versatile transformations are developed.

## References

[1] Michael J.P. (1991). Quinoline, quinazoline and acridone alkaloids. Nat. Prod. Rep..

[2] Michael J.P. (2008). Quinoline, quinazoline and acridone alkaloids. Nat. Prod. Rep..

[3] Uchida M., Tabusa F., Komatsu M., Morita S., Kanbe T., Nakagawa K. (1987). Studies on 2(1*H*)-quinolinone derivatives as gastric antiulcer active agents. Synthesis and antiulcer activities of optically active alpha-amino acid derivatives of 2(1*H*)-quinolinone and oxindole. Chem. Pharm. Bull..

[4] Norman P. (2002). Tipifarnib (Janssen Pharmaceutica). Curr. Opin. Investig. Drugs.

[5] Richter S., Parolin C., Palumbo M., Palù G. (2004). Antiviral properties of quinolone-based drugs. Curr. Drug Targets Infect. Disord..

[6] Hopkins A.L., Ren J., Milton J., Hazen R.J., Chan J.H., Stuart D.I., Stammers D.K. (2004). Design of non-nucleoside inhibitors of HIV-1 reverse transcriptase with improved drug resistance properties. 1. J. Med. Chem..

[7] Freeman G.A., Andrews C.W., Hopkins A.L., Lowell G.S., Schaller L.T., Cowan J.R., Gonzales S.S., Koszalka G.W., Hazen R.J., Boone L.R. (2004). Design of non-nucleoside inhibitors of HIV-1 reverse transcriptase with improved drug resistance properties. 2. J. Med. Chem..

[8] Huse H., Whiteley M. (2011). 4-Quinolones: Smart phones of the microbial world. Chem. Rev..

[9] Winter R., Kelly J.X., Smilkstein M.J., Hinrichs D., Koop D.R., Riscoe M.K. (2011). Optimization of endochin-like quinolones for antimalarial activity. Exp. Parasitol..

[10] Xia Y., Yang Z.Y., Xia P., Bastow K.F., Nakanishi Y., Nampoothiri P., Hamel E., Brossi A., Lee K.H. (2003). Antitumor agents. Part 226: Synthesis and cytotoxicity of 2-phenyl-4-quinolone acetic acids and their esters. Bioorg. Med. Chem. Lett..

[11] Wang S.W., Pan S.L., Huang Y.C., Guh J.H., Chiang P.C., Huang D.Y., Kuo S.C., Lee K.H., Teng C.M. (2008). CHM-1, a novel synthetic quinolone with potent and selective antimitotic antitumor activity against human hepatocellular carcinoma in vitro and in vivo. Mol. Cancer Ther..

[12] Joule J.A., Mills K. (2000). Quinolines and isoquinolines: Reaction and synthesis. Heterocyclic chemistry.

[13] Fuson R.C., Burness D.N. (1946). A new synthesis of 2-aryl-4-hydroxyquinolines. J. Am. Chem. Soc..

[14] Boteva A.A., Krasnykh O.P. (2009). The methods of synthesis, modification and biological activity of 4-quinolones (review). Chem. Heterocycl. Comp..

[15] Seixas R.S.G.R., Silva V.L.M., Silva A.M.S. (2016). Metal-catalysed cross-coupling reactions in the synthesis and transformations of quinolones and acridones, in synthesis and modification of heterocycles by metal-catalyzed cross-coupling reactions. Top. Heterocycl. Chem..

[16] Cortese N.A., Ziegler C.B.J., Hrnjez B.J., Heck R.F. (1978). Palladium-catalyzed synthesis of 2-quinolones derivatives from 2-iodoanilines. J. Org. Chem..

[17] Bernini R., Cacchi S., Fabrizi G., Sferrazza A. (2006). 4-Aryl-2-quinolones via a domino Heck reaction/cyclization process. Heterocycles.

[18] Bernini R., Cacchi S., De Salve I., Fabrizi G. (2006). The Heck reaction of β-arylacrylamides: An approach to 4-aryl-2-quinolones. Synlett.

[19] Battistuzzi G., Bernini R., Cacchi S., De Salve I., Fabrizi G. (2007). 4-Aryl-2-quinolones through a pseudo-domino Heck/Buchwald–Hartwig reaction in a molten tetrabutylammonium acetate/tetrabutylammonium bromide mixture. Adv. Synth. Catal..

[20] Fourquez J.M., Godard A., Marsais F., Quéquiner G. (1995). Regioselectivity of the metalation of polymethoxylated pivaloylaminobenzenes. Synthesis of methoxy-2(1*H*)-quinolones. J. Heterocycl. Chem..

[21] Cho C.S., Kim J.U. (2007). An approach for quinolines via palladium-catalyzed Heck coupling followed by cyclization. Tetrahedron Lett..

[22] Taylor J.G., Correia C.R.D. (2011). Stereoselective synthesis of unsymmetrical β,β-diarylacrylates by a Heck-Matsuda reaction: Versatile building blocks for asymmetric synthesis of β,β-diphenylpropanoates, 3-aryl-indole and 4-aryl-3,4-dihydro-quinolin-2-one and formal synthesis of (−)-indatraline. J. Org. Chem..

[23] Felpin F.X., Coste J., Zakri C., Fouquet E. (2009). Preparation of 2-quinolones by Heck reduction-cyclization (HRC) reactions by using a multitask palladium catalyst. Chem. Eur. J..

[24] Djakovitch L., Batail N., Genelot M. (2011). Recent advances in the synthesis of *N*-containing heteroaromatics via heterogeneously transition metal catalysed cross-coupling reactions. Molecules.

[25] Glover B., Harvey K.A., Liu B., Sharp M.J., Tymoschenko M.F. (2003). Regioseletive palladium-catalyzed arylation of 3-carboalkoxy furan and thiophene. Org. Lett..

[26] Borhade S.R., Waghmode S.B. (2011). An efficient synthesis of 4-arylquinolin-2(1*H*)-ones and 3-alkenyl-4-arylquinolin-2(1*H*)-one using a Pd/NiFe_2_O_4_-catalyzed consecutive Heck reaction. Can. J. Chem..

[27] Kuroda T., Suzuki F. (1991). Synthesis of 1H-imidazo[4,5-c]quinolin-4(5*H*)-one via palladium-catalyzed cyclization of *N*-(2-bromophenyl)-1*H*-imidazole-4-carboxamide. Tetrahedron Lett..

[28] Beccalli E.M., Broggini G., Martinelli M., Paladino G., Zoni C. (2005). Synthesis of tricyclic quinolones and naphthyridones by intramolecular Heck cyclization of functionalized electron-rich heterocycles. Eur. J. Org. Chem..

[29] Liu Z., Shi C., Chen Y. (2008). Synthesis of 3-alkyl-1H-quinolin-2-ones via palladium-catalyzed intramolecular cyclization of benzyl halides and α,β-unsaturated amides. Synlett.

[30] Arcadi A., Cacchi S., Fabrizi G., Manna F., Pace P. (1998). Ethyl *N*-(o-ethynyl)malonanilide as a useful building block for the preparation of 3,4-disubstituted-2(1*H*)-quinolones, 3,4-disubstituted- and 2,3,4-trisubstituted quinolones. Synlett.

[31] Kadnikov D.V., Larock R.C. (2003). Palladium-catalyzed carbonylative annulation of terminal alkynes: Synthesis of coumarins and 2-quinolones. J. Organomet. Chem..

[32] Torii S., Okumoto H., Xu L.H. (1991). Palladium-catalyzed carbonylation to form 2-substituted 1,4-dihydro-4-oxo-quinoline. Tetrahedron Lett..

[33] Torii S., Okumoto H., Xu L.H., Sadakane M., Shostakovsky M.V., Ponomaryov A.B., Kalinin V.N. (1993). Synthesis of chromones and quinolones via Pd-catalyzed carbonylation of o-iodophenols and anilines in the presence of acetylenes. Tetrahedron.

[34] Kadnikov D.V., Larock R.C. (2004). Synthesis of 2-quinolones via palladium-catalyzed carbonylative annulation of internal alkynes by *N*-substituted *o*-iodoanilines. J. Org. Chem..

[35] Chen J.-R., Liao J., Xiao W.-J. (2010). Microwave-assisted, palladium-catalyzed carbonylative cyclization—Rapid synthesis of 2-quinolones from unprotected 2-iodoanilines and terminal alkynes. Can. J. Chem..

[36] Jafarpour F., Otaredi-Kashani A. (2014). Carbonylative annulation of unsaturated compounds using molybdenum hexacarbonyl: An efficient synthesis of 2(1*H*)-quinolones. Arkivoc.

[37] Vicente J., Abad J.-A., López-Serrano J., Jones P.G., Nájera C., Botella-Segura L. (2005). Synthesis and reactivity of ortho-palladated arylureas. Synthesis and catalytic activity of a C,N,C pincer complex. Stoichiometric syntheses of some N-heterocycles. Organometallics.

[38] Zhang X., Liu H., Jia Y. (2016). A palladium-catalyzed intramolecular carbonylative annulation reaction for the synthesis of 4,5-fused tricyclic 2-quinolones. Chem. Commun..

[39] Mori M., Chiba K., Ohta N., Ban Y. (1979). A novel synthesis of cyclic imides and quinolone by use of palladium catalyzed carbonylation. Heterocycles.

[40] Okuro K., Kai H., Alper H. (1997). Palladium-catalyzed asymmetric cyclocarbonylation of 2-(1-methylvinyl) anilines. Tetrahedron Asymm..

[41] Banwell M.G., Lupton D.W., Ma X., Renner J., Sydnes M.O. (2004). Synthesis of quinolines, 2-quinolones, phenanthridines and 6(5H)-phenanthridinones via palladium[0]-mediated Ullmann cross-coupling of 1-bromo-2-nitroarenes with β-halo-enals, -enones or –esters. Org. Lett..

[42] Teng Y., Suwanarusk R., Ngai M.H., Srinivasan R., Ong A.S.M., Ho B., Laurent R., Chai C.L.L. (2015). An amidation/cyclization approach to the synthesis of *N*-hydroxyquinolinones and their biological evaluation as potential anti-plasmodial, anti-bacterial and iron(II)-chelating agents. Bioorg. Med. Chem. Lett..

[43] Porosa L., Viirre R.D. (2009). Desymmetrization of malonamides via an enantioselective intramolecular Buchwald-Hartwig reaction. Tetrahedron Lett..

[44] Takenaka K., Itoh N., Sasai H. (2009). Enantioselective synthesis of C2-symmetric spirobilactams via Pd-catalyzed intramolecular double N-arylation. Org. Lett..

[45] Ortiz-de-Elguea V., Sotomayor N., Lete E. (2015). Two consecutive palladium(II)-promoted C-H alkenylation reactions for the synthesis of 3-alkenylquinolones. Adv. Synth. Catal..

[46] Manley P.J., Bilodeau M.T. (2004). A new synthesis of naphthyridinones and quinolinones: Palladium-catalyzed amidation of o-carbonyl-substituted aryl halides. Org. Lett..

[47] Inamoto K., Saito T., Hiroya K., Doi T. (2010). Palladium-catalysed intramolecular amidation of C(sp2)-H bonds: Synthesis of 4-aryl-2-quinolinones. J. Org. Chem..

[48] Inamoto K. (2013). Synthesis of heterocyclic compounds through palladium-catalyzed C–H cyclization processes. Chem. Pharm. Bull..

[49] Inamoto K., Kawasaki J., Hiroya K., Kondo Y., Doi T. (2012). Tandem-type Pd(II)-catalyzed oxidative Heck reaction/intramolecular C–H amidation sequence: A novel route to 4-aryl-2-quinolinones. Chem. Commun..

[50] Jia C., Piao D., Kitamura T., Fujiwara Y. (2000). New method for preparation of coumarins and quinolinones via Pd-catalyzed intramolecular hydroarylation of C-C triple bonds. J. Org. Chem..

[51] Kancherla R., Naveen T., Maiti D. (2015). Palladium-catalyzed [3 + 3] annulation between diarylamines and α,β-unsaturated acids through C-H activation: Direct access to 4-substituted 2-quinolinones. Chem. Eur. J..

[52] Queiroz M.J.R.P., Castanheira E.M.S., Lopes T.C.T., Cruz Y.K., Kirsch G. (2007). Synthesis of fluorescent tetracyclic lactams by a “one pot” three steps palladium-catalyzed borylation, Suzuki coupling (BSC) and lactamization DNA and polynucleotides binding studies. J. Photochem. Photobiol. A Chem..

[53] Tsuritani T., Yamamoto Y., Kawasaki M., Mase T. (2009). Novel Approach to 3,4-dihydro-2(1H)-quinolinone derivatives via cyclopropane ring expansion. Org. Lett..

[54] Wu J., Xiang S., Zeng J., Leow M., Liu X.W. (2015). Practical route to 2-quinolinones via a Pd-catalysed C-H bond activation/C-C bond formation/ cyclisation cascade reaction. Org. Lett..

[55] Yan J.X., Li H., Liu X.W., Shi J.L., Wang X., Shi Z.J. (2014). Palladium-catalyzed C(sp3)-*H* activation: A facile method for the synthesis of 3,4-dihydroquinolinone derivatives. Angew. Chem. Int. Ed..

[56] Dong Y., Liu B., Chen P., Liu Q., Wang M. (2014). Palladium-catalyzed C-S activation/aryne insertion/coupling sequence: Synthesis of functionalized 2-quinolinones. Angew. Chem. Int. Ed..

[57] Wang W., Peng X., Qin X., Zhao X., Ma C., Tung C.H., Xu Z. (2015). Synthesis of quinolinones with palladium-oxidative annulation between acrylamides and arynes. J. Org. Chem..

[58] Tadd A.C., Matsuno A., Fielding M.R., Willis M.C. (2009). Cascade palladium-catalyzed alkenyl aminocarbonylation/intramolecular aryl amidation: An annulative synthesis of 2-quinolones. Org. Lett..

[59] Kundu N.G., Mahanty J.S., Das P., Das B. (1993). Synthesis of quinolines and 2,3-dihydro-4(1*H*)-quinolones. Palladium catalysed reaction of o-iodoanilides with acetylenic carbinols. Tetrahedron Lett..

[60] Mahanty J.S., De M., Das P., Kundu N.G. (1997). Palladium catalysed heteroannulation with acetylenic carbinols as synthons—Synthesis of quinolines and 2,3-dihydro-4(1*H*)-quinolones. Tetrahedron.

[61] Pisaneschi F., Sejberg J.J.P., Blain C., Ng W.H., Aboagye E.O., Spivey A.C. (2011). 2-Substituted-2,3-dihydro- -1H-quinolin-4-ones via acid-catalyzed tandem Rupe rearrangement-Donnelly-Farrell ring closure of 2-(3’-hydroxy-propynyl)anilines. Synlett.

[62] Kalinin V.N., Shostakovsky M.V., Ponomaryov A.B. (1992). A new route to 2-aryl-4-quinolones via palladium catalyzed carbonylative coupling of o-iodoanilines with terminal arylacetylenes. Tetrahedron Lett..

[63] Haddad N., Tan J., Farina V. (2006). Convergent synthesis of the quinolone substructure of BILN 2061 via carbonylative Sonogashira coupling/cyclization. J. Org. Chem..

[64] Genelot M., Bendjeriou A., Dufaud V., Djakovitch L. (2009). Optimised procedures for the one-pot selective syntheses of indoxyls and 4-quinolones by a carbonylative Sonogashira/cyclisation sequence. Appl. Catal. A.

[65] Genelot M., Dufaud V., Djakovitch L. (2011). Heterogeneous metallo-organocatalysis for the selective one-pot synthesis of 2-benzylidene-indoxyl and 2-phenyl-4-quinolone. Tetrahedron.

[66] Batail N., Genelot M., Dufaud V., Joucla L., Djakovitch L. (2011). Palladium-based innovative catalytic procedures: Designing new homogeneous and heterogeneous catalysts for the synthesis and functionalisation of *N*-containing heteroaromatic compounds. Catal. Today.

[67] Åkerbladh L., Nordeman P., Wejdemar M., Odell L.R., Larhed M. (2015). Synthesis of 4-Quinolones via a Carbonylative Sonogashira Cross-Coupling Using Molybdenum Hexacarbonyl as a CO Source. J. Org. Chem..

[68] Zhao T., Xu B. (2010). Palladium-catalyzed tandem amination reaction for the synthesis of 4-quinolones. Org. Lett..

[69] Fei X.D., Zhou Z., Li W., Zhu Y.M., Shen J.K. (2012). Buchwald–Hartwig coupling/Michael addition reactions: One-pot synthesis of 1,2-disubstituted 4-quinolones from chalcones and primary amines. Eur. J. Org. Chem..

[70] Sakamoto T., Kondo Y., Uchiyama D., Yamanaka H. (1991). Condensed heteroaromatic ring systems. XIX. Synthesis and reactions of 5-(tributylstannyl)isoxazoles. Tetrahedron.

[71] Sakamoto T., Yasuhara A., Kondo Y., Yamanaka H. (1992). Condensed heteroaromatic ring systems. XX. Palladium-catalyzed carbonylative coupling of iodobenzenes with (*Z*)-1-ethoxy-2-(tributylstannyl)ethane. Chem. Pharm. Bull..

[72] Torii S., Okumoto H., Xu L.H. (1990). A direct approach to 2-substituted 1,4-dihidro-4-oxo-quinoline-3- carboxylates by palladium-catalyzed carbonylative cyclization. Tetrahedron Lett..

[73] Latham E.J., Stanforth S.P. (1996). Synthesis of indoles and quinolones by sequential Wittig and Heck reactions. Chem. Commun..

[74] Latham E.J., Stanforth S.P. (1997). Synthesis of indoles and quinolones by sequential Wittig and Heck reactions. J. Chem. Soc. Perkin Trans..

[75] Costa M., Cà N.D., Gabriele B., Massera C., Salerno G., Soliani M. (2004). Synthesis of 4*H*-3,1-benzoxazines, quinazolin-2-ones and quinolin-4-ones by palladium-catalyzed oxidative carbonylation of 2-ethynylaniline derivatives. J. Org. Chem..

[76] Gabriele B., Salerno G., Costa M. (2004). PdI2-catalyzed synthesis of heterocycles. Synlett.

[77] Alfonsi R., Botta B., Cacchi S., Di Marcotullio L., Fabrizi G., Faedda R., Goggiamani A., Iazzetti A., Mori M. (2017). Design, palladium-catalyzed synthesis and biological investigation of 2-substituted 3-aroylquinolin-4(1*H*)-ones as inhibitors of the Hedgehog signaling pathway. J. Med. Chem..

[78] Okuro K., Alper H. (2012). Palladium-catalyzed intermolecular cyclocarbonylation of 2-iodoanilines with the Michael acceptor, diethyl ethoxycarbonylbutendienoate. J. Org. Chem..

[79] Grigg R., Liu A., Shaw D., Suganthan S., Woodall D.E., Yoganathan G. (2000). Synthesis of quinol-4-ones and chroman-4-ones via a palladium-catalysed cascade carbonylation-allene insertion. Tetrahedron Lett..

[80] Okuro K., Alper H. (1997). Palladium-catalyzed carbonylation of o-iodophenols with allenes. J. Org. Chem..

[81] Wu J., Zhou Y., Wu T., Zhou Y., Chiang C.W., Lei A. (2017). From ketones, amines and carbon monoxide to 4-quinolones: Palladium-catalyzed oxidative carbonylation. Org. Lett..

[82] Huang J., Chen Y., King A.O., Dilmeghani M., Larsen R.D., Faul M.M. (2008). A mild, one-pot synthesis of 4-quinolones via sequential Pd-catalyzed amidation and base-promoted cyclization. Org. Lett..

[83] Park K.Y., Lee J., Park S.J., Heo J.N., Lim H.J. (2015). Regioselective synthesis of 1,2- and 1,4-dihydroquinolines by palladium-catalyzed intramolecular *N*-arylation. Adv. Synth. Catal..

[84] Nemoto T., Fukuda T., Hamada Y. (2006). Efficient synthesis of 3-substituted 2,3-dihydroquinolin-4-ones using a one-pot sequential multi-catalytic process: Pd-catalyzed allylic amination-thiazolium salt-catalyzed Stetter reaction cascade. Tetrahedron Lett..

[85] Barluenga J., Rodríguez F., Fañanás F.J. (2009). Recent advances in the synthesis of indole and quinoline derivatives through cascade reactions. Chem. Asian J..

[86] Solé D., Serrano O. (2007). Palladium-catalyzed intramolecular nucleophilic substitution at the alkoxycarbonyl group. Angew. Chem. Int. Ed..

[87] Neville C.F., Barr S.A., Grundon M.F. (1992). Approaches to the syntheses of dimeric quinolinone alkaloids. Tetrahedron Lett..

[88] Glasnov T.N., Stadlbauer W., Kappe C.O. (2005). Microwave-assisted multistep synthesis of functionalized 4-arylquinolin-2(1H)-ones using palladium-catalyzed cross-coupling chemistry. J. Org. Chem..

[89] Wang Z., Wang B., Wu J. (2007). Diversity-oriented synthesis of functionalized quinolin-2(1*H*)-ones via Pd-catalyzed site-selective cross-coupling reactions. J. Comb. Chem..

[90] Valente S., Kirsch G. (2011). Facile synthesis of 4-acetyl-coumarins, -thiocoumarin and -quinolin-2(1*H*)-one via very high α-regioselective Heck coupling on tosylates. Tetrahedron Lett..

[91] Chattopadhyay S.K., Neogi K., Singha S.K., Dey R. (2008). Sequential Claisen rearrangement and intramolecular Heck reaction as a route to medium-ring oxacycle-fused heterocycles. Synlett.

[92] Majumdar K.C., Nandi R.K., Samanta S., Chattopadhyay B. (2010). Novel synthesis of heterocycle-annulated azocine derivatives of biological relevance by aromatic aza-Claisen rearrangement and intramolecular Heck reaction. Synthesis.

[93] Majumdar K.C., Samanta S., Ghosh T. (2012). Synthesis of coumarin- and quinolone-annulated benzazocinone frameworks by a palladium-catalyzed intramolecular Heck reaction. Synthesis.

[94] Majumdar K.C., Ghosh T., Samanta S. (2011). Facile synthesis of coumarin- and quinolone-annulated benzazoninone derivatives by an intramolecular Heck reaction strategy via 9-exo-trig cyclization. Synthesis.

[95] Majumdar K.C., Mukhopadhyay P.P. (2003). Regioselective synthesis of 2H-benzopyrano[3,2-c]quinolin-7(8*H*)-ones by radical cyclization. Synthesis.

[96] Majumdar K.C., Mukhopadhyay P.P., Basu P.K. (2005). Regioselective synthesis of coumarin and quinolone-annulated spiro heterocycles via aryl radical cyclization. Synthetic Commun..

[97] Majumdar K.C., Pal A.K., Taher A., Debnath P. (2007). Highly effective regioselective method for the synthesis of substituted coumarin- and quinolone-annulated heterocycles using a palladium(0)-catalyzed reaction. Synthesis.

[98] Nolan M.T., Bray J.T.W., Eccles K., Cheung M.S., Lin Z., Lawrence S.E., Whitwood A.C., Fairlamb I.J.S., McGlacken G.P. (2014). Pd-catalysed intramolecular regioselective arylation of 2-pyrones, pyridones, coumarins and quinolones by C-H bond functionalization. Tetrahedron.

[99] Pardo L.M., Prendergast A.M., Nolan M.T., Ó Muimhneacháin E., McGlacken G.P. (2015). Pd/pivalic acid mediated direct arylation of 2-pyrones and related heterocycles. Eur. J. Org. Chem..

[100] Majumdar K.C., Chakravorty S., Shyam P.K., Taher A. (2009). Palladium-mediated Heck reaction to the synthesis of 3-substituted indoles and indolones. Synthesis.

[101] Majumdar K.C., Ganai S., Chattopadhyay B., Ray K. (2011). Palladium-catalyzed one-pot synthesis of pyrrole-annulated coumarin, quinolone and 7-aza-indole derivatives. Synlett.

[102] Payack J.F., Vazquez E., Matty L., Kress M.H., McNamara J. (2005). A concise synthesis of a novel antiangiogenic tyrosine kinase inhibitor. J. Org. Chem..

[103] Fraley M.E., Arrington K.L., Buser C.A., Ciecko P.A., Coll K.E., Fernandes C., Hartman G.D., Hoffman W.F., Lynch J.J., McFall R.C. (2004). Optimization of the indolyl quinolinone class of KDR (VEGFR-2) kinase inhibitors: Effects of 5-amido- and 5-sulphonamido-indolyl groups on pharmacokinetics and hERG binding. Bioorg. Med. Chem. Lett..

[104] Wu J., Zhang L., Sun X. (2005). Synthesis of 3,4-disubstituted quinolin-2(1*H*)-ones via palladium-catalyzed regioselective cross-coupling reactions of 3-bromo-4-trifloxyquinolin-2(1*H*)-one with arylboronic acids. Chem. Lett..

[105] Wang Z., Wu J. (2008). Synthesis of 1*H*-indol-2-yl-(4-aryl)-quinolin-2(1*H*)-ones via Pd-catalyzed regioselective cross-coupling reaction and cyclization. Tetrahedron.

[106] Hashim J., Glasnov T.N., Kremsner J.M., Kappe C.O. (2006). Symmetrical bisquinolones via metal-catalyzed cross-coupling and homocoupling reactions. J. Org. Chem..

[107] Hao F., Asahara H., Nishiwaki N. (2017). Direct and efficient functionalization of the 1-methyl-2-quinolone framework. Proc. Engin..

[108] Majumdar K.C., Mondal S. (2008). A new strategy for the synthesis of coumarin- and quinolone-annulated pyrroles via Pd(0) mediated cross-coupling followed by Cu(I) catalyzed heteroannulation. Tetrahedron Lett..

[109] Majumdar K.C., Ghosh S.K. (1994). Studies on amine oxide rearrangements: Regioselective synthesis of pyrano[3,2-e]indol-7-one. J. Chem. Soc. Perkin Trans..

[110] Majumdar K.C., Biswas P., Jana G.H. (1997). Studies on amine oxide rearrangements: Regioselective synthesis of pyrrolo[3,2-f]quinolin-7-ones. J. Chem. Res..

[111] Majumdar K.C., Chatoppadhyay B., Samanta S. (2009). A short route to the synthesis of pyrrolocoumarin and pyrroloquinolone derivatives by Sonogashira cross-coupling and gold-catalyzed cycloisomerization of acetylenic amines. Synthesis.

[112] Majumdar K.C., De N., Roy B. (2010). Iron/palladium-catalyzed intramolecular hydroamination: An expedient synthesis of pyrrole-annulated coumarin and quinolone derivatives. Synthesis.

[113] Wang Z., Xing X., Xue L., Gao F., Fang L. (2013). Synthesis of 3H-pyrrolo[2,3-c]quinolin-4(5*H*)-ones via Pd-catalyzed cross-coupling reaction and cyclization. Org. Biomol. Chem..

[114] Wang Z., Xue L., He Y., Weng L., Fang L. (2014). Access to functionalized 3*H*-pyrrolo[2,3-c]quinolin-4(5*H*)-ones and thieno[2,3-c]quinolin-4(5*H*)-ones via domino reaction of 4-alkynyl-3-bromoquinolin-2(1*H*)-ones. J. Org. Chem..

[115] Messaoudi S., Audisio D., Brion J.D., Alami M. (2007). Rapid access to 3-(N-substituted)-aminoquinolin-2(1H)-ones using palladium-catalyzed C–N bond coupling reaction. Tetrahedron.

[116] Soussi M.A., Audisio D., Messaoudi S., Provot O., Brion J.D., Alami M. (2011). Palladium-catalyzed coupling of 3-halo-substituted coumarins, chromenes and quinolones with various nitrogen-containing nucleophiles. Eur. J. Org. Chem..

[117] Walsh T.F., Toupence R.B., Young J.R., Huang S.X., Ujjainwalla F., DeVita R.J., Goulet M.T., Wyvratt M.J., Fisher M.H., Lo J.L. (2000). Potent antagonists of gonadotropin releasing hormone receptors derived from quinolone-6-carboxamides. Bioorg. Med. Chem. Lett..

[118] Sahu K.B., Maity A., Mondal S., Paira R., Saha P., Naskar S., Hazra A., Benerjee S., Mondal N.B. (2013). Basic alumina-supported synthesis of aryl-heteroarylmethanes via palladium catalyzed cross-coupling under microwave irradiation. J. Heterocycl. Chem..

[119] Almeida A.I.S., Silva A.M.S., Cavaleiro J.A.S. (2010). Reactivity of 3-iodo-4-quinolones in Heck reactions: Synthesis of novel (*E*)-3-styryl-4-quinolones. Synlett.

[120] Pinto J., Silva V.L.M., Silva A.M.G., Silva A.M.S., Costa J.C.S., Santos L.M.N.B.F., Enes R., Cavaleiro J.A.S., Vicente A.A.M.O.S., Teixeira J.A.C. (2013). Ohmic heating as a new efficient process for organic synthesis in water. Green Chem..

[121] Silva V.L.M., Santos L.M.N.B.F., Silva A.M.S. (2017). Ohmic heating: An emerging concept in organic synthesis. Chem. Eur. J..

[122] Pinto J., Silva V.L.M., Santos L.M.N.B.F., Silva A.M.S. (2016). Synthesis of (*E*)-3-styrylquinolin-4(1*H*)-ones in water by ohmic heating: A comparison with other methodologies. Eur. J. Org. Chem..

[123] Silva V.L.M., Silva A.M.S., Cavaleiro J.A.S. (2010). New synthesis of 2,3-diarylacridin-9(10*H*)-ones and (*E*)-2-phenyl-4-styrylfuro[3,2-c]quinolones. Synlett.

[124] Silva V.L.M., Silva A.M.S. (2014). New synthetic methods for 2,3-diarylacridin-9(10*H*)-one and (*E*)-2-aryl-4-styrylfuro[3,2-c]quinoline derivatives. Tetrahedron.

[125] Jakopović I.P., Kragol G., Forrest A.K., Frydrych C.S.V., Štimac V., Kapić S., Škugor M.M., Ilijaš M., Paljetak H.C., Jelić D. (2010). Synthesis and properties of macrolones characterized by two ether bonds in the linker. Bioorg. Med. Chem..

[126] Joseph B., Béhard A., Lesur B., Guillaumet G. (2003). Convenient synthetic routes to 5-substituted 3-(4-methoxyphenyl)- 4(1*H*)-quinolones. Synlett.

[127] Pain C., Célanire S., Guillaumet G., Joseph B. (2003). Synthesis of 5-substituted 2-(4- or 3-methoxyphenyl)-4(1*H*)-quinolones. Tetrahedron.

[128] Cross R.M., Manetsch R. (2010). Divergent route to access structurally diverse 4-quinolones via mono or sequential cross-couplings. J. Org. Chem..

[129] Mugnaini C., Falciani C., De Rosa M., Brizzi A., Pasquini S., Corelli F. (2011). Regioselective functionalization of quinolin-4(1*H*)-ones via sequential palladium-catalyzed reactions. Tetrahedron.

[130] Felts A.S., Rodriguez A.L., Smith K.A., Engers J.L., Morrison R.D., Byers F.W., Blobaum A.L., Locuson C.W., Chang S., Venable D.F. (2015). Design of 4-oxo-1-aryl-1,4-dihydroquinoline-3-carboxamides as selective negative allosteric modulators of metabotropic glutamate receptor subtype 2. J. Med. Chem..

[131] Pinto J., Silva V.L.M., Silva A.M.G., Santos L.M.N.B.F., Silva A.M.S. (2015). Ohmic heating-assisted synthesis of 3-arylquinolin-4(1*H*)-ones by a reusable and ligand-free Suzuki−Miyaura reaction in water. J. Org. Chem..

[132] Gomes A.T.P.C., Cunha A.C., Domingues M.R.M., Neves M.G.P.M.S., Tomé A.C., Silva A.M.S., Santos F.C., Souza M.C.B.V., Ferreira V.F., Cavaleiro J.A.S. (2011). Synthesis and characterization of new porphyrin/4-quinolone conjugates. Tetrahedron.

[133] Holder J.C., Marziale A.N., Gatti M., Mao B., Stoltz B.M. (2013). Palladium-catalyzed asymmetric conjugate addition of arylboronic acids to heterocyclic acceptors. Chem. Eur. J..

[134] Venkataraman S., Barange D.K., Pal M. (2006). One-pot synthesis of 2-substituted furo[3,2-c]quinolines via tandem coupling-cyclization under Pd/C-copper catalysis. Tetrahedron Lett..

[135] Pal M. (2009). Palladium-catalyzed alkynylation of aryl and hetaryl halides: A journey from conventional palladium complexes or salts to palladium/carbon. Synlett.

[136] Škugor M.M., Štimac V., Palej I., Lugarić D., Paljetak H.Č., Filić D., Modrić M., Dilović I., Gembarovski D., Mutak S. (2010). Synthesis and biological activity of 4′′-O-acyl derivatives of 14- and 15-membered macrolides linked to ω-quinolone-carboxylic unit. Bioorg. Med. Chem..

[137] Kapić S., Paljetak H.Č., Alihodžić S., Antolović R., Haber V.E., Jarvest R.L., Holmes D.J., Broskey J.P., Hunt E. (2010). 6-Alkylquinolone-3-carboxylic acid tethered to macrolides synthesis and antimicrobial profile. Bioorg. Med. Chem..

[138] Layek M., Gajare V., Kalita D., Islam A., Mukkanti K., Pal M. (2009). Pd/C–Cu in coupling-cyclization process: A general synthesis of 2-substituted 6-oxopyrrolo[3,2,1-ij]quinoline derivatives. Tetrahedron Lett..

[139] Laborde E., Leshaski L.E., Kiely J.S. (1990). Palladium-catalyzed intermolecular vinylic arylation of cycloalkenes. Applications to the synthesis of quinolone antibacterials. Tetrahedron Lett..

[140] Kiely J.S., Laborde E., Lesheski L.E., Bucsh R.A. (1991). Synthesis of 7-(alkenyl, cycloalkenyl and 1,2,3,6-tetrahydro-4-pyridinyl)quinolones. J. Heterocycl. Chem..

[141] Reuman M., Daum S.J., Singh B., Wentland M.P., Perni R.B., Pennock P., Carabateas P.M., Gruett M.D., Saindane M.T., Dorff P.H. (1995). Synthesis and antibacterial activity of some novel l-substituted 1,4-dihydro-4-oxo-7-pyridinyl-3-quinolinecarboxylic acids. Potent antistaphylococcal agents. J. Med. Chem..

[142] Wicke L., Engels J.W., Gambari R., Saab A.M. (2013). Synthesis and antiproliferative activity of quinolone nucleosides against the human myelogenous leukemia K-562 cell line. Arch. Pharm. Chem. Life Sci..

[143] Batalha P.N., Gomes A.T.P.C., Forezi L.S.M., Costa L., Souza M.C.B.V., Boechat F.C.S., Ferreira V.F., Almeida A., Faustino M.A.F., Neves M.G.P.M.S. (2015). Synthesis of new porphyrin/4-quinolone conjugates and evaluation of their efficiency in the photoinactivation of Staphylococcus aureus. RSC Adv..

[144] Li M., Li L., Ge H. (2010). Direct C-3-alkenylation of quinolones via palladium-catalyzed C-H functionalization. Adv. Synth. Catal..

[145] Messaoudi S., Brion J.D., Alami M. (2012). Palladium-catalyzed decarboxylative coupling of quinolone-3-carboxylic acids and related heterocyclic carboxylic acids with (hetero)aryl halides. Org. Lett..

[146] Xia C., Yang Y., Wei Z., Yu W., Liao H., Shen C., Zhang P. (2015). Palladium-catalyzed decarboxylative Csp2–Csp2 cross-coupling reactions: An efficient route for synthesis of azaisoflavone derivatives. Catal. Lett..

[147] Xia C., Wei Z., Yang Y., Yu W., Liao H., Shen C., Zhang P. (2016). Palladium-catalyzed thioetherification of quinolone derivatives via decarboxylative C-S cross-couplings. Chem. Asian J..

[148] Schmidt J.P., Li C., Breit B. (2017). Transition-metal-catalyzed regiodivergent and stereoselective access to branched and linear allylated 4-pyridones. Chem. Eur. J..

[149] Han X., Yue Z., Zhang X., He Q., Yang C. (2013). Copper-mediated, palladium-catalyzed cross-coupling of 3-iodochromones, thiochromones and quinolones with ethyl bromodifluoroacetate. J. Org. Chem..

